# Antibiotic pretreatment attenuates liver ischemia–reperfusion injury by Farnesoid X receptor activation

**DOI:** 10.1038/s41419-022-04955-x

**Published:** 2022-05-21

**Authors:** Hanyi Liu, Jinglin Wang, Yitao Ding, Xiaolei Shi, Haozhen Ren

**Affiliations:** 1 Department of Hepatobiliary Surgery, The Affiliated Drum Tower Hospital of Nanjing University Medical School, Nanjing, China; 2 Institute of Hepatobiliary Surgery, Nanjing University, Nanjing, China

**Keywords:** Biochemistry, Molecular biology

## Abstract

Prophylactic antibiotics (Abx) are used before liver surgery, and the influence of antibiotic pretreatment on hepatic ischemia–reperfusion injury (IRI) remains unclear. Hence, we explored the impact of Abx pretreatment on hepatic IRI in the present work. The gut microbiota has an essential role in hepatic bile acid (BA) metabolism, and we assumed that depletion of the gut microbiota could affect the composition of hepatic BAs and affect liver IRI. The IRI model demonstrated that Abx pretreatment attenuated liver IRI by alleviating cell apoptosis, reducing the inflammatory response, and decreasing the recruitment of CCR2+ monocytes. Mechanistically, Abx pretreatment reshaped the gut microbiota, especially decreasing the relative abundance of Firmicutes and increasing the relative abundance of Clostridium, which were related to the transformation of BAs and were consistent with the altered bile acid species (unconjugated BAs, especially UDCA). These altered BAs are known FXR agonists and lead to the activation of the farnesoid X receptor (FXR), which can directly bind to the FXR response element (FXRE) harbored in the TLR4 promoter and further suppress downstream mitogen-activated protein kinase (MAPK) and nuclear kappa B (NF-κB) pathways. Meanwhile, the CCL2–CCR2 axis was also involved in the process of FXR activation, as we confirmed both in vivo and in vitro. Importantly, we proved the importance of FXR in mice and clinical occlusion samples, which were inversely correlated with liver injury. Taken together, our study identified that Abx pretreatment before liver resection was a beneficial event by activating FXR, which might become a potential therapeutic target in treating liver injury.

## Introduction

Liver ischemia–reperfusion injury (IRI) occurs in the surgical removal of part of the liver, transplantation, trauma, and other tissue hypoperfusion diseases [1]. Liver IRI contains two distinctive stages, ischemia and reperfusion. Cellular metabolic disturbances occur during the ischemic stage, while in the reperfusion stage, injury from metabolic disturbances and a robust inflammatory response lead to cytotoxicity directly and indirectly [2]. The main factors affecting hepatic IRI include ischemia time, collateral circulation, the concentration of electrolytes, and gut microbiota. However, the underlying mechanisms of liver IRI remain largely undetermined. Therefore, it is necessary to identify novel therapeutic targets and further explore the pathogenesis of hepatic IRI.

The gut microbiome consists of trillions of mainly colon-restricted, indigenous bacteria [3], and it contributes to all types of diseases [4–6]. However, the specific correlation between gut microbiota and hepatic IRI remains unclear, although several studies have focused on this topic. Antibiotic (Abx) pretreatment for 2 weeks in the “donor” affects Kupffer cell function and improves liver IRI in mouse orthotopic liver transplantation (OLT) [7]. In rat OLT, receivers treated with Abx for 7 days could attenuate liver injury [8], while the underlying mechanisms remain unclear. Recipient Abx pretreatment alleviates liver IRI by decreasing endoplasmic reticulum (ER) stress, enhancing autophagy, and inhibiting inflammation in a PGE2/EP4-dependent manner in mouse OLT [9]. For patients undergoing liver resection and liver transplantation, prophylactic antibiotics are used before surgery. It remains largely unknown whether Abx pretreatment affects hepatic IRI via different pathways.

The liver is the only organ for bile acid (BA) synthesis. Primary BAs are first synthesized from cholesterol in hepatocytes and flow into the duodenum during ingestion of food. The majority of BAs are reabsorbed in the terminal ileum and return to the liver. The remaining BAs pass into the colon, where they are subjected to bacterial metabolism [10]. Therefore, depletion of gut bacteria by broad-spectrum antibiotics can affect the metabolism of hepatic BAs, leading to different outcomes of liver diseases. As a recognized BA receptor, farnesoid X receptor (FXR) (also called NR1H4) functions as a ligand-regulated transcription factor that mediates the expression of different genes involved in cholesterol/BA metabolism, liver gluconeogenesis, and lipogenesis [11]. Interestingly, FXR is also critical in liver IRI. It has been reported that FXR activation induced by GW4064 can alleviate liver IRI by reducing the expression of small heterodimer partner (SHP) in Kupffer cells [12]. In small intestine and kidney IRI models, FXR activation by GW4064 and 6α-ECDCA can attenuate local inflammatory responses and alleviate IRI [13, 14].

Toll-like receptor (TLR) signaling, especially TLR4 signaling, plays a critical role in liver IRI [15]. TLR4 is a pattern recognition receptor that functions in innate immunity and is expressed in multiple innate immune cells and hepatocytes [16, 17]. TLR4 signaling includes two pathways that are dependent on MyD88, and the nuclear kappa B (NF-κB) and mitogen-activated protein kinase (MAPK) signaling pathways are downstream pathways of TLR4 signaling, which are both fundamental in the roles of liver IRI [18, 19]. Chemokines are also critical in the development of IRI. Chemokines and corresponding receptors are important in coordinating sequential immune cells into injured areas in the liver, leading to inflammatory responses to specific triggers.

In the current study, we explored the mechanism behind Abx pretreatment against hepatic IRI and found that FXR might be a therapeutic target in liver IRI. Abx pretreatment reshaped the gut microbiota, decreased the relative abundance of Firmicutes, increased the relative abundance of Clostridium, increased the levels of unconjugated BAs, especially the levels of UDCA, and activated liver FXR. FXR activation decreased the expression of TLR4 by binding directly to the promoter region of TLR4, and downstream NF-κB and MAPK signaling pathways were both depressed in Abx-pretreated IRI mice or GW4064-treated hepatocytes. Moreover, the CCL2–CCR2 axis was involved in this process. In addition, we also demonstrated changes in FXR signaling in clinical liver resection samples with different ischemia durations. Collectively, we, for the first time, investigated the relationship between Abx pretreatment and FXR signaling in a mouse IRI model and provided a potential target to treat IRI in liver surgery.

## Materials and methods

### Animals

Briefly, 6–8-week-old male C57BL/6J mice were provided by the Animal Center of the Affiliated Drum Tower Hospital of Nanjing University Medical School. All animals were bred in a specific pathogen-free facility. All animal-related protocols were authorized by the Institutional Animal Care and Use Committee of Nanjing University, China, which complied with the NIH Guide for the Care and Use of Laboratory Animals.

Four-week-old male C57BL/6 mice were orally gavaged with antibiotics (1 g/L ampicillin; 0.5 g/L vancomycin; 1 g/L metronidazole; and 1 g/L neomycin) at a total dose of 20 mL/kg/day for 4 weeks as previously described [7] with some modifications. Control mice were gavaged with the same dose of saline for 4 weeks.

To achieve liver-specific FXR knockdown in the mouse model, 4-week-old male C57BL/6 mice were transfected with 10^11^ pfu AAV8-delivered short hairpin RNA (shRNA) (GeneChem), which consists of a liver-specific thyroxine-binding globulin (TBG) promoter and a GFP tag. shFXR had the following target sequence: GGAGAGTGAATGATCACAA. On the second day after tail vein injection, all mice were orally gavaged with antibiotics for 4 weeks.

All mice were randomly assigned to the experiments.

### Human liver samples

Liver specimens were harvested from subjects with benign focal hepatic lesions who underwent liver surgery between April 2020 and April 2021 at Affiliated Drum Tower Hospital, Medical School of Nanjing University. The principal diagnosis of these patients included focal nodular hyperplasia (FNH), hemangioma, hepatolithiasis, and angiomyolipoma, without a history of autoimmune disorders, hepatitis, malignancy, immune deficiencies, or HIV infection. Liver samples were collected and preserved immediately. All patients involved were informed and signed written consent forms. All human experiments were authorized by the human ethics committees of the Affiliated Drum Tower Hospital, Medical School of Nanjing University, following the Declaration of Helsinki.

### Liver IRI model

The hepatic warm IRI model was described previously with slight modifications [20]. In brief, isoflurane in combination with chloral hydrate was used for anesthesia, and the portal vein and arterial blood supply to the left and median liver lobes were blocked using an atraumatic clip. The clip was removed after 90 min of interruption. Mice were euthanized after 6 h of reperfusion with high-dose isoflurane. Serum samples were collected and immediately analyzed by an automatic analyzer (Fuji, Tokyo, Japan) for serum alanine aminotransferase (ALT), aspartate aminotransferase (AST), and lactate dehydrogenase (LDH). In addition, liver samples were sectioned and subjected to fixation or snap-frozen.

### Histology and immunohistochemical (IHC) staining

Paraffin-embedded liver tissue was subjected to hematoxylin and eosin (H&E) staining. Liver injury was assessed by Suzuki’s et al. criteria [21] by three different pathologists who were blinded to groups in the Department of Pathology, Affiliated Drum Tower Hospital, Medical School of Nanjing University. For IHC assays, the sections were subjected to an antigen retrieval process and then incubated with antibodies against CD68 (Abcam Cambridge, MA, USA), Ly6G (Abcam), CCR2 (Abcam), CCL2 (Proteintech, Wuhan, China), and FXR (Santa Cruz Biotechnology, Santa Cruz, CA, USA) overnight at 4 °C. This was followed by incubation with horseradish peroxidase-conjugated secondary antibodies. Immunoreactive cells were incubated with DAB (diaminobenzidine) substrate. At least three images were taken from random fields for each stained section, and each experimental group contained at least three mice.

### Fecal pellet collection and 16S rDNA Sequencing

24 h after the last gavage, the mouse was placed in a clean cage covered with sterile filter paper, and the fecal sample were collected immediately into sterile centrifuge tube after excretion. Each sample weighed more than 200 mg. After the collection of each sample, the filter paper should be replaced and the collected sterile centrifuge should be placed on liquid nitrogen immediately. After all samples were collected, the samples were shipped on dry ice to the company for further testing.

Fecal 16S rDNA assays were performed by Shanghai Biotree Biotech Co., Ltd. (Shanghai, China). Briefly, microbial DNA was extracted using a QIAamp Fast DNA Stool Mini Kit (Qiagen, CA, United States) based on the recommended protocol. PCR amplification targeted the hypervariable V3–V4 region of the 16S rDNA, followed by purification and quantification. Purified amplicons were sequenced on the Illumina NovaSeq platform, and the data were further analyzed.

### Targeted BA analysis using UHPLC–MS/MS

The content of endogenous BAs in the liver was determined by Shanghai Biotree Biotech Co., Ltd. (Shanghai, China). Briefly, individual liver specimens were precisely weighed and extracted with extraction solution (acetonitrile-methanol-water, 2:2:1, 0.1% formic acid contained and mixed with isotopically labeled standard), and the resulting supernatants were subjected to UHPLC–MS/MS analysis after centrifugation. UHPLC separation was conducted on an Agilent 1290 Infinity series UHPLC System (Agilent Technologies, USA). A series of calibration standard solutions were previously detected.

### Isolation of hepatic nonparenchymal cells (NPCs)

Hepatic NPCs were isolated as previously described [22]. In brief, mice were anesthetized, a 20-G catheter was inserted into the superior vena cava, the inferior vena cava was blocked, and the portal vein was cut to perfuse liver tissues. Prewarmed Hank’s balanced salt solution (HBSS) was used to perfuse liver tissues, followed by incubation with digestion buffer [1 X HBSS containing 0.4% collagenase (Sigma-Aldrich, St. Louis, MO, USA), 1.25 mM CaCl2, 4 mM MgSO_4_, and 10 mM HEPES]. The left and median liver lobes were used to make single-cell suspensions using a 70-μm cell strainer (BD Falcon, Bedford, MA, USA), and the cells were subsequently centrifuged at 700 × *g* for 5 min at 4 °C. The cell pellet was redistributed in 15 mL of 35% Percoll (Sigma-Aldrich, St. Louis, MO, USA) containing 50 U/mL heparin, followed by centrifugation at 500 × *g* for 15 min. The resulting cell pellet was harvested and redistributed in 1.5 mL of red blood cell lysis buffer (Sigma-Aldrich, St. Louis, MO, USA) for 5 min. HBSS containing 0.5% fetal bovine serum (FBS) was used to wash the cells. Finally, the cell pellet was redistributed in 100 µL of HBSS containing 2% FBS for cell counting and antibody staining. The number of total NPCs was normalized according to the liver weight for statistical analysis.

### Flow cytometry

Briefly, 10^6^ hepatic NPCs were incubated with anti-mouse CD16/32 antibody (clone 93, Biolegend, San Diego, CA, USA) for 10 min to minimize nonspecific binding. Cells were incubated with a PerCP-conjugated anti-CD45 antibody (clone 30-F11, Biolegend), an APC-labeled anti-F4/80 antibody (clone BM8, Biolegend), a FITC-conjugated anti-CD11b antibody (clone M1/70, Biolegend), a PE-Cyanine7-conjugated anti-Ly6C antibody (clone HK1.4, Biolegend), and a PE-conjugated anti-CCR2 antibody (clone SA203G11, Biolegend) or incubated with a PerCP-conjugated anti-CD45 antibody (clone 30-F11, Biolegend), an APC-conjugated anti-CD3 antibody (clone 17A2, Biolegend), a PE-Cyanine7-conjugated anti-NK1.1 (clone PK136, Biolegend), a PE-conjugated anti-CD4 (clone GK1.5, Biolegend), and a FITC-conjugated anti-CD8a (clone 53-6.7, Biolegend). The results were collected by a BD FACSAria III Flow Cytometer (BD Bioscience, San Jose, CA, USA) and analyzed using FlowJo 10.4 software (BD Bioscience).

After the cells were treated with hypoxia-reoxygenation (H/R), apoptosis was assessed by an Annexin V-FITC/PI Apoptosis Detection Kit (Vazyme Biotech, Nanjing, China) based on the manufacturer’s protocols.

### Cell culture and H/R model

AML12 (mouse liver cell line) cell lines were purchased from ATCC (Manassas, VA, USA). AML12 cells were cultured in DMEM/F12 medium mixed with 10% FBS, 100 U/mL penicillin, 100 mg/mL streptomycin, 1% insulin-transferrin-sodium selenite (ITS, Sigma-Aldrich, St. Louis, MO, USA), and dexamethasone (40 ng/mL, Sigma-Aldrich). Mycoplasma contamination was conducted every 3 months for all cells and consistently tested negative.

Mouse primary hepatocytes were isolated based on a previous protocol [23]. Briefly, prewarmed solution 1 (Krebs Ringer with glucose and EGTA) and solution 2 (Krebs Ringer with glucose, CaCl_2_ and type IV collagenase) were slowly perfused the liver sequentially from the portal vein. After cell counting, cells were seeded at a density of 5 × 10^5^ cells per well in a 6-well plate, which were precoated with rat-tail collagen I (Sigma-Aldrich). Mouse primary hepatocytes were cultured in DMEM supplemented with 4.5 g/L glucose, 10% fetal bovine serum, 1 mM dexamethasone and 0.1 mM insulin.

To assess H/R injury in vitro, cells were maintained in a hypoxic gas mixture (5% CO_2_, 94% N_2_, and 1% O_2_) without FBS for 12 h at 37 °C. For reoxygenation, the culture medium was changed to medium supplemented with 10% FBS in a 5% CO_2_ incubator, and the cells were incubated for 4 h as previously described [24].

### Enzyme-linked immunosorbent assay (ELISA)

The levels of IL-1β (MultiSciences, Hangzhou, China) in mouse serum were determined using a commercial ELISA kit based on the manufacturer’s protocols.

### siRNA transfection

The siRNA sequence of mouse FXR was GGAGAGTGAATGATCACAA. The siRNA sequence of mouse TLR4 was as follows: CAATCTGACGAACCTAGTA. Cells were transiently transfected with siRNA using Polyplus reagent (Polyplus, Illkirch, France) based on the manufacturer’s protocols.

### Western blotting analysis

Western blotting analysis was carried out as previously described [25]. The following antibodies were used: anti-Bcl-2 (#ab182858), anti-Bax (#ab182733), anti-Bcl-xl (#ab32370), anti-p-P65 (#ab76302), anti-P65 (#ab32536), anti-p-IκBα (#ab133462), anti-IκBα (#ab32518) (Abcam Cambridge, MA, USA), anti-FXR (#sc-25309), anti-TLR4 (#sc-293072) (Santa Cruz Biotechnology, Santa Cruz, CA, USA), anti-p-ERK1/2 (#4370), anti-ERK1/2 (#4695), anti-p-P38 (#4511), anti-P38 (#8690), anti-p-JNK1/2 (#4668), anti-JNK1/2 (#9252), anti-CCL2 (#66272-1-Ig), and anti-β-actin (#66009-1-Ig) (Proteintech, Wuhan, China).

### Quantitative real-time polymerase chain reaction (qRT-PCR)

Total RNA from liver tissues, ileum tissues, and hepatocytes was extracted using TRIzol^TM^ (Life Technologies, Carlsbad, CA, USA) reagent based on the manufacturer’s protocols. Complementary DNA (cDNA) was synthesized with HiScript II RT SuperMix for qPCR + gDNA wiper (Vazyme Biotech, Nanjing, China). The mRNA level of target genes was quantified by qRT-PCR using ChamQ SYBR qPCR Master Mix (Vazyme Biotech, Nanjing, China) on an ABI PRISM 7500 Real-Time PCR System (Applied Biosystems, Foster City, CA, USA). The primers used for qRT-PCR are presented in Supplementary Table S1.

### TUNEL apoptosis assays

A TUNEL BrightGreen Apoptosis Detection Kit (Vazyme Biotech, Nanjing, China) was used based on the manufacturer’s protocols. Briefly, frozen liver sections were dried at room temperature for 15 min and then fixed in 4% paraformaldehyde (diluted in PBS) for 30 min. Sections were incubated with 0.2% Triton X-100 (diluted in PBS) for 15 min and equilibrated with equilibration buffer (diluted in ddH_2_O) for 20 min, followed by incubation with 50 μL TdT reaction mixture at 37 °C for 60 min and protected from light. Subsequently, the sections were counterstained with DAPI and immediately examined under an immunofluorescence microscope.

### Luciferase reporter assay

The predicted mouse TLR4 promoter sequence was cloned into the luciferase reporter vector pGL3-basic. Mouse FXR (mFXR) overexpression plasmids were constructed based on the pcDNA3.1 vector. A dual-luciferase assay was performed by cotransfecting pcDNA3.1-mFXR, pGL3-mTLR4-prom, and pGL4.75 plasmids into AML12 cells and mouse primary hepatocytes. The control vector pGL4.75 was employed to normalize firefly luciferase activity. Luciferase assays were performed with a Dual-Luciferase Reporter Assay Kit (Vazyme Biotech, Nanjing, China) based on the manufacturer’s protocols. Transient transfections were performed with Polyplus reagent (Polyplus, Illkirch, France) based on the manufacturer’s protocols.

### Chromatin immunoprecipitation assay

The ChIP assay was constructed as described previously [26]. Briefly, control or FXR overexpression plasmids were transfected into AML12 cells and mouse primary hepatocytes for 48 h and crosslinked with 1% paraformaldehyde. The crosslinking was stopped after 10 min with glycine. Cells were then washed and lysed with cold PBS supplemented with protease inhibitor. The lysates were sonicated for 15 s and interposed with 30 s pauses, which was repeated 5 times. The DNA fragments were immunoprecipitated with 5 μg of anti-FXR antibodies or IgG (Santa Cruz Biotechnology). The result was confirmed by qRT-PCR. The following primers were used: 5′-TTTGGACAAGGTGATCTGGTGTT-3′ (forward) and 5′-AAATTCAAGTCATAGGACATGGCAC-3′ (reverse).

### Statistical analysis

Statistical analysis was conducted using GraphPad Prism 6.01 software (San Diego, CA, USA). All data are expressed as the means ± SEM. All data were first tested for normality via the Kolmogorov–Smirnov test. Normally distributed data were compared by Student’s *t* test. Otherwise, the Wilcoxon rank-sum test was applied. One-way analysis of variance was adopted to compare differences among multiple groups. *P* values <0.05 were considered statistically significant.

## Results

### Abx pretreatment protects the liver from IRI

**Fig. 1:**
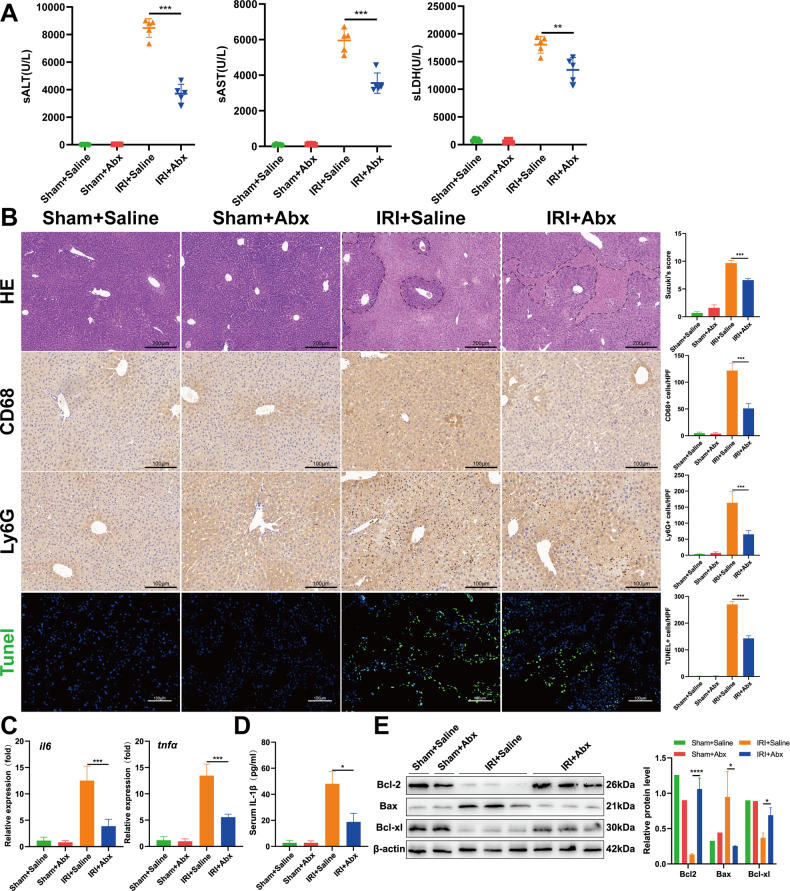
Abx pretreatment protects the liver from IRI. **A** The levels of serum ALT, AST, and LDH in saline- and Abx-treated mice were determined after 90 min of ischemia and 6 h of reperfusion. **B** Representative H&E, CD68, Ly6G, and TUNEL staining of liver sections after IRI. Scale bars, 200, 100, 100, 100 μm. **C** The expression of IL-6 and TNF-α at the mRNA level in different groups after IRI. (*n* = 5 per group) (**D**) Serum IL-1β levels in different groups after IRI. **E** The expression levels of Bcl-2, Bax, and Bcl-xl at the protein level in each group. Data are the mean ± SEM, ^∗^*p* < 0.05; ^∗∗^*p* < 0.01; ^∗∗∗^*p* < 0.001 by unpaired Student’s *t* test.

To study the role of gut flora in hepatic IRI, mice were orally gavaged with saline or broad-spectrum antibiotics for 4 weeks, followed by IRI modeling. Compared with the IRI + saline group, the Abx-treated mice showed significantly attenuated IRI after modeling, confirmed by reduced levels of serum ALT, AST, and LDH (Fig. 1A), and the liver damage area in H&E staining and Suzuki score was also decreased (Fig. 1B). Moreover, Abx pretreatment reduced the accumulation of CD68+ (Fig. 1B) macrophages and Ly6G+ (Fig. 1B) neutrophils in ischemic livers compared with the saline-treated controls. Meanwhile, we also detected the levels of the cytokines IL-6, TNF-α, and IL-1β in the liver (Fig. 1C) and serum (Fig. 1D) and found that Abx-treated mice exhibited a decreased inflammatory response compared with the saline group after IRI. To assess the apoptosis levels in the liver, we used TUNEL staining (Fig. 1B) and Western blotting analysis (Fig. 1E) and proved that Abx pretreatment remarkably reduced apoptosis levels. These results indicated that Abx pretreatment could protect against IRI-induced damage.

### The CCL2–CCR2 axis is involved in the Abx-mediated protection of the liver IRI model

**Fig. 2:**
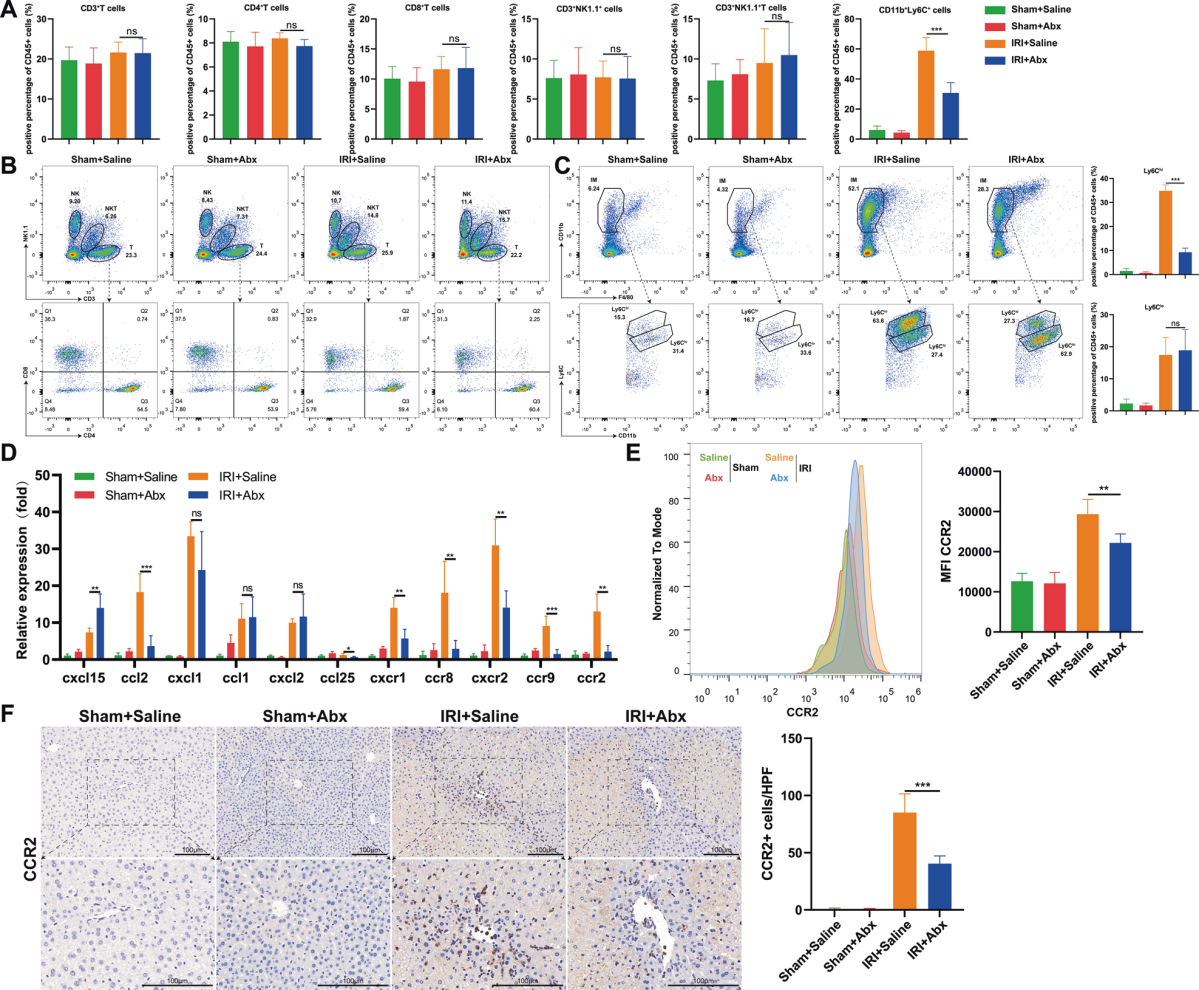
Abx pretreatment decreases the recruitment of CCR2+ monocytes in hepatic IRI. **A** The proportion of NPC cells in the liver after sham and IRI modeling, including CD3+ cells, CD4+ T cells, CD8+ T cells, NK cells, NKT cells, and IMs. **B**, **C** Representative flow cytometry images of different NPC cell proportions in the liver after sham and IRI modeling and quantitative histogram of two subsets of IMs (Ly6C^hi^ and Ly6C^lo^). **D** The expression of chemokines and corresponding receptors at the mRNA level in liver tissues after sham and IRI modeling in each group. **E** Average fluorescence intensity of CCR2+ monocytes in each group (gated on CD45+CD11b+ cells). **F** Representative images of CCR2+ monocytes in liver sections (*n* = 5 per group). Scale bars, 100 μm. Data are the mean ± SEM, ^∗^*p* < 0.05; ^∗∗^*p* < 0.01; ^∗∗∗^*p* < 0.001 by unpaired Student’s *t* test.

The innate immune system is essential in the process of liver IRI. CD4+ T cells, Kupffer cells, neutrophils, NK cells, and natural killer T (NKT) cells have been identified as crucial cells in the pathogenesis of IRI [2, 27, 28]. To investigate whether Abx pretreatment had an impact on the composition of innate immune cells and thus affected liver IRI outcomes, flow cytometry was used to characterize the hepatic cell population. After IRI modeling, the proportions of CD3+ T cells, CD4+ T cells, CD8+ T cells, NK cells, and NKT cells were not different between the two groups, while the proportion of hepatic infiltrating macrophages (IMs) (CD11b+Ly6C+) was significantly decreased in the Abx-treated group (Fig. 2A). IMs were further categorized into two subgroups based on the expression of Ly6C (Ly6C^hi^ and Ly6C^lo^). Figure 2C shows that the reduction in IMs after Abx pretreatment was mainly attributed to the decrease in the Ly6C^hi^ subset, which has been identified as a proinflammatory phenotype of monocytes [29]. Chemokines, such as CXCL1, CXCL2, and CXCL8, are secreted by activated innate immune cells or injured parenchymal cells in a paracrine way to attract neutrophils or monocytes for hepatic accumulation. As we mentioned before, among these chemokines, CCL2 seemed to be especially important for the migration of proinflammatory monocytes, which usually have high expression of Ly6C [30]. Therefore, we detected a series of chemokines and relevant receptors in the Abx pretreatment and saline control groups after IRI, and the CCL2–CCR2 axis seemed to show the most prominent change (Fig. 2D), as evidenced by the quantification of CCR2 + monocytes (gated on CD45+CD11b+ cells) using flow cytometry (Fig. 2E). IHC staining demonstrated that the recruited CCR2+ monocytes were obviously decreased around the central vein in the injured area after Abx pretreatment (Fig. 2F). These results indicated that the decreased recruitment of CCR2+ monocytes was involved in the Abx-mediated protection of the liver IRI model.

### Abx pretreatment reshapes gut microbiota, alters the composition of liver BAs and activates liver FXR

**Fig. 3:**
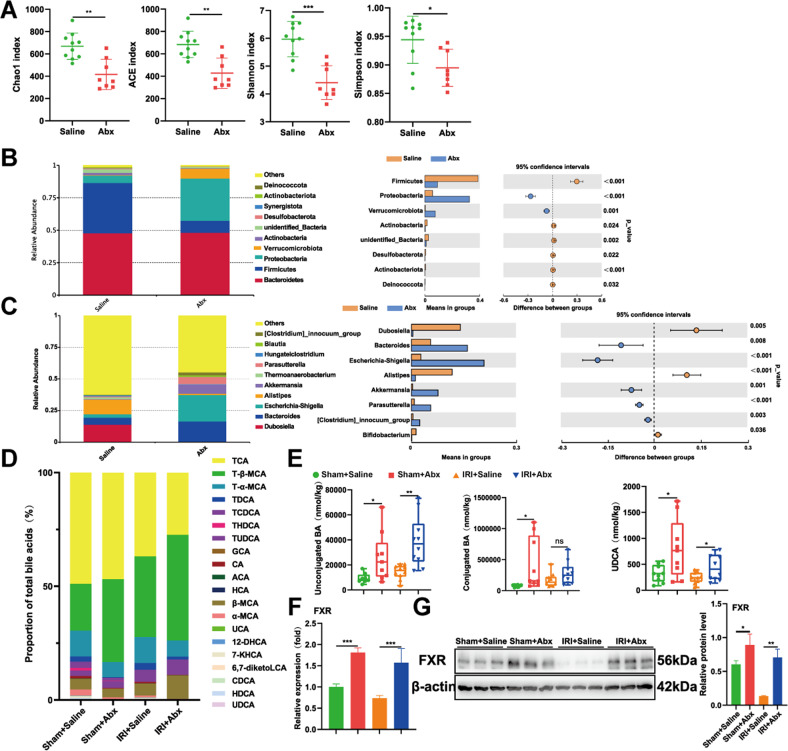
Abx pretreatment reshapes gut microbiota, alters the composition of liver BAs and activates liver FXR. **A** α-diversity index in the two groups. **B** Relative bacterial abundances at the phylum level in the two groups. **C** Relative bacterial abundances at the genus level in the two groups. **D** The ratio of different BAs in the liver. **E** Unconjugated BA, conjugated BA and UDCA levels in the liver. **F**, **G** mRNA and protein levels of FXR in different groups. Scale bars, 100 μm. Data are the mean ± SEM, ^∗^*p* < 0.05; ^∗∗^*p* < 0.01; ^∗∗∗^*p* < 0.001 by Wilcoxon rank-sum test (**A**–**C**) and unpaired Student’s *t* test (**E**–**G**).

To further investigate the mechanism behind the Abx-mediated protective role in liver IRI, we analyzed the changes in gut microbiota after Abx pretreatment by 16S rDNA sequencing. The α-diversity indices (Chao1 index, ACE index, Shannon index, Simpson index) in Fig. 3A proved that Abx-treated mice showed a significant decrease in community richness and diversity. To assess the composition of bacteria in different groups, we investigated the taxonomic profile of bacteria at the phylum and genus levels. Compared with the saline group, Abx-treated mice showed no difference in the relative abundance of Bacteroidetes and a large decrease in Firmicutes (Fig. 3B). At the genus level, Abx pretreatment significantly increased the relative abundance of Bacteroides and Clostridium. Taken together, these changed bacteria are involved in the formation of secondary BAs and bile salt hydrolase (BSH) activity, indicating that Abx pretreatment might influence BA profiles in mice. Therefore, we detected a panel of BAs in the liver using UHPLC–MS. Figure 3D reveals that after Abx pretreatment, the liver showed a different composition of BAs. Microbial attenuation increased the levels of unconjugated BAs in both the sham + Abx group and IRI + Abx group, while conjugated BAs showed no difference between the IRI + Saline group and IRI + Abx group (Fig. 3E). Unconjugated BAs serve as high affinity ligand agonizts of FXR, and compared to conjugated BAs, it is well recognized that unconjugated BAs have greater potential to activate FXR [31]. Thus, we screened all endogenous high affinity ligand agonizts of FXR and found that UDCA was obviously upregulated after Abx pretreatment (Fig. 3E), which was consistent with Clostridium (Fig. 3C), the main metabolic bacteria in the formation of UDCA. These results indicated that Abx pretreatment reshaped the gut microbiota, changed liver BA metabolism and further activated liver FXR, as evidenced by qPCR and Western blotting analysis (Fig. 3F, G).

### Liver-specific FXR knockdown abolishes the Abx-mediated protective effect of hepatic IRI

**Fig. 4:**
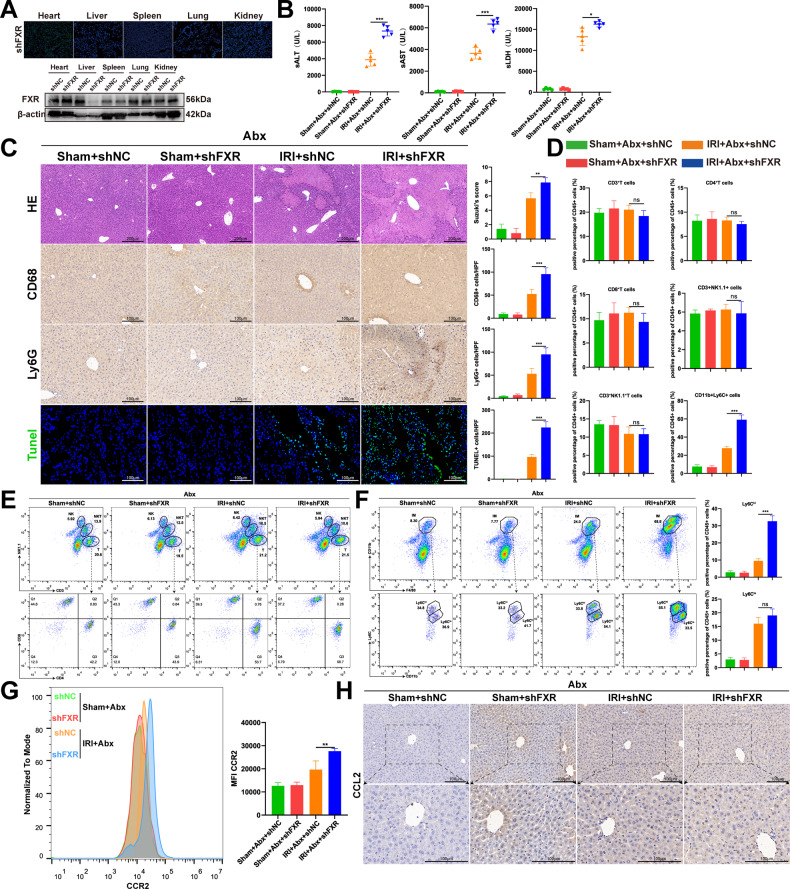
Liver-specific FXR inhibition abolishes the Abx-mediated protective effect of hepatic IRI. **A** Frozen sections and protein levels confirmed liver-specific inhibition of FXR. **B** The levels of serum ALT, AST, and LDH in AAV8 shNC- and AAV8 shFXR-injected mice were measured after IRI. **C** Representative H&E, CD68, Ly6G, and TUNEL staining of liver sections after IRI. Scale bars, 200, 100, 100, 100 μm. **D** The proportion of NPC cells in the liver after sham and IRI modeling, including CD3+ cells, CD4+ T cells, CD8+ T cells, NK cells, NKT cells, and IMs. **E**, **F** Representative flow cytometry images of different NPC cell proportions in the liver after sham and IRI modeling and quantitative histogram of two subsets of IMs (Ly6C^hi^ and Ly6C^lo^). **G** Average fluorescence intensity of CCR2+ monocytes in each group (gated on CD45+ CD11b+ cells). **H** Representative CCL2 IHC staining of liver sections with or without IRI in AAV8 shNC- and AAV8 shFXR-injected mice. (*n* = 5 per group). Data are the mean ± SEM, ^∗^*p* < 0.05; ^∗∗^*p* < 0.01; ^∗∗∗^*p* < 0.001 by unpaired Student’s *t* test.

To test whether FXR activation was the key event in the Abx-mediated protective role of liver IRI, mice were transfected in vivo with adeno-associated virus 8 (AAV8) by the tail vein to knockdown FXR (shFXR). Figure 4A confirmed that transfection with shFXR AAV8 could specifically target the liver and knockdown liver FXR, and mice injected with the corresponding AAV8 empty virus (shNC) were considered the negative control. Mice injected with shFXR AAV8 and shNC AAV8 were both orally gavaged with Abx for 4 weeks, followed by IRI or sham modeling. We observed that shFXR AAV8 injection abolished the Abx-mediated protective role of liver IRI, as confirmed by increased levels of serum ALT, AST, and LDH (Fig. 4B) and increased liver damage area and Suzuki score (Fig. 4C). Moreover, shFXR AAV8 injection also aggravated the inflammatory response, as evidenced by accelerated infiltrating immune cells (Fig. 4C). In addition, shFXR AAV8 injection increased apoptosis levels, as evidenced by the increased number of TUNEL-positive cells (Fig. 4C), suggesting that FXR activation was the key event in the Abx-mediated protective role of hepatic IRI. To investigate whether FXR activation influenced the proportion of hepatic innate immune cells, especially IMs and CCR2+ monocytes, we detected the proportion of innate immune cells by flow cytometry. Figure 4F, G confirmed that FXR knockdown could upregulate the proportion of hepatic IMs and CCR2+ monocytes in Abx-treated IRI mice. IHC staining demonstrated that FXR knockdown could accelerate the secretion of CCL2 (Fig. 2H) in Abx-treated IRI mice. These data suggested that the Abx-mediated protective role of hepatic IRI was mainly due to the activation of liver FXR.

### FXR regulates MAPK and NF-κB signaling by suppressing TLR4 both in vivo and in vitro

**Fig. 5:**
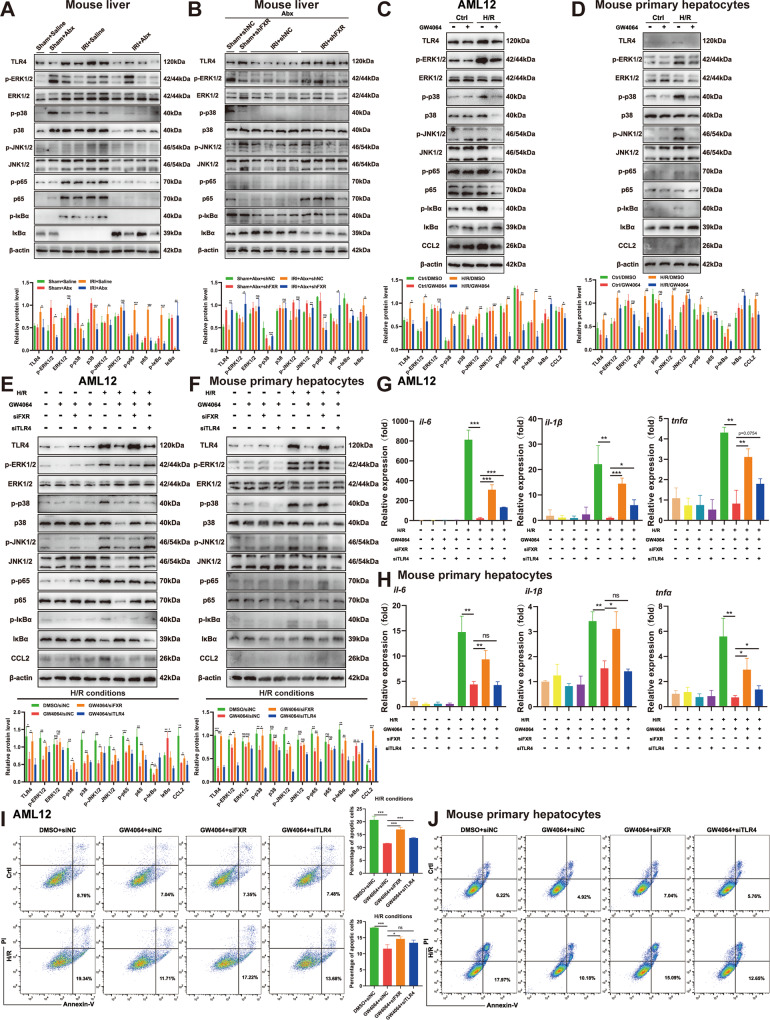
FXR regulates MAPK and NF-κB signaling by suppressing TLR4 both in vivo and in vitro. **A**, **B** Western blotting analysis of hepatic TLR4, MAPK, and NF-κB pathways in saline-, Abx-treated or Abx/shNC-, or Abx/shFXR-injected mice. **C**, **D** Western blotting analysis of the TLR4, MAPK, and NF-κB pathways and CCL2 in DMSO- and GW4064-treated AML12 cells or mouse primary hepatocytes after H/R. **E**, **F** Western blotting analysis of the TLR4, MAPK and NF-κB pathways and CCL2 in the H/R + GW4064 + FXR siRNA and H/R + GW4064 + TLR4 siRNA groups in AML12 cells or mouse primary hepatocytes. **G**, **H** The expression of IL-6, TNF-α, and IL-1β at the mRNA level in AML12 cells or mouse primary hepatocytes treated with H/R, GW4064, FXR siRNA, or TLR4 siRNA singly or in combination. **I**, **J** Apoptosis levels of AML12 cells or mouse primary hepatocytes treated with H/R, GW4064, FXR siRNA, or TLR4 siRNA singly or in combination (*n* = 3–4 per group). Data are the mean ± SEM, ^∗^*p* < 0.05; ^∗∗^*p* < 0.01; ^∗∗∗^*p* < 0.001 by unpaired Student’s *t* test.

TLR4 is crucial in the pathogenesis of liver IRI. To further investigate the molecular mechanism of FXR activation in liver IRI, we analyzed the expression of TLR4 and its downstream MAPK (including p-ERK1/2, ERK1/2, p-P38, P38, p-JNK1/2, and JNK1/2) and NF-κB (including p-P65, P65, p-IκBα, and IκBα) family members at the protein level using Western blotting analysis. Figure 5A shows that the expression levels of TLR4, p-ERK1/2, p-P38 and p-JNK1/2 were remarkably elevated in the saline group after IRI, while Abx pretreatment dramatically decreased the expression levels of TLR4, p-ERK1/2, p-P38 and p-JNK1/2. Figure 5B confirmed that FXR inhibition could reverse the decrease in protein levels in mice pretreated with Abx. To confirm the relationship between FXR activation and TLR4 expression, the inflammatory response, and cell apoptosis, in vitro experiments were carried out in AML12 cell line and mouse primary hepatocytes. We imitated IRI in vitro using the H/R model as described previously [24]. As expected, the H/R model activated MAPK and NF-κB signaling and upregulated the expression of TLR4 both in AML12 cells and mouse primary hepatocytes, while such activation and upregulation were abrogated after administration of GW4064, a selective FXR agonist (Fig. 5C, D). The expression of CCL2 has been confirmed to be related to MAPK and NF-κB signaling [32], and we proved it in both AML12 cells and mouse primary hepatocytes (Fig. 5C, D). Figure 5G–J show that the H/R model promoted the inflammatory response and cell apoptosis in AML12 cells and mouse primary hepatocytes, while administration of GW4064 reversed this process. To investigate whether FXR regulates the TLR4-mediated MAPK and NF-κB signaling pathways by directly modulating TLR4 expression, we used siRNA to deplete FXR in AML12 cells and mouse primary hepatocytes. Depletion of FXR abolished the suppression of the MAPK and NF-κB signaling pathways and downregulation of TLR4 and CCL2 (Fig. 5E, F), as well as the inflammatory response (Fig. 5G, H) and cell apoptosis (Fig. 5I, J), after administration of GW4064 in the H/R model.

**Fig. 6:**
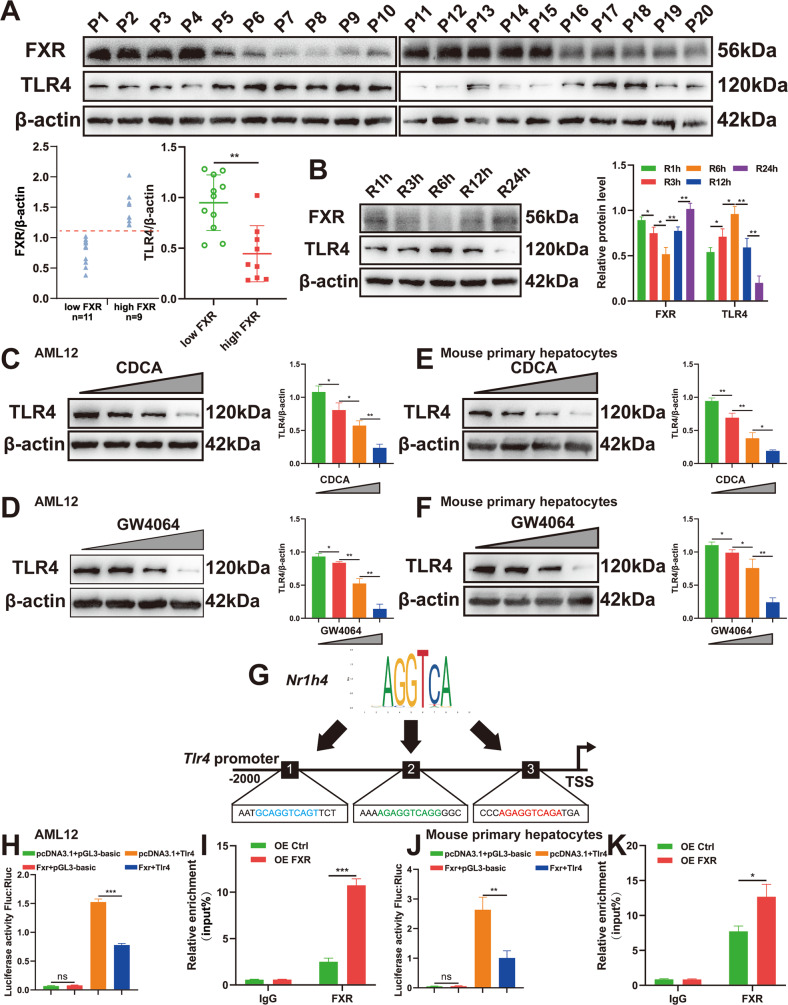
FXR suppresses TLR4 transcription by directly binding to the FXRE located in the TLR4 promoter region. **A** Western blotting analysis of FXR in 20 clinical benign surgical liver samples. **B** The expression of hepatic FXR and TLR4 at the protein level in mice with different reperfusion durations. **C**, **D**, **E**, **F** Two types of FXR agonizts suppressed TLR4 expression in a dose-dependent manner in AML12 cells or mouse primary hepatocytes. **G** The promoter region of the mouse TLR4 gene contained several FXREs. **H**, **J** Transfection of pGL3-mTLR4-prom in the presence or absence of pcDNA3.1-FXR in AML12 cells or mouse primary hepatocytes for 24 h, and the luciferase assay was then performed. **I**, **K** AML12 cells or mouse primary hepatocytes were transfected with FXR overexpression or control plasmids. ChIP analysis was performed with anti-FXR or IgG (*n* = 3 per group). Data are the mean ± SEM, ^∗^*p* < 0.05; ^∗∗^*p* < 0.01; ^∗∗∗^*p* < 0.001 by unpaired Student’s *t* test.

To further investigate the specific mechanism underlying FXR activation and TLR4 suppression, we first detected FXR and TLR4 expression in 20 clinical benign surgical liver samples by Western blotting analysis and found that FXR expression was negatively correlated with TLR4 (Fig. 6A), which was also confirmed in mouse models with different reperfusion times (Fig. 6B). An in vitro study demonstrated that FXR activation inhibited TLR4 expression in a dose-dependent manner, as confirmed by the application of the endogenous ligand CDCA (Fig. 6C, E) and the pharmacological agonist GW4064 (Fig. 6D, F). Subsequently, we assessed whether the FXR response element (FXRE) existed in the promoter region of the TLR4 gene using JASPAR search analysis, and 33 binding sites were detected. Figure 6G shows schematic diagrams of 3 representative FXR binding sites on the TLR4 promoter. To verify whether FXR regulates TLR4 promoter activity, we constructed a luciferase reporter expression plasmid (pGL3-mTLR4-prom) driven by a mouse TLR4 promoter. AML12 cells and mouse primary hepatocytes were transfected with pGL3-mTLR4-prom in the presence or absence of the FXR expression vector. Figure 6H–K show that FXR could directly bind to the FXRE located in the promoter region of TLR4 and inhibit TLR4 expression.

These results suggested that FXR activation induced by Abx treatment could regulate TLR4-mediated MAPK and NF-κB signaling pathways and influence the CCL2–CCR2 axis both in vivo and in vitro. FXR regulated TLR4 expression by repressing the transcriptional activity of the TLR4 gene promoter.

### Hepatic FXR levels are inversely correlated with liver damage

**Fig. 7:**
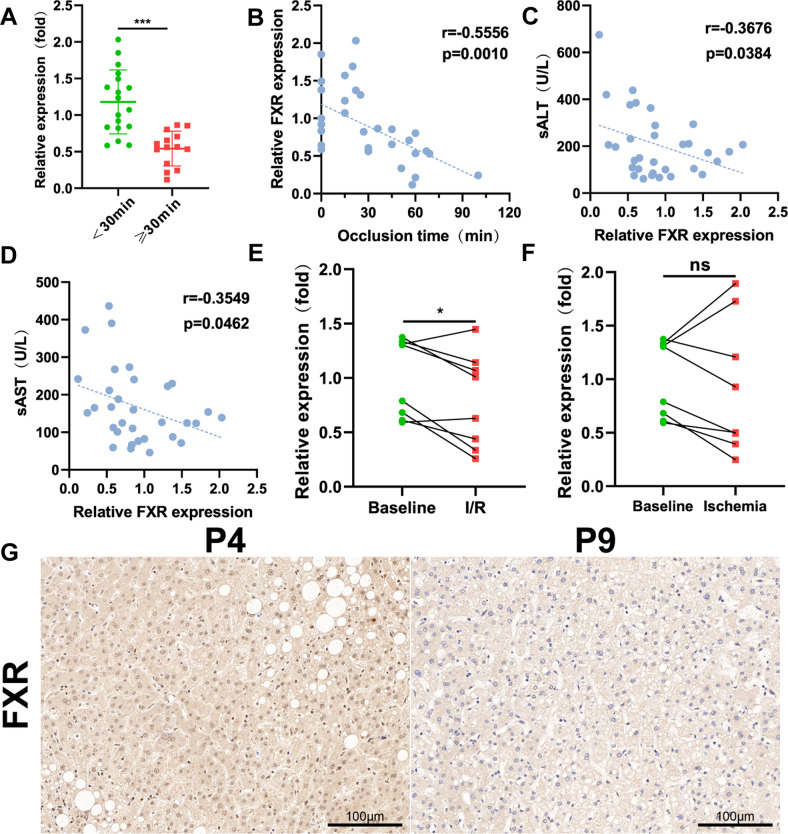
Liver FXR levels are inversely correlated with liver damage. **A** The expression of hepatic FXR at the mRNA level in clinical samples with different occlusion times. Data are the mean ± SEM, ^∗∗^*p* < 0.01. **B** Pearson correlation analysis between relative FXR expression and occlusion times. **C**, **D** Pearson correlation analysis between relative FXR expression and sALT or sAST. **E**, **F** Relative FXR expression in the sham and IRI or ischemia groups in a GEO dataset (GSE12720). **G** Representative FXR staining of clinical liver sections. Scale bars, 100 μm. Schematic illustrating the possible mechanisms of the protective role of Abx pretreatment in hepatic IRI.

**Fig. 8:**
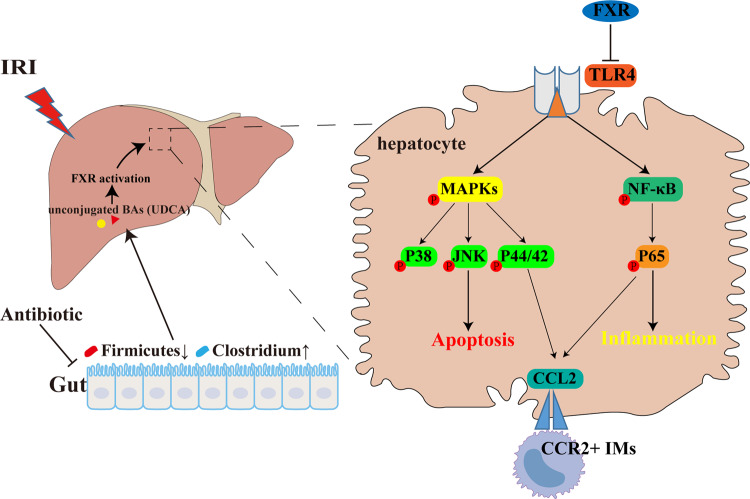
Schematic summary of the present study.

To study the expression levels of FXR in the livers of patients who underwent liver surgery with occlusion of hepatic blood flow, we collected 32 liver specimens from patients undergoing surgical resection of benign liver lesions (Table S2). Routine liver surgery blocks hepatic blood flow for 15 min for operation, followed by reperfusion for 5 min. Therefore, we divided the 32 specimens into two groups with an occlusion time of 30 min. The expression of FXR was obviously decreased with the extension of occlusion time, evidenced by qRT-PCR analysis (Fig. 7A) and Pearson correlation analysis (Fig. 7B). Pearson correlation analysis between relative FXR expression and liver enzymes showed that hepatic FXR levels were inversely correlated with liver damage levels (Fig. 7C, D). We also analyzed FXR expression levels in a Gene Expression Omnibus (GEO) data set (GSE12720) and confirmed that FXR was decreased in response to IRI but not ischemic injury in living donor liver grafts (Fig. 7E, F). The abovementioned findings suggested that the FXR levels in mice and clinical liver occlusion samples were inversely associated with the level of liver damage, indicating a critical role of FXR in hepatic IRI (Fig. 8).

## Discussion

As a common complication in liver resection and transplantation, IRI has been associated with diverse and complex mechanisms, such as activation of TLRs and the innate immune response [21, 33]. Recently, some studies have confirmed the critical role of the gut flora in hepatic IRI [7–9]. However, none of them have explored the relationship between Abx pretreatment and FXR activation in hepatic IRI. We, for the first time, demonstrated that Abx pretreatment could reshape the gut microbiota, alter the liver BA composition and protect the liver from IRI-induced injury by activating FXR. Moreover, Abx pretreatment protected the liver by downregulating TLR4 and suppressing downstream MAPK/NF-κB signaling and the CCL2–CCR2 axis, thereby alleviating liver injury and reducing inflammation. Abx pretreatment mediated the protection from hepatic IRI in an FXR-dependent manner, as confirmed by FXR activation after Abx treatment, and liver-specific FXR inhibition abolished the Abx-mediated protective role of hepatic IRI. Mechanistically, activation of FXR decreased the transcription of TLR4 by binding to an FXRE harbored in the TLR4 promoter.

The gut flora is critical in the process of IRI. Specific bacteria, such as *Lactobacillus acidophilus*, alleviate renal IRI by improving intestinal microbial distribution and anti-inflammatory responses [34]. Bifidobacterium bifidum PRL2010 can inhibit the adhesion of pathogens and enhance the host innate immunity response, thereby attenuating intestinal IRI [35]. The gut microbiota can also function as an environmental factor. Beneficial outcomes have been reported after depletion of gut microbiota by oral application of broad-spectrum antibiotics in different IRI models. Gut flora-depleted mice are protected from renal IRI by reducing mature F4/80+ kidney resident macrophages and bone marrow monocytes [36]. Depletion of gut commensal bacteria in mesenteric IRI mice decreases B cells, Igs, and TLR expression and suppresses the complement system [37]. Meanwhile, the gut microbiome is also important in BA metabolism. BAs are initially synthesized in hepatocytes and secreted into the duodenum with ingestion of food. The majority of the BAs secreted from the liver undergo enterohepatic circulation, while some of the BAs escape and pass into the colon, where they are subjected to bacterial metabolism [38]. By using 16S rDNA sequencing and UHPLC–MS/MS, we found that Abx pretreatment inhibited Firmicutes at the phylum level, leading to the accumulation of unconjugated BAs, and upregulated Clostridium at the genus level, increasing UDCA levels. We confirmed the activation of FXR after depletion of gut flora, which was consistent with previous research [39].

TLR4 signaling in both parenchymal cells and NPCs is fundamental in the development of hepatic IRI [40, 41]. TLR4 greatly contributes to the activation of downstream MAPK and NF-κB pathways. MAPKs, including ERK, p38, and JNK, regulate multiple genes involved in cell apoptosis, proliferation, and growth [42, 43]. NF-κB is a well-recognized pathway in regulating inflammatory responses. Phosphorylation of the IκB kinase (IKK) complex triggers inducible degradation of IκBα, contributing to canonical activation of NF-κB. In renal IRI, TLR4/MAPK/NF-κB pathways are suppressed by the administration of Maresin 1, leading to mitigated inflammation and oxidative stress [44]. In hepatic IRI, TLR4/NF-κB pathway-mediated inflammation is suppressed with ASC deficiency, leading to attenuated injury [45]. In the current study, we found that inhibition of TLR4 by FXR activation suppressed downstream MAPK and NF-κB pathways, contributing to decreased apoptotic cells and inflammatory responses.

Inflammatory cell infiltration is one of the characteristics of liver IRI, which is mediated by chemokines and cytokines. During hepatic inflammation, many CCL2 molecules are released from Kupffer cells, injured hepatocytes, and activated hepatic stellate cells (HSCs), leading to hepatic accumulation of CCR2-positive monocytes initially from bone marrow [46, 47]. CCR2-positive monocytes express high levels of Ly6C, a surface protein allowing hepatic accumulation [30]. In the current study, we found that depletion of gut flora significantly decreased the expression of CCL2 and reduced the infiltration of CCR2-positive monocytes, and this process was mediated by FXR activation, as evidenced by liver-specific FXR knockdown. MAPK and NF-κB signaling are associated with the expression of CCL2 [32, 48]. In our current work, we showed that the expression of CCL2 was consistent with MAPK and NF-κB signaling, indicating that these signaling pathways participated in the FXR-mediated regulation of CCL2.

FXR was initially considered a nuclear receptor for farnesol metabolites, and subsequent studies have revealed its multiple functions in various diseases. Abnormally high levels of BAs are one of the pathogenic factors contributing to cholestatic liver diseases, and FXR regulates the levels of hepatic BAs, thus being viewed as a therapeutic target for primary biliary cholangitis [49, 50]. In colon cancer, the expression of FXR is inversely correlated with clinical outcomes [51], and in vitro studies have also demonstrated the downregulation of FXR in colon cancer cells [52]. Mechanistically, FXR suppresses the expression of miR-135A1, resulting in upregulation of cyclin D2 (CCNG2) [53]. However, FXR functions in liver IRI have rarely been studied, despite its protective role in hepatic IRI [12]. In the current work, we confirmed that FXR activation could suppress the expression of TLR4 by directly binding to the FXRE harbored in the TLR4 promoter firstly, thereby regulating downstream MAPK and NF-κB pathways and affecting cell apoptosis and the inflammatory response. We also confirmed the protective role of FXR in mice and clinical samples, and there was an obvious negative relationship between the degree of liver damage and the levels of FXR.

In summary, our work confirmed that depletion of gut flora altered the composition of hepatic BAs, leading to FXR activation, which could directly regulate the transcriptional activity of TLR4 and further alleviate hepatic IRI by decreasing cell apoptosis and reducing the inflammatory response. Collectively, our study suggested that FXR was a promising target in liver injury. Additional studies are needed to explore the functions of FXR in different liver diseases.

## Supplementary information

Reproducibility Checklist

uncropped western blots

supplementary materials

Detailed Attribution of Authorship

Supplemental Material

Supplemental Material

Supplemental Material

Supplemental Material

Supplemental Material

Supplemental Material

Supplemental Material

Supplemental Material

Supplemental Material

Supplemental Material

Supplemental Material

Supplemental Material

Supplemental Material

Supplemental Material

Supplemental Material

Supplemental Material

Supplemental Material

Supplemental Material

Supplemental Material

Supplemental Material

Supplemental Material

Supplemental Material

Supplemental Material

Supplemental Material

Supplemental Material

Supplemental Material

Supplemental Material

Supplemental Material

Supplemental Material

Supplemental Material

Supplemental Material

Supplemental Material

Supplemental Material

Supplemental Material

Supplemental Material

Supplemental Material

Supplemental Material

Supplemental Material

Supplemental Material

Supplemental Material

Supplemental Material

Supplemental Material

Supplemental Material

Supplemental Material

Supplemental Material

Supplemental Material

Supplemental Material

Supplemental Material

Supplemental Material

Supplemental Material

Supplemental Material

Supplemental Material

Supplemental Material

Supplemental Material

Supplemental Material

Supplemental Material

Supplemental Material

Supplemental Material

Supplemental Material

Supplemental Material

Supplemental Material

Supplemental Material

Supplemental Material

Supplemental Material

Supplemental Material

Supplemental Material

Supplemental Material

Supplemental Material

Supplemental Material

Supplemental Material

Supplemental Material

Supplemental Material

Supplemental Material

Supplemental Material

Supplemental Material

Supplemental Material

Supplemental Material

Supplemental Material

Supplemental Material

Supplemental Material

Supplemental Material

Supplemental Material

Supplemental Material

Supplemental Material

Supplemental Material

Supplemental Material

Supplemental Material

Supplemental Material

Supplemental Material

Supplemental Material

Supplemental Material

Supplemental Material

Supplemental Material

Supplemental Material

Supplemental Material

Supplemental Material

Supplemental Material

Supplemental Material

Supplemental Material

Supplemental Material

Supplemental Material

Supplemental Material

Supplemental Material

Supplemental Material

Supplemental Material

Supplemental Material

Supplemental Material

Supplemental Material

Supplemental Material

Supplemental Material

Supplemental Material

Supplemental Material

Supplemental Material

Supplemental Material

Supplemental Material

Supplemental Material

Supplemental Material

Supplemental Material

Supplemental Material

Supplemental Material

Supplemental Material

Supplemental Material

Supplemental Material

Supplemental Material

Supplemental Material

Supplemental Material

Supplemental Material

Supplemental Material

Supplemental Material

Supplemental Material

Supplemental Material

Supplemental Material

Supplemental Material

Supplemental Material

Supplemental Material

Supplemental Material

Supplemental Material

Supplemental Material

Supplemental Material

Supplemental Material

Supplemental Material

Supplemental Material

Supplemental Material

Supplemental Material

Supplemental Material

Supplemental Material

Supplemental Material

Supplemental Material

Supplemental Material

Supplemental Material

Supplemental Material

Supplemental Material

Supplemental Material

Supplemental Material

Supplemental Material

Supplemental Material

Supplemental Material

Supplemental Material

Supplemental Material

Supplemental Material

Supplemental Material

Supplemental Material

Supplemental Material

Supplemental Material

Supplemental Material

Supplemental Material

Supplemental Material

Supplemental Material

Antibiotic pretreatment attenuates liver ischemia–reperfusion injury by Farnesoid X receptor activation

## Data Availability

The data that support the findings of this study are available from the corresponding author upon reasonable request.
