# Supplementary figures and images for: Antibiotic pretreatment attenuates liver ischemia–reperfusion injury by Farnesoid X receptor activation (part 1 of 2)
Source: Cell Death Dis. 2022 May 21;13(5):484. doi: 10.1038/s41419-022-04955-x (PMC9124217; doi:10.1038/s41419-022-04955-x)

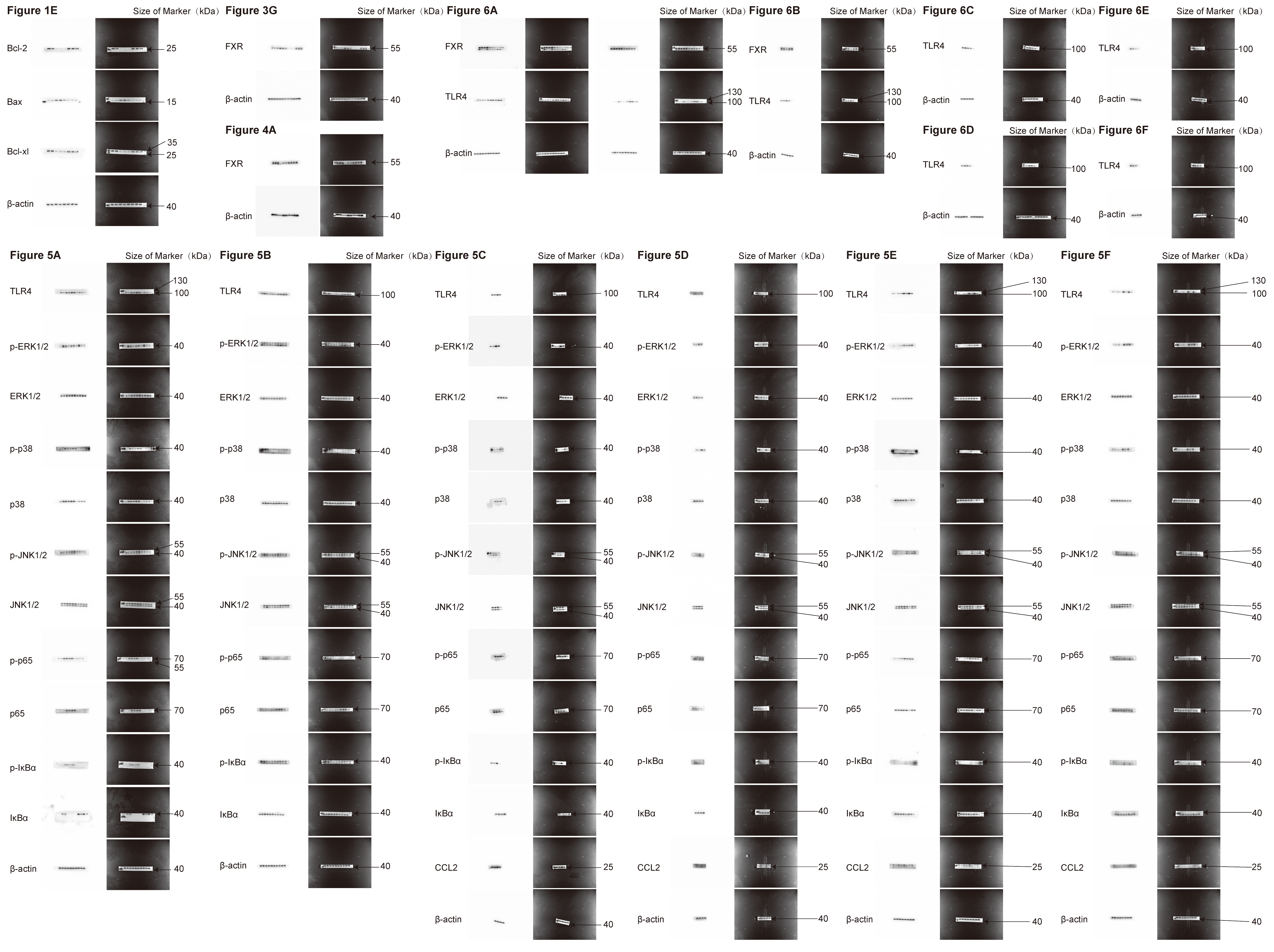

Supplement: Supplementary file 2 — uncropped western blots [file 41419_2022_4955_MOESM2_ESM.png]

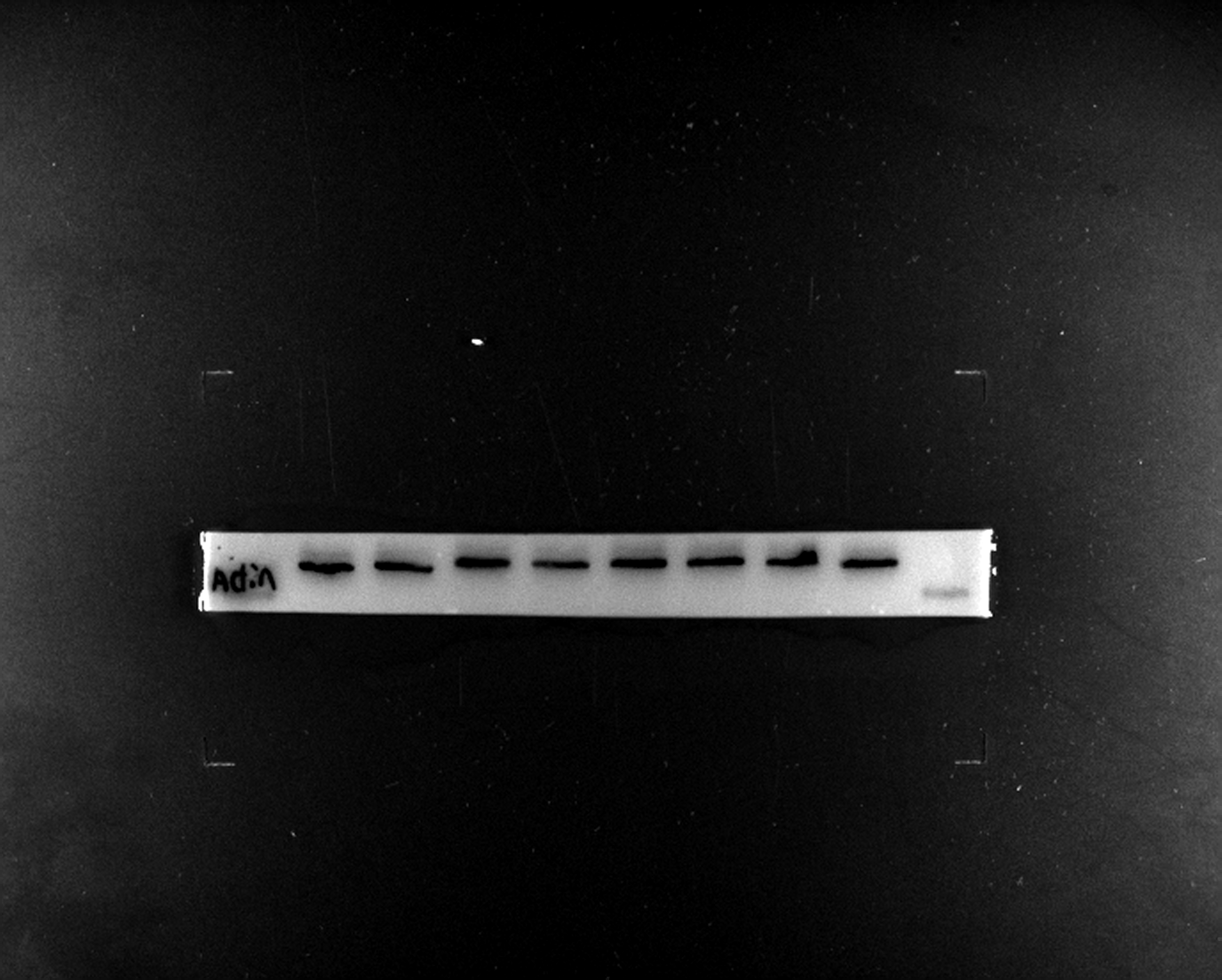

Supplement: Supplementary file 5 — Supplemental Material [file 41419_2022_4955_MOESM5_ESM.tif]

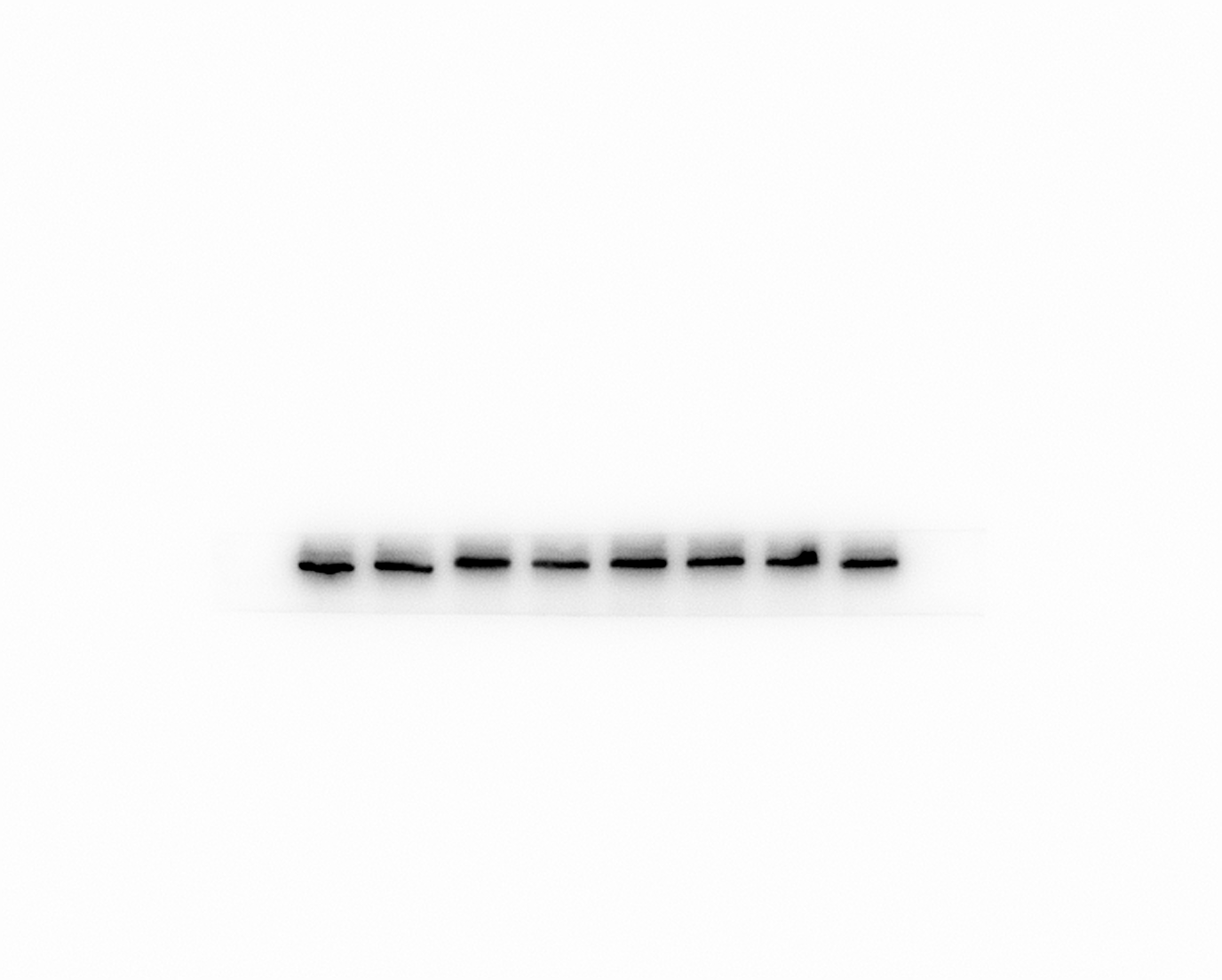

Supplement: Supplementary file 6 — Supplemental Material [file 41419_2022_4955_MOESM6_ESM.tif]

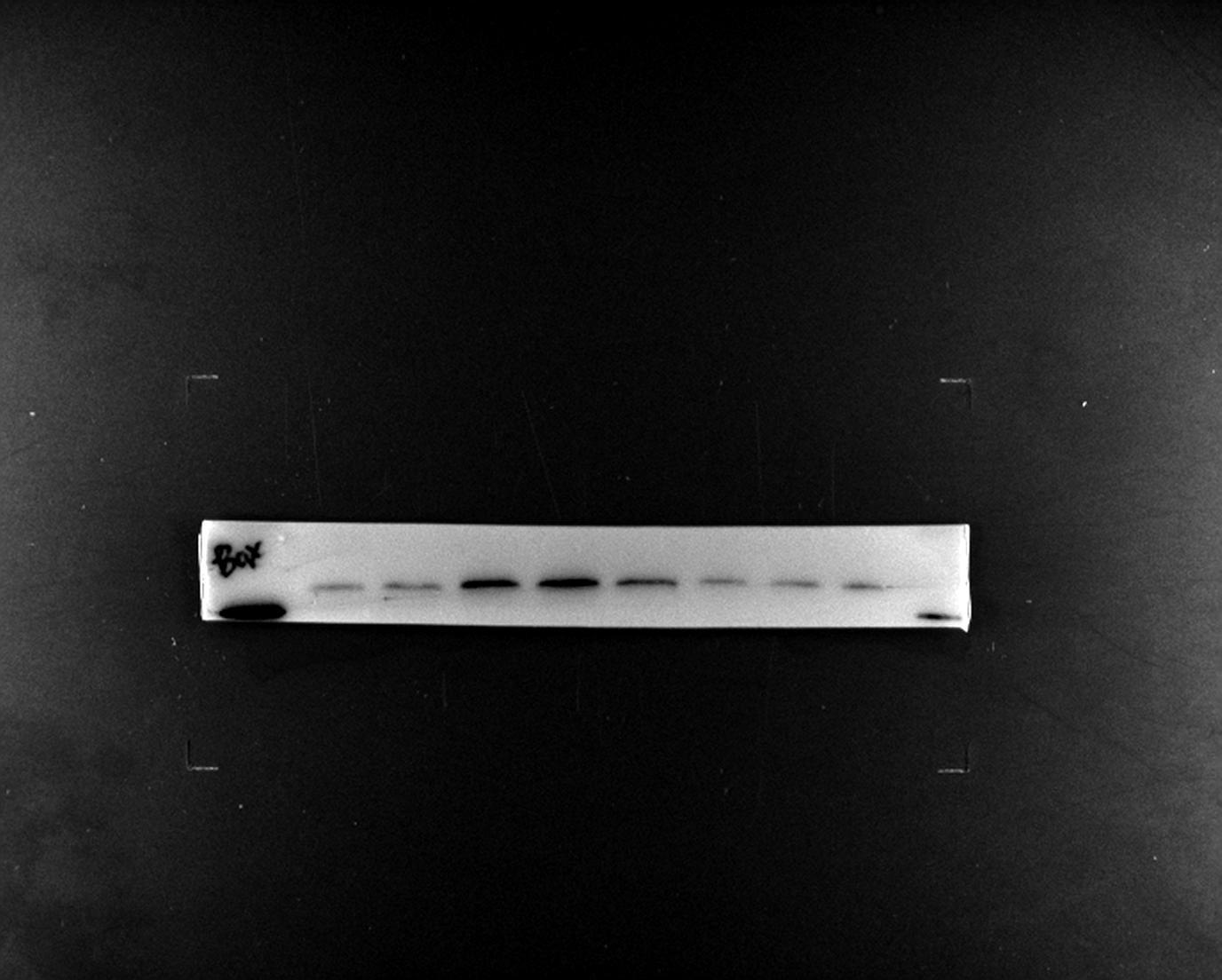

Supplement: Supplementary file 7 — Supplemental Material [file 41419_2022_4955_MOESM7_ESM.tif]

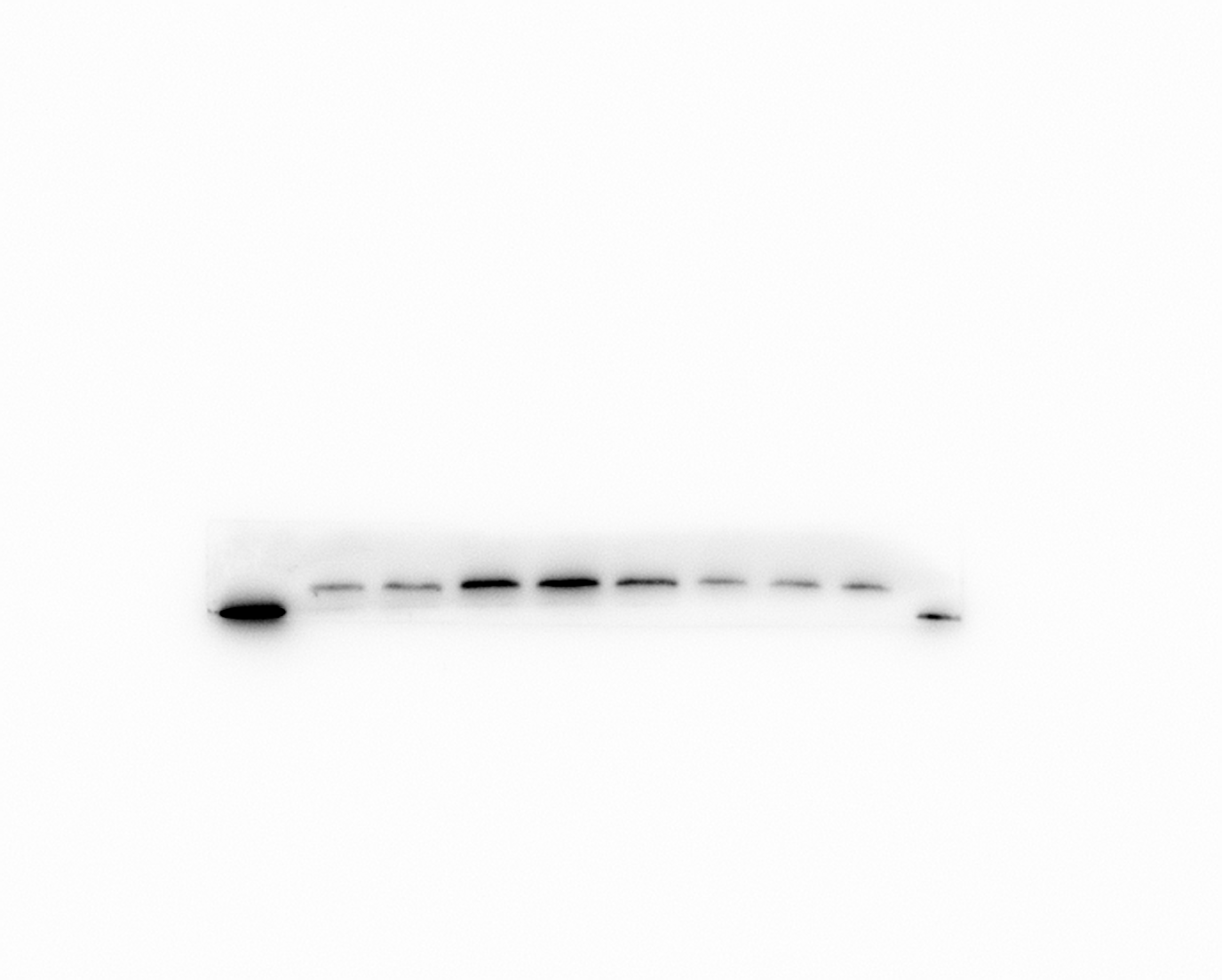

Supplement: Supplementary file 8 — Supplemental Material [file 41419_2022_4955_MOESM8_ESM.tif]

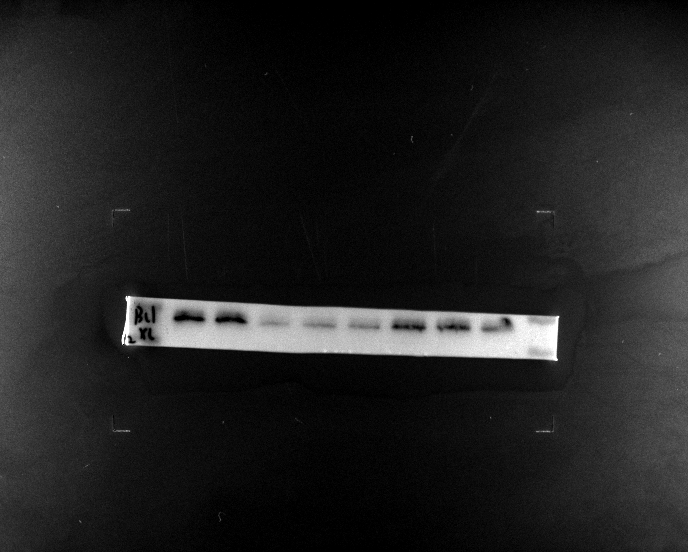

Supplement: Supplementary file 9 — Supplemental Material [file 41419_2022_4955_MOESM9_ESM.tif]

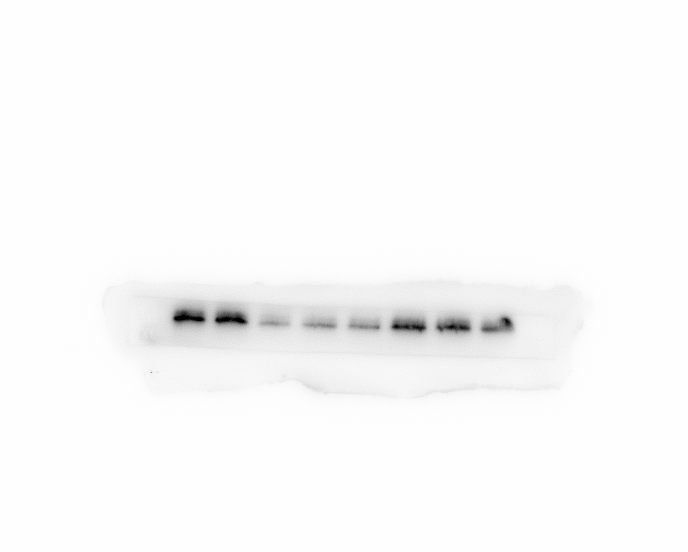

Supplement: Supplementary file 10 — Supplemental Material [file 41419_2022_4955_MOESM10_ESM.tif]

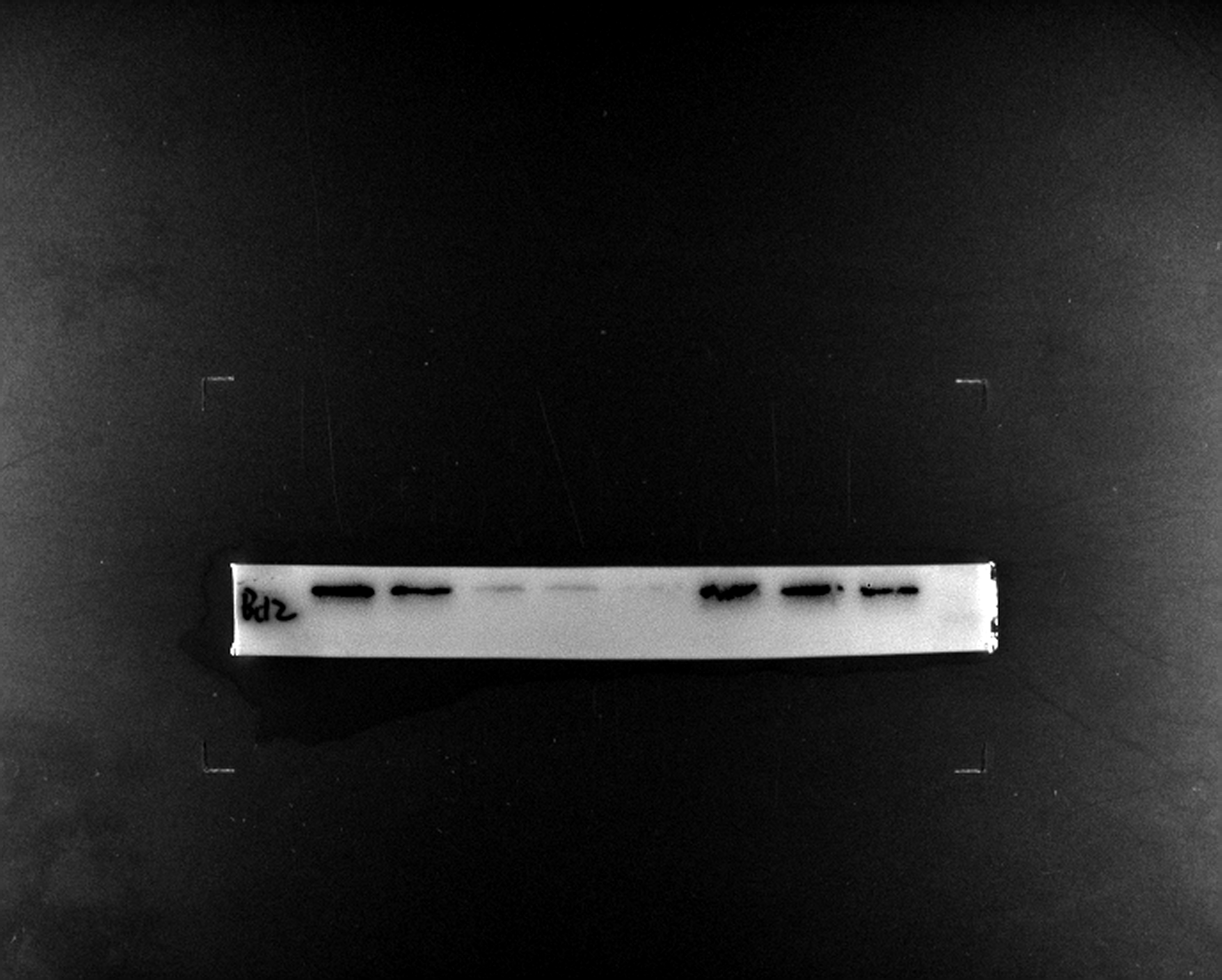

Supplement: Supplementary file 11 — Supplemental Material [file 41419_2022_4955_MOESM11_ESM.tif]

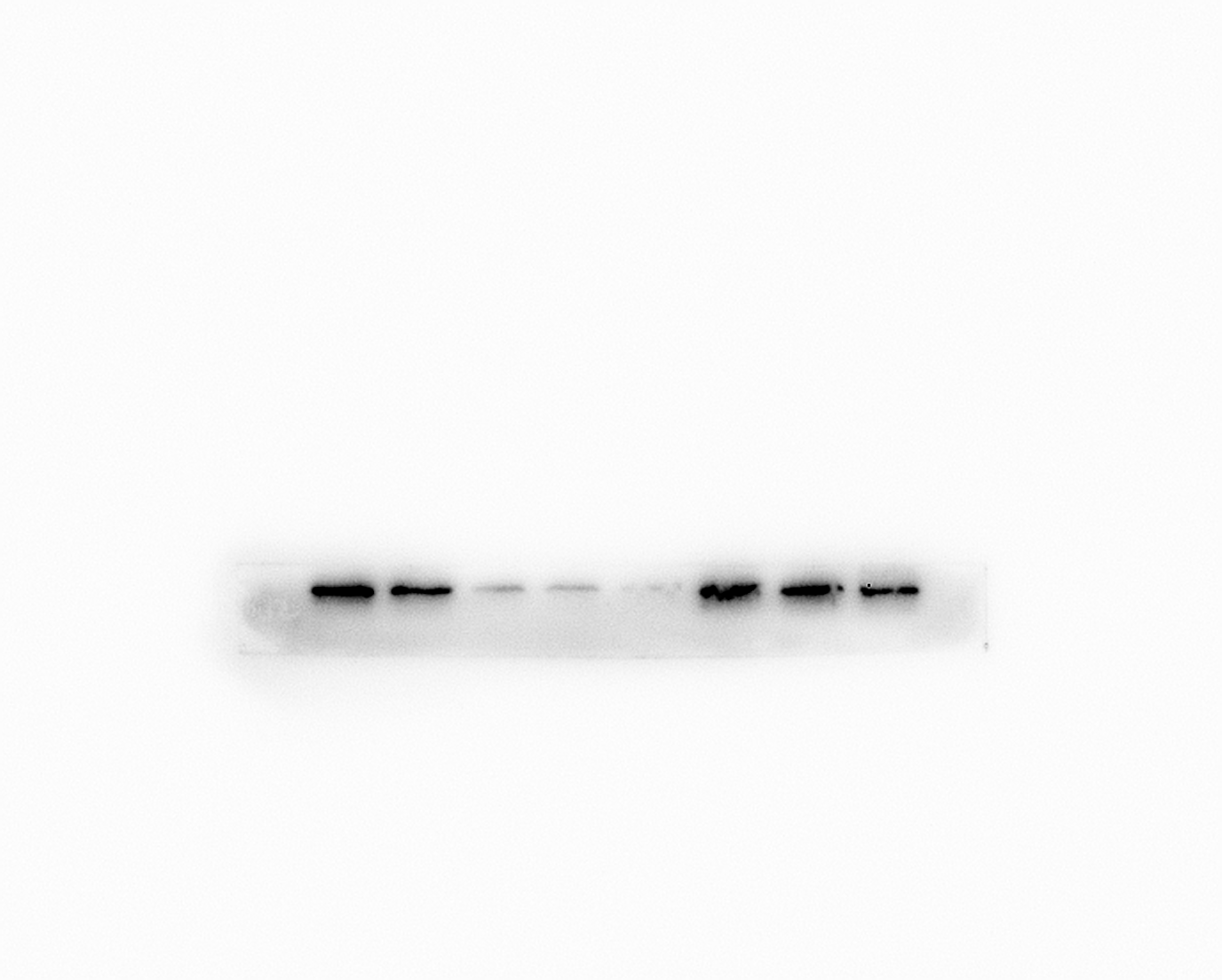

Supplement: Supplementary file 12 — Supplemental Material [file 41419_2022_4955_MOESM12_ESM.tif]

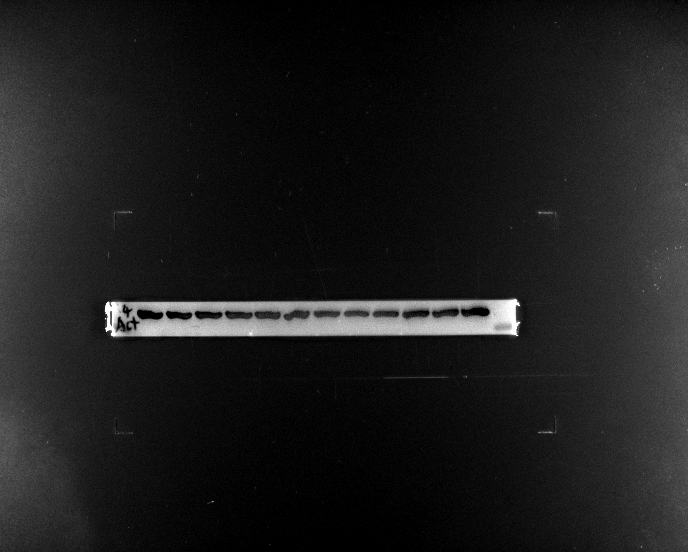

Supplement: Supplementary file 13 — Supplemental Material [file 41419_2022_4955_MOESM13_ESM.tif]

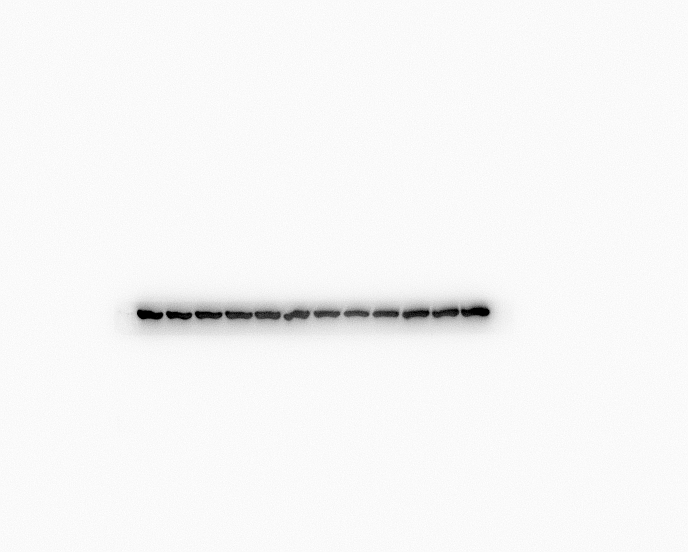

Supplement: Supplementary file 14 — Supplemental Material [file 41419_2022_4955_MOESM14_ESM.tif]

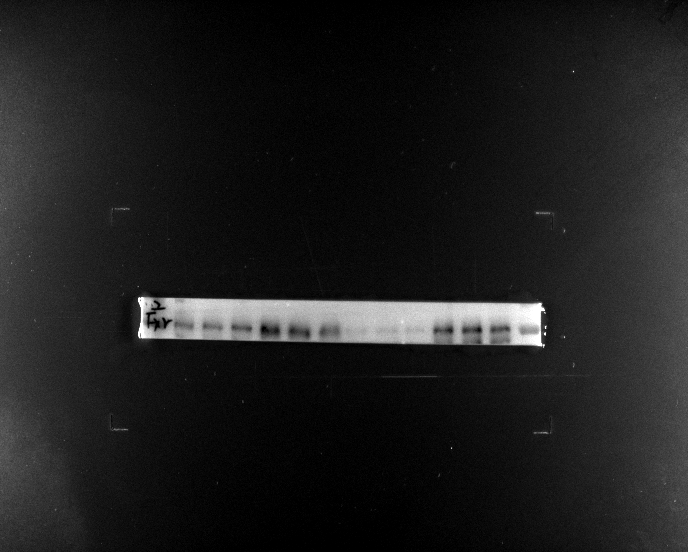

Supplement: Supplementary file 15 — Supplemental Material [file 41419_2022_4955_MOESM15_ESM.tif]

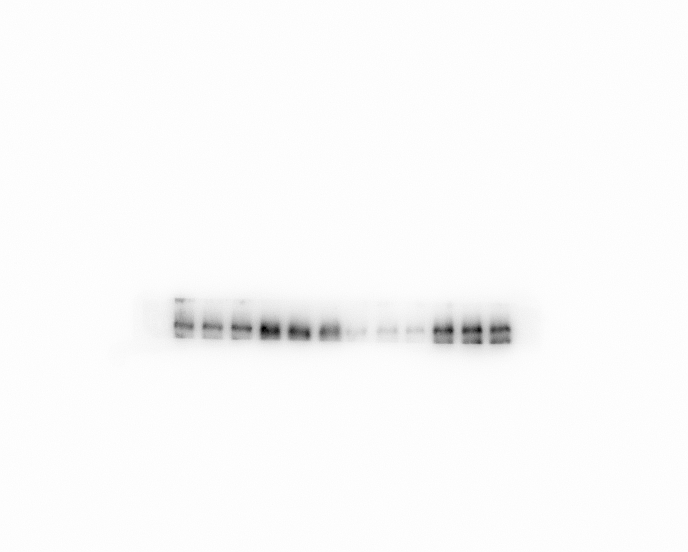

Supplement: Supplementary file 16 — Supplemental Material [file 41419_2022_4955_MOESM16_ESM.tif]

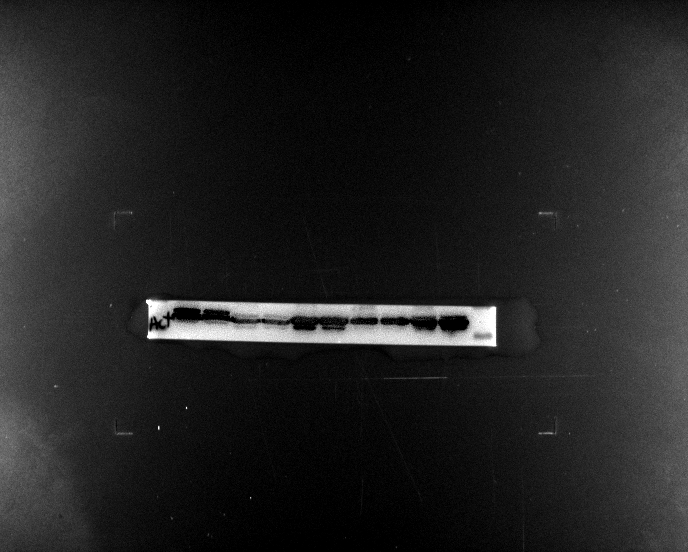

Supplement: Supplementary file 17 — Supplemental Material [file 41419_2022_4955_MOESM17_ESM.tif]

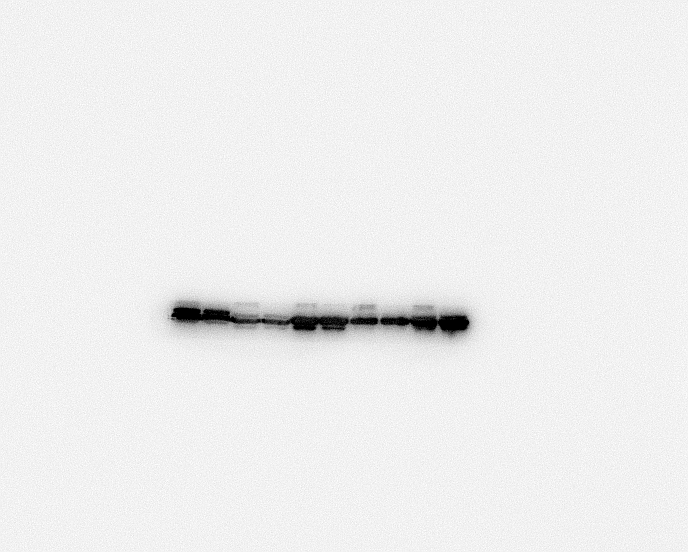

Supplement: Supplementary file 18 — Supplemental Material [file 41419_2022_4955_MOESM18_ESM.tif]

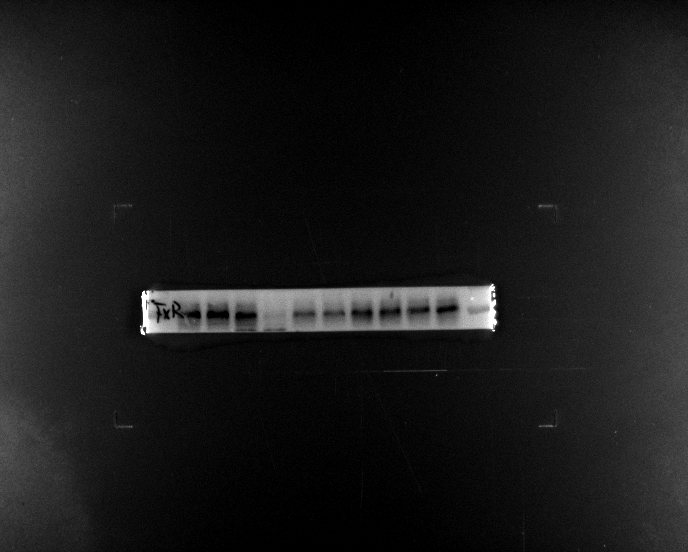

Supplement: Supplementary file 19 — Supplemental Material [file 41419_2022_4955_MOESM19_ESM.tif]

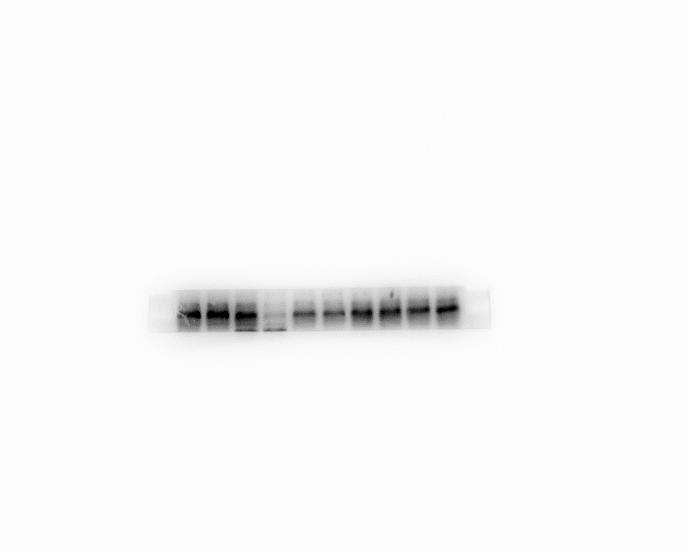

Supplement: Supplementary file 20 — Supplemental Material [file 41419_2022_4955_MOESM20_ESM.tif]

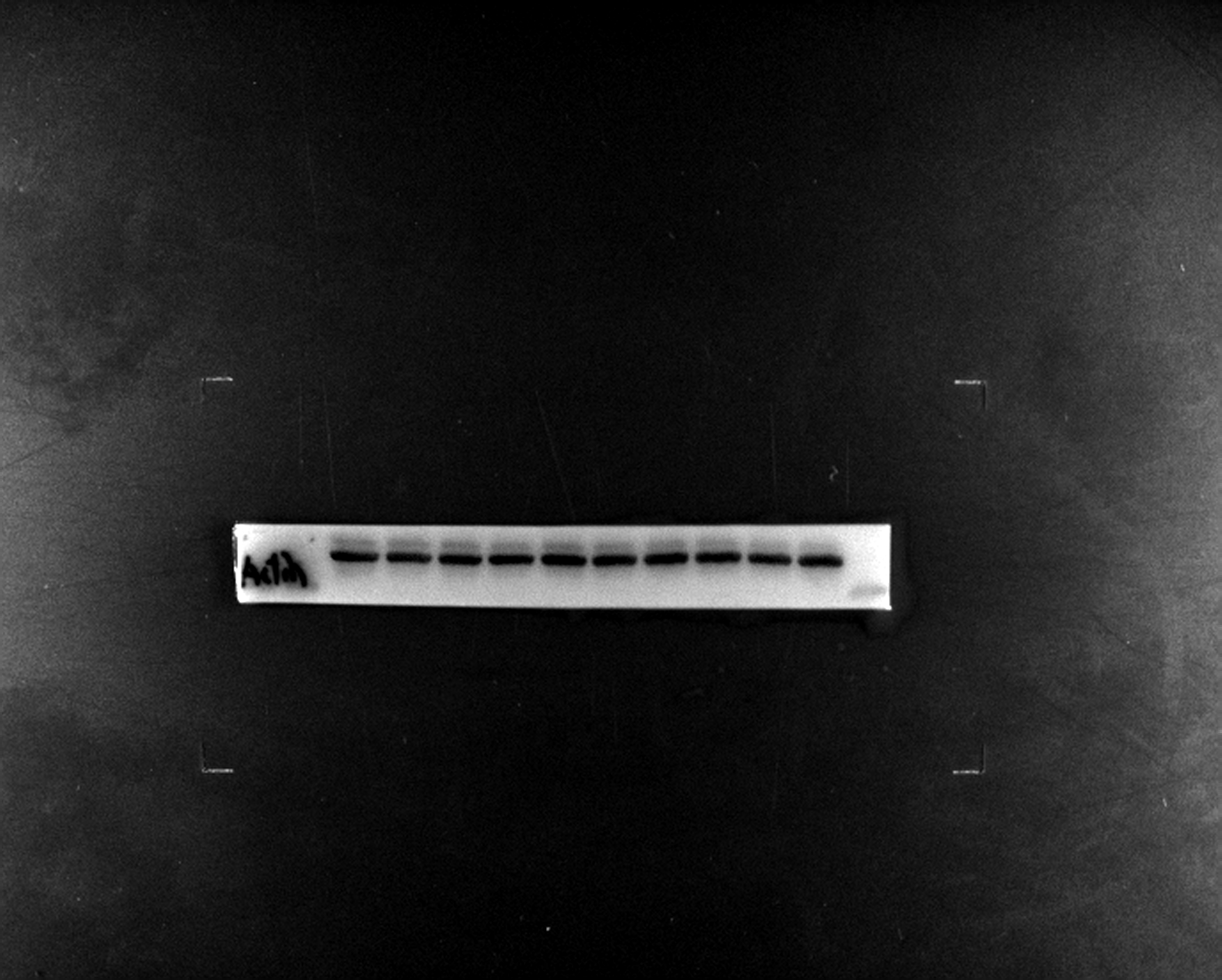

Supplement: Supplementary file 21 — Supplemental Material [file 41419_2022_4955_MOESM21_ESM.tif]

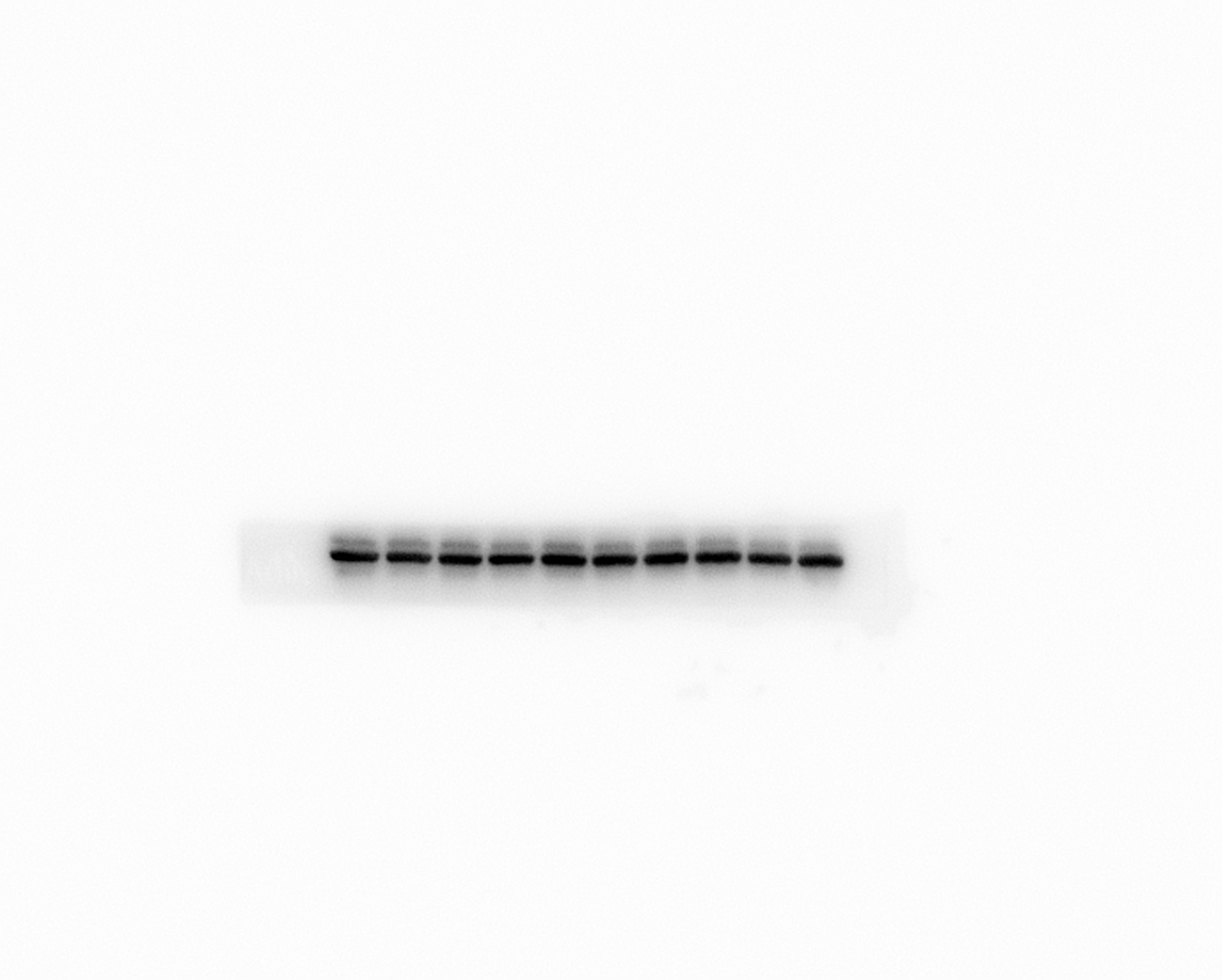

Supplement: Supplementary file 22 — Supplemental Material [file 41419_2022_4955_MOESM22_ESM.tif]

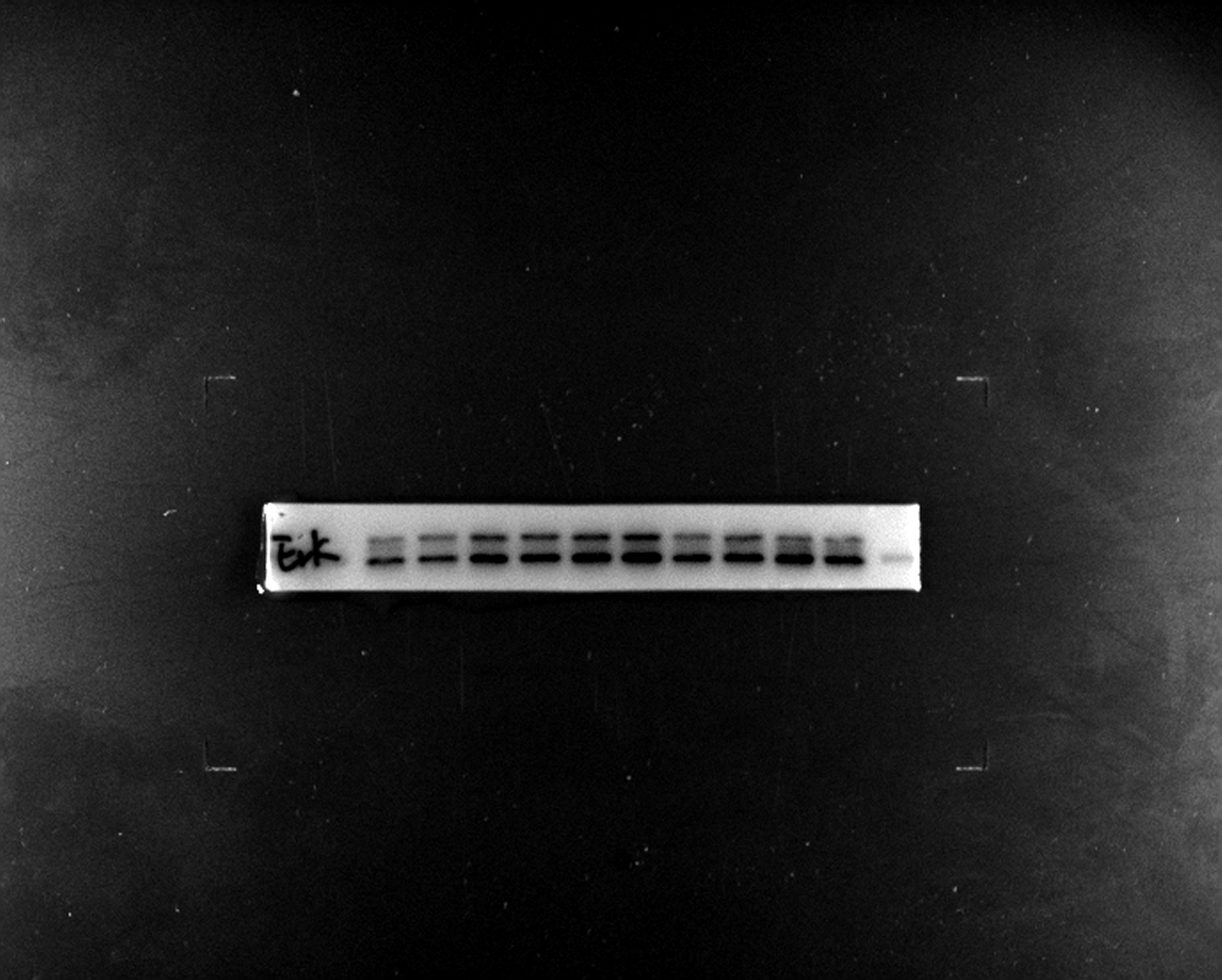

Supplement: Supplementary file 23 — Supplemental Material [file 41419_2022_4955_MOESM23_ESM.tif]

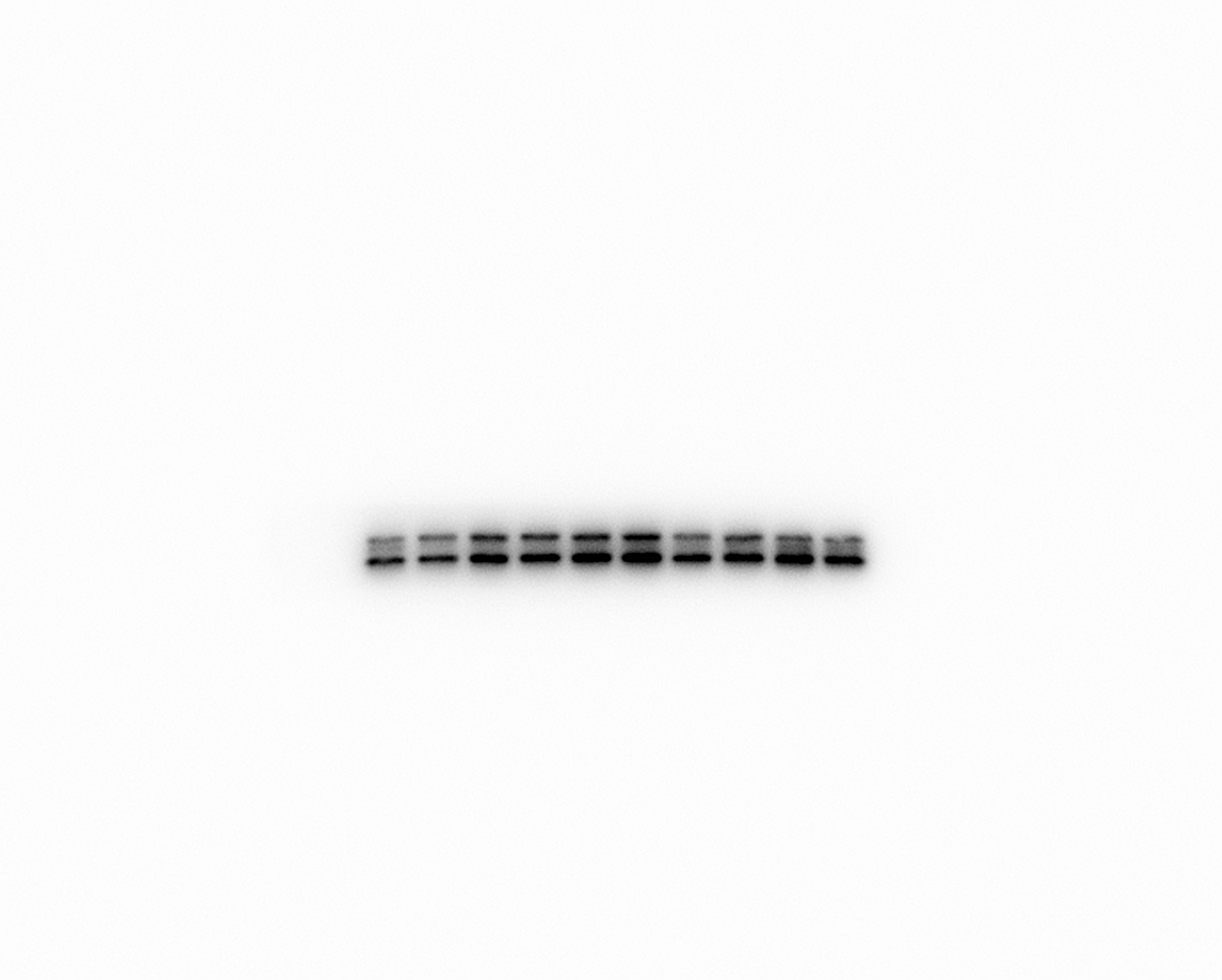

Supplement: Supplementary file 24 — Supplemental Material [file 41419_2022_4955_MOESM24_ESM.tif]

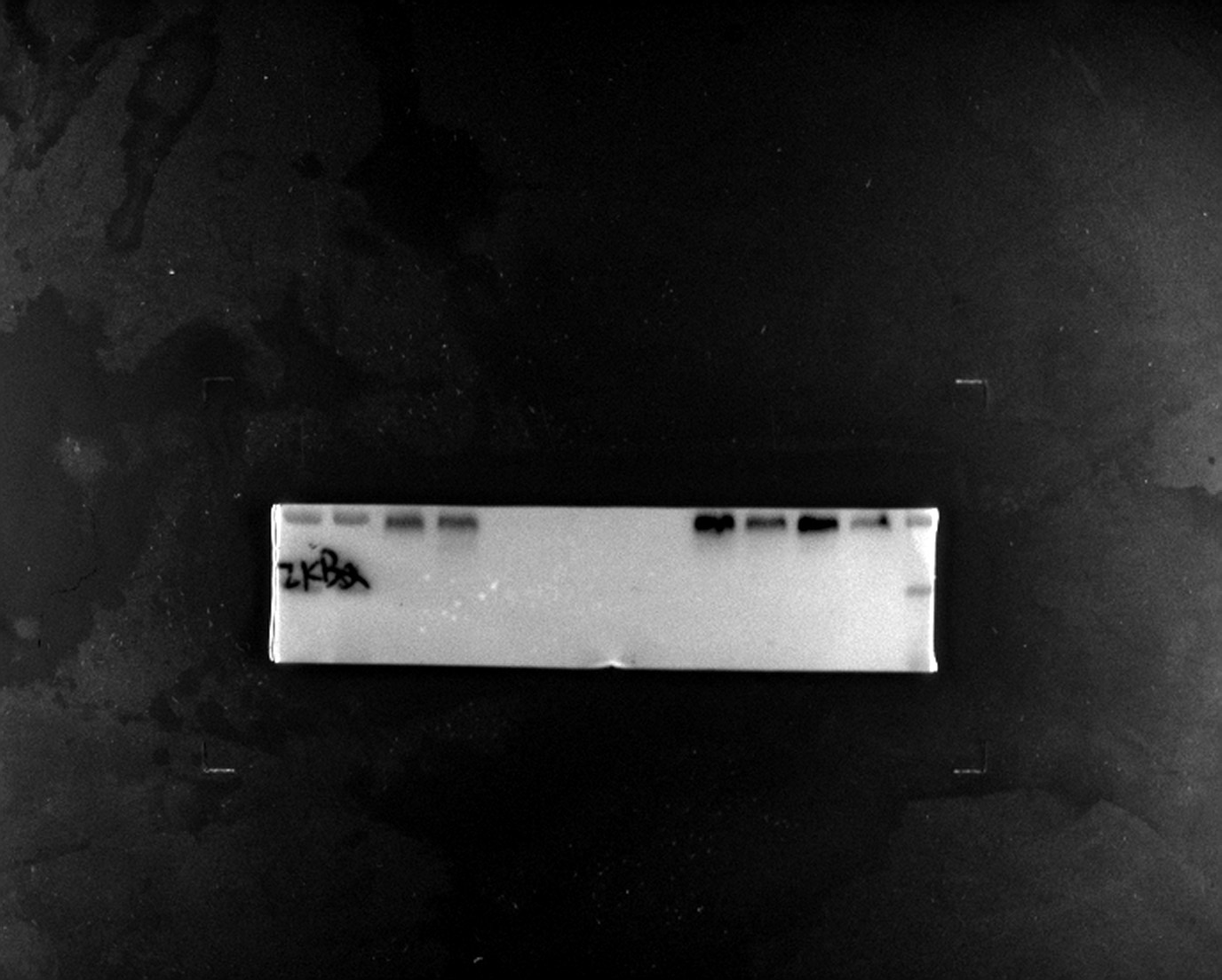

Supplement: Supplementary file 25 — Supplemental Material [file 41419_2022_4955_MOESM25_ESM.tif]

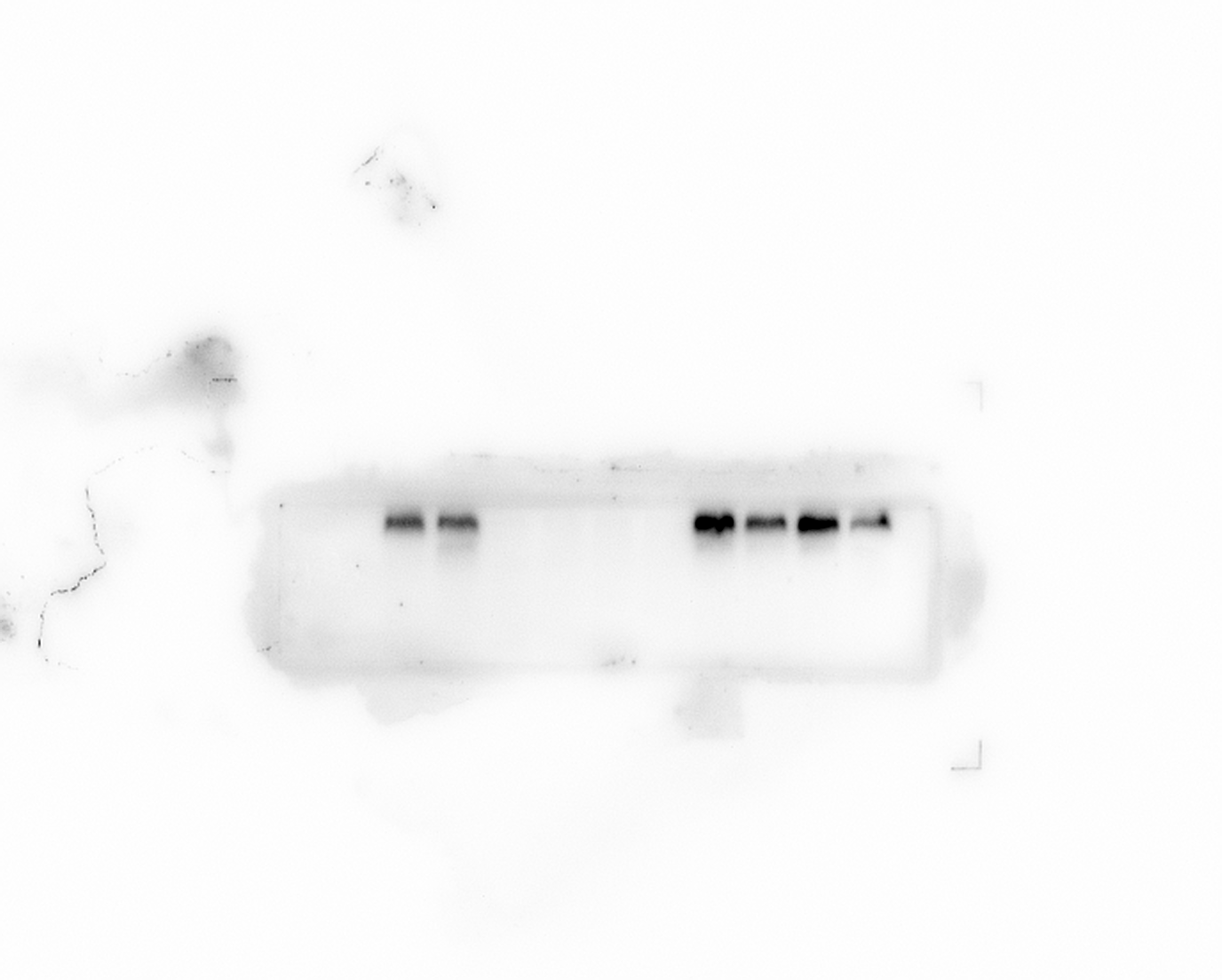

Supplement: Supplementary file 26 — Supplemental Material [file 41419_2022_4955_MOESM26_ESM.tif]

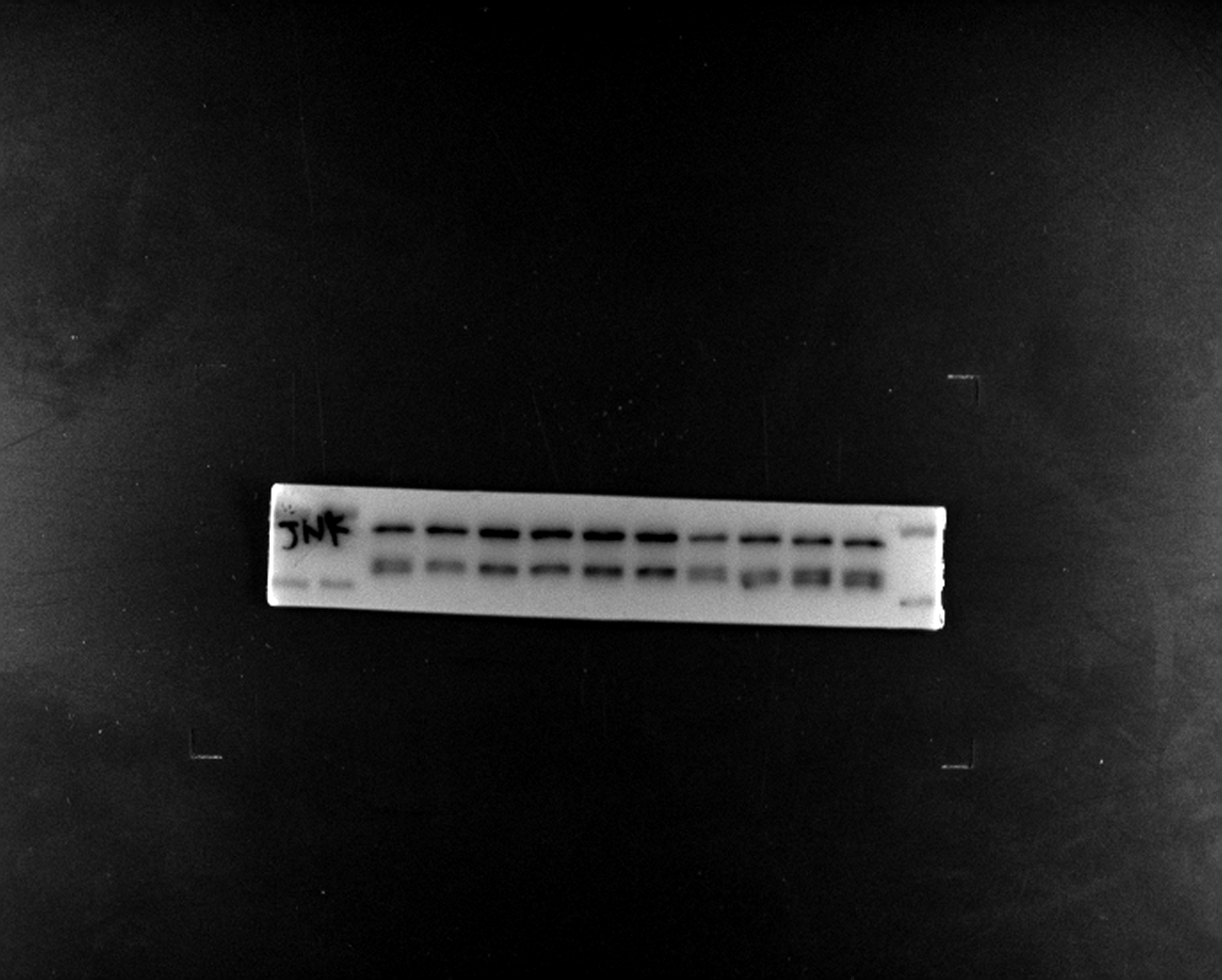

Supplement: Supplementary file 27 — Supplemental Material [file 41419_2022_4955_MOESM27_ESM.tif]

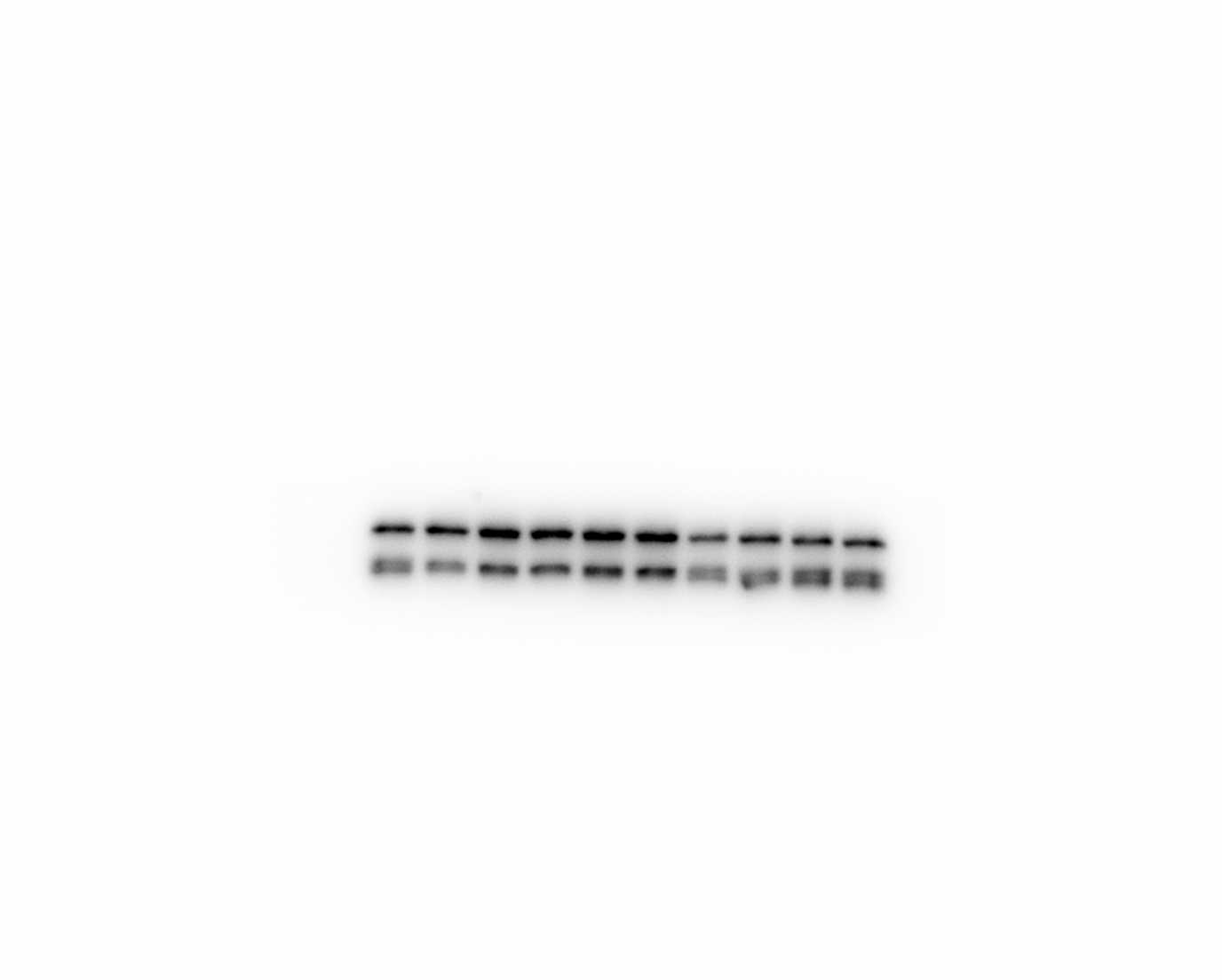

Supplement: Supplementary file 28 — Supplemental Material [file 41419_2022_4955_MOESM28_ESM.tif]

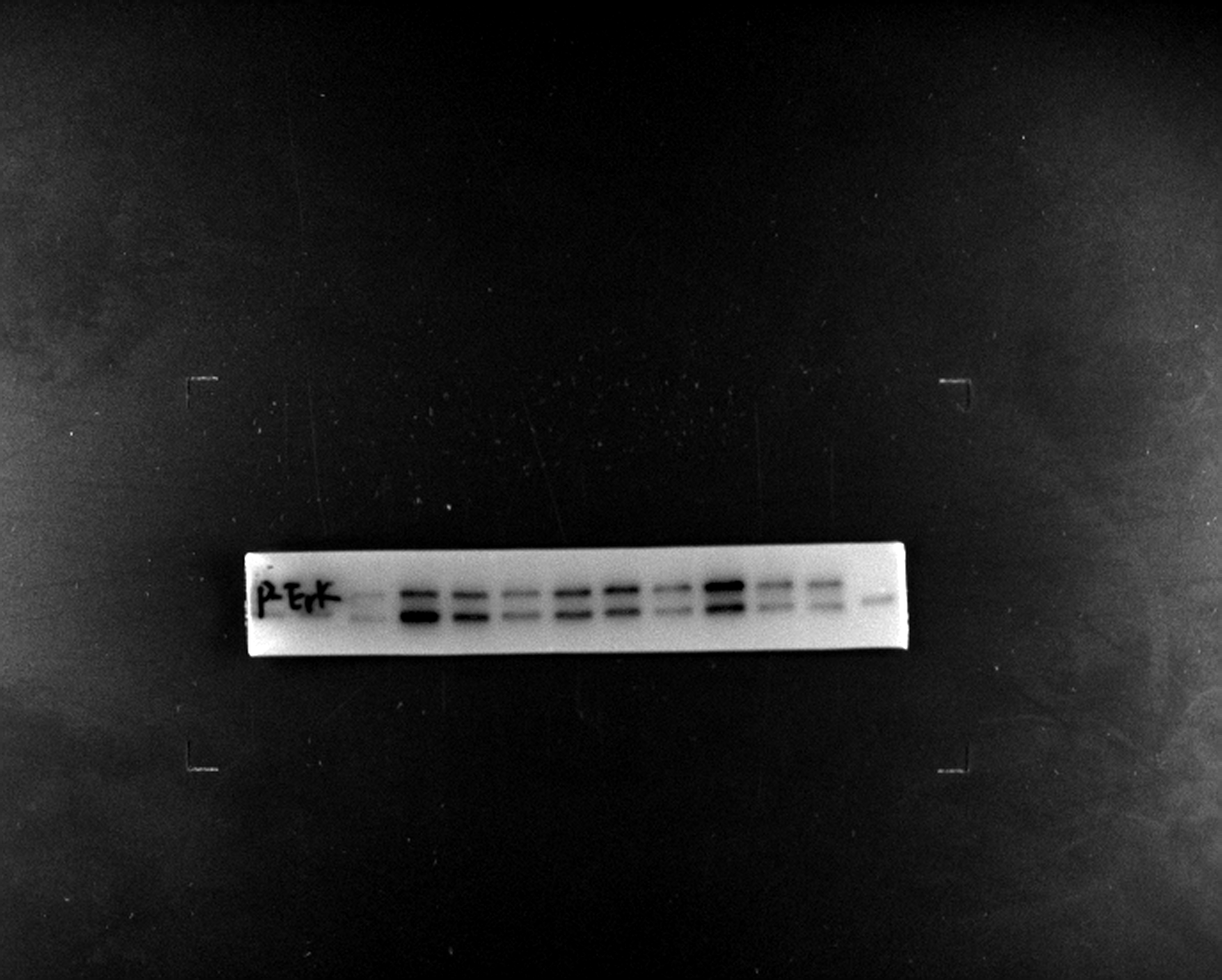

Supplement: Supplementary file 29 — Supplemental Material [file 41419_2022_4955_MOESM29_ESM.tif]

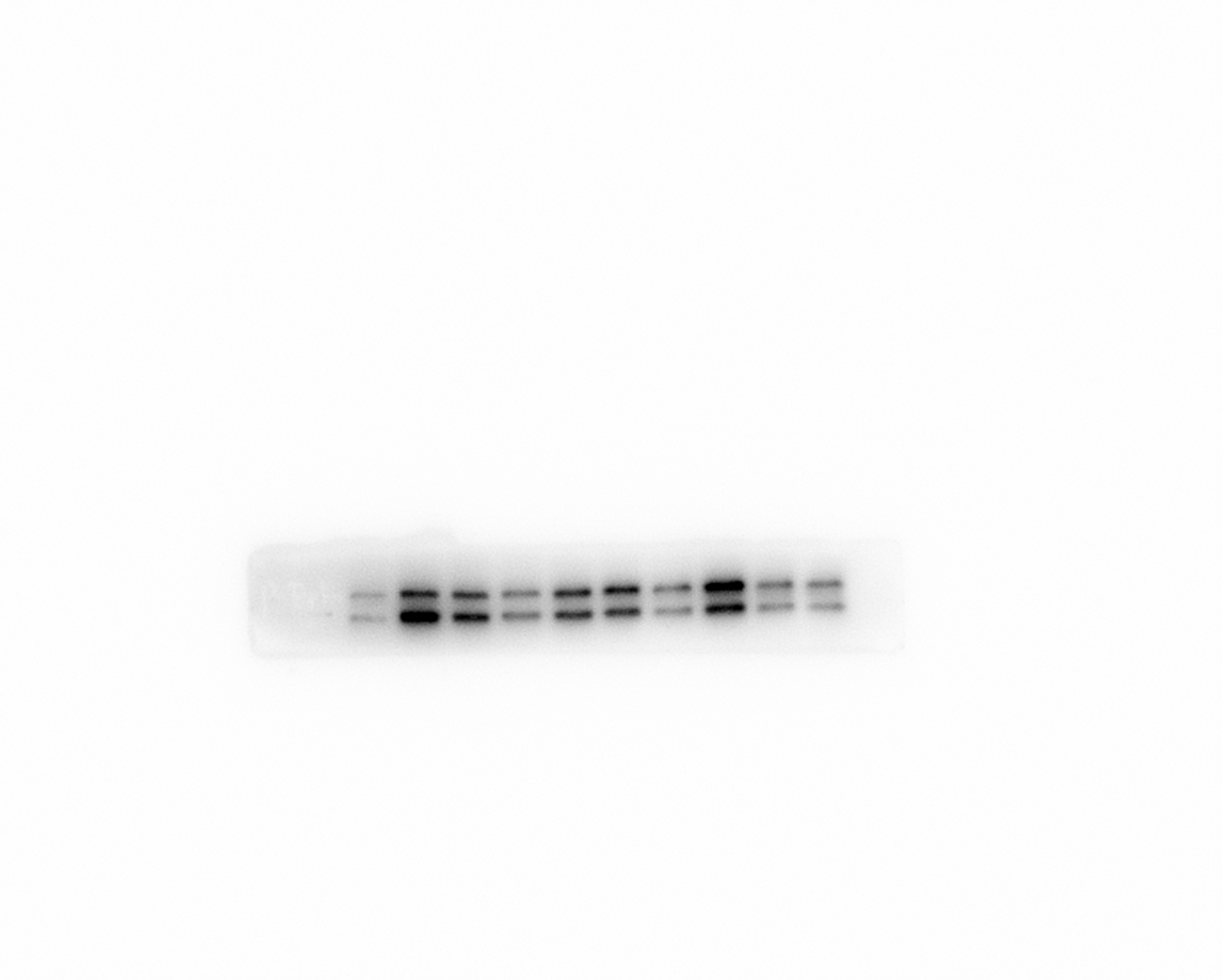

Supplement: Supplementary file 30 — Supplemental Material [file 41419_2022_4955_MOESM30_ESM.tif]

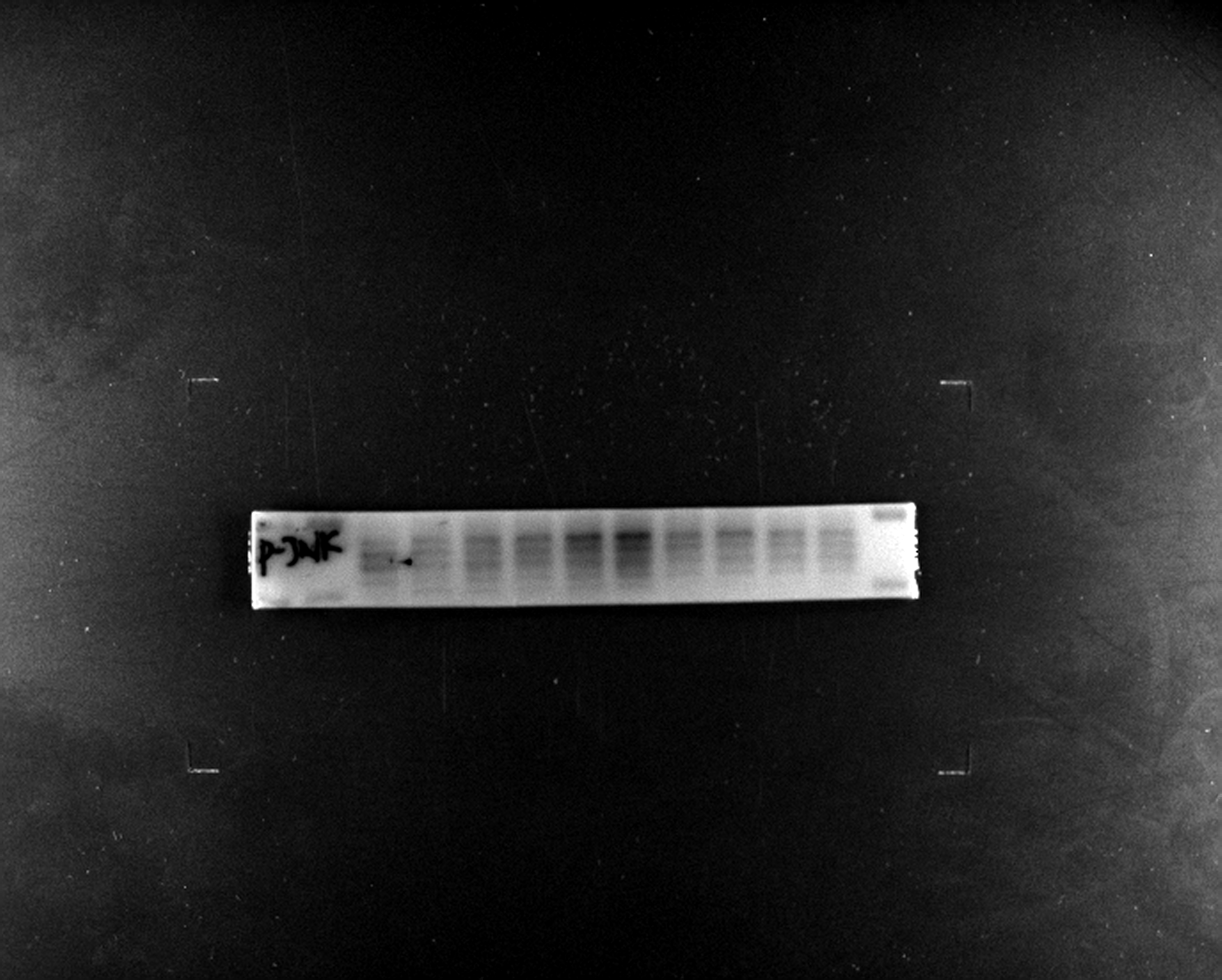

Supplement: Supplementary file 31 — Supplemental Material [file 41419_2022_4955_MOESM31_ESM.tif]

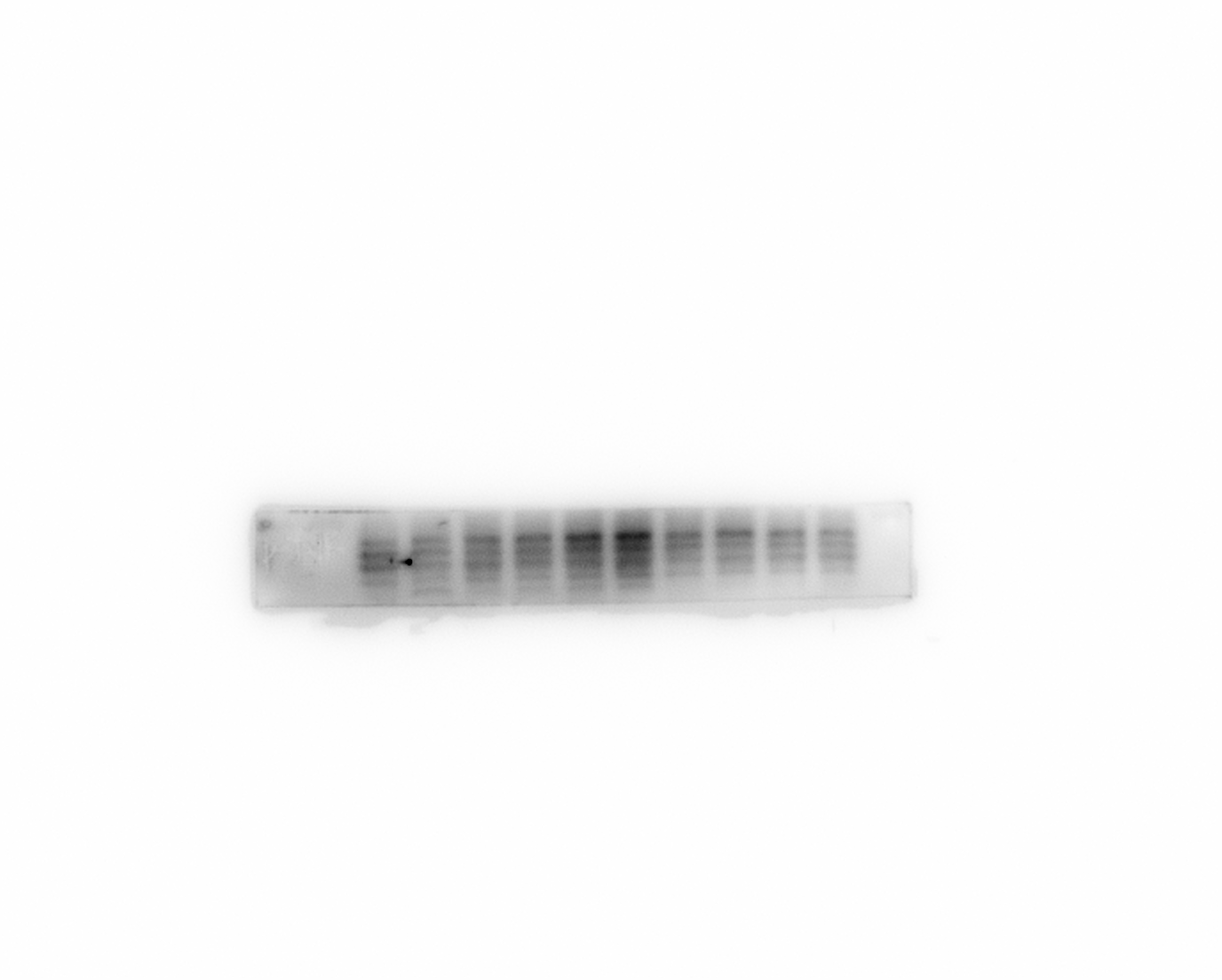

Supplement: Supplementary file 32 — Supplemental Material [file 41419_2022_4955_MOESM32_ESM.tif]

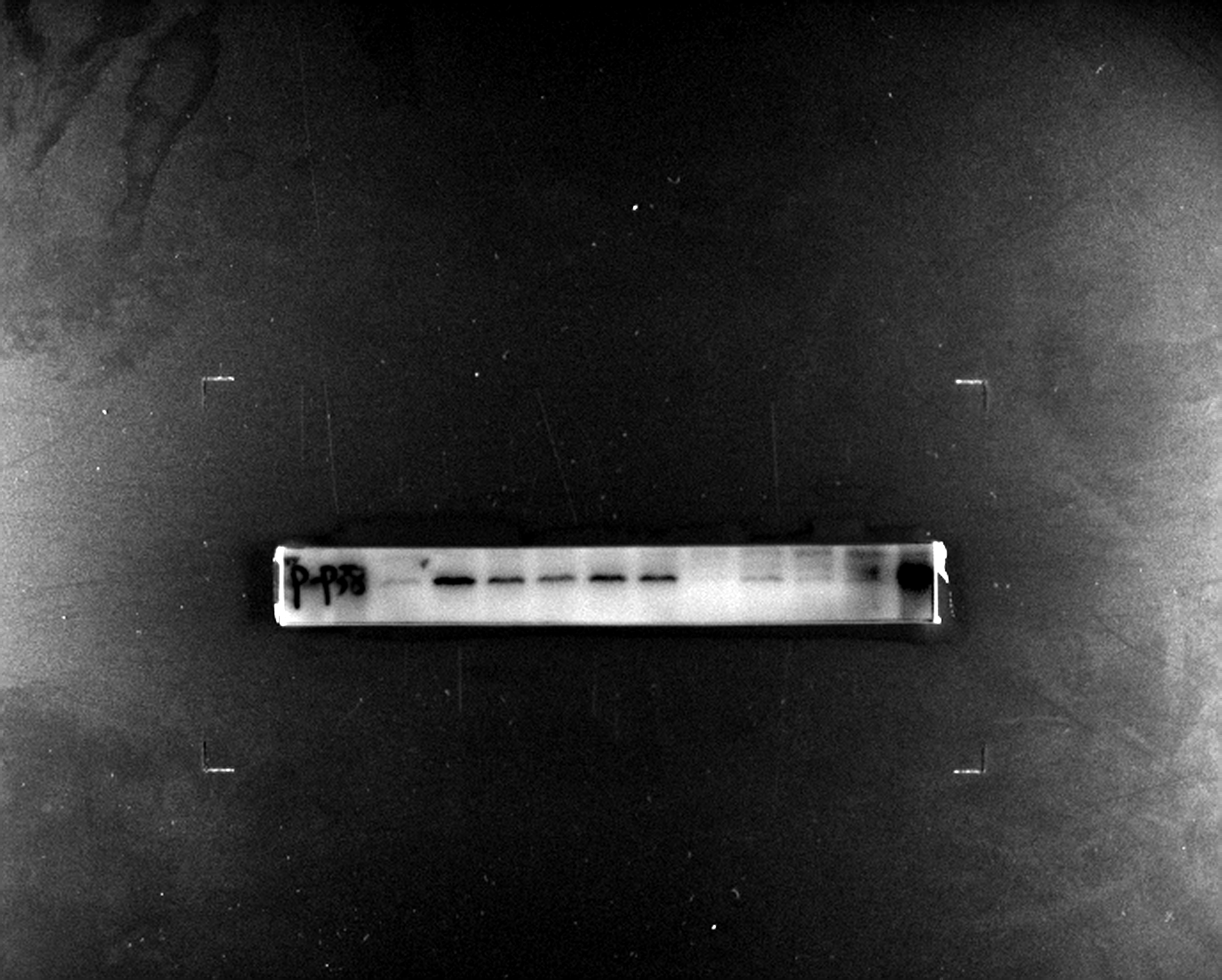

Supplement: Supplementary file 33 — Supplemental Material [file 41419_2022_4955_MOESM33_ESM.tif]

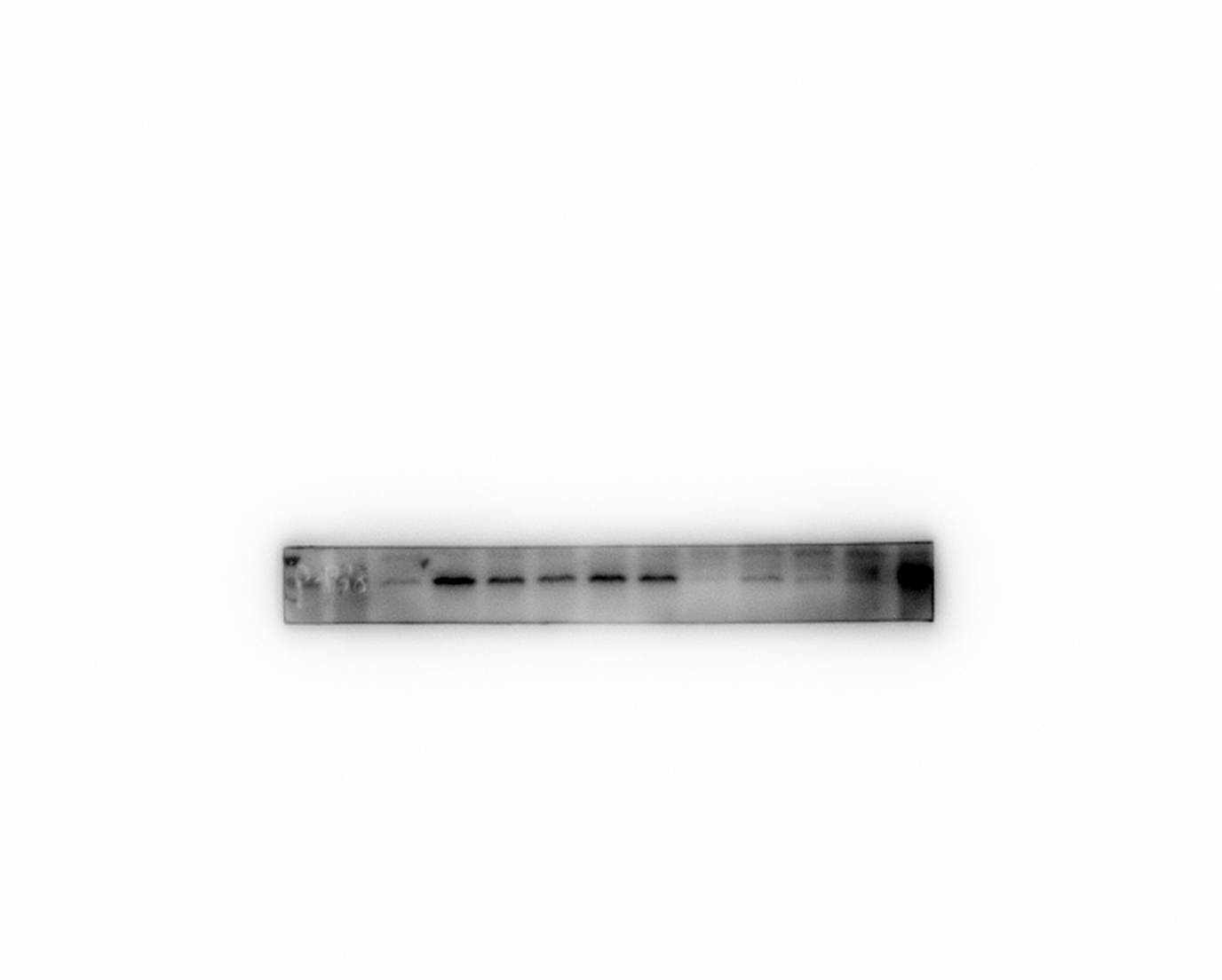

Supplement: Supplementary file 34 — Supplemental Material [file 41419_2022_4955_MOESM34_ESM.tif]

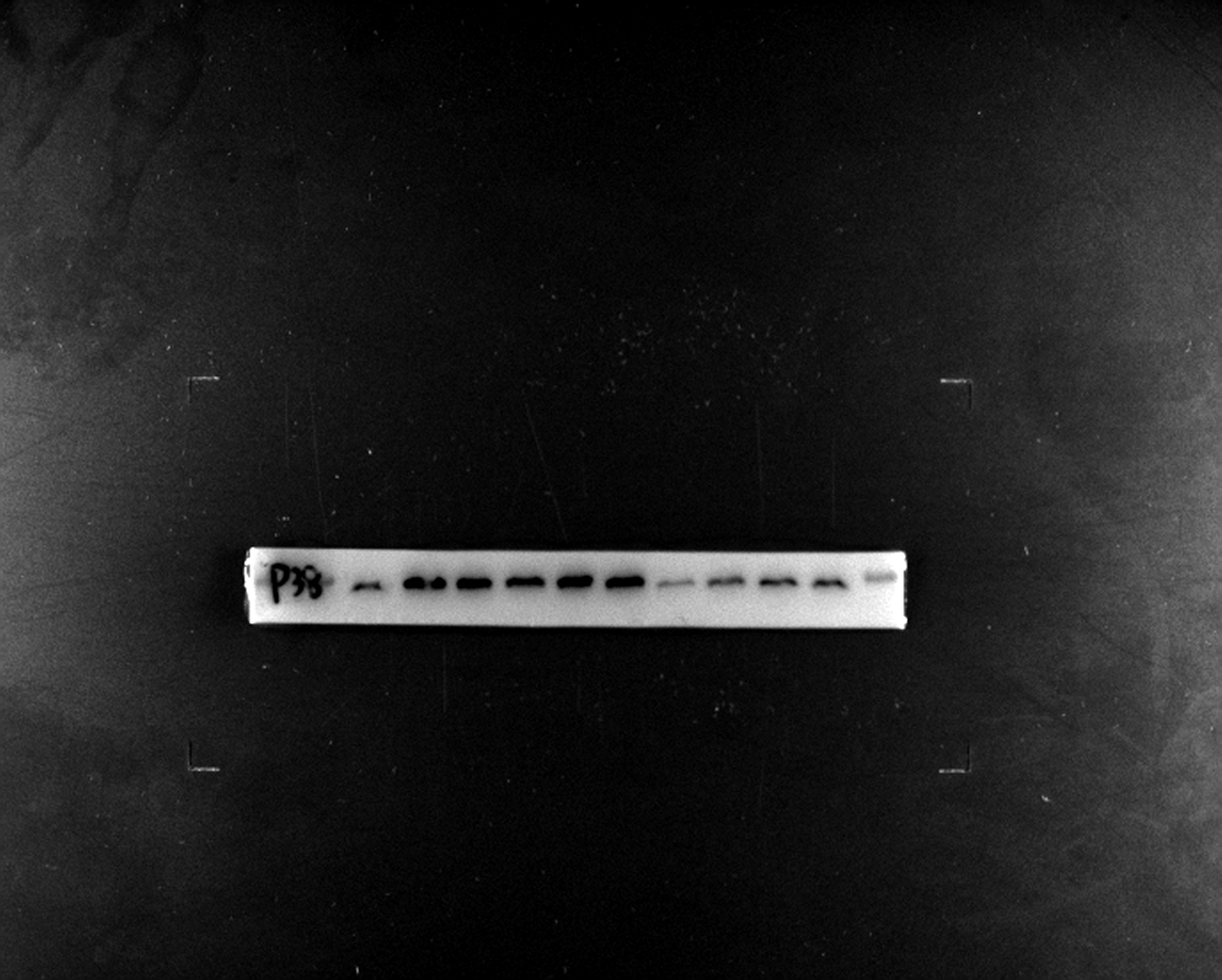

Supplement: Supplementary file 35 — Supplemental Material [file 41419_2022_4955_MOESM35_ESM.tif]

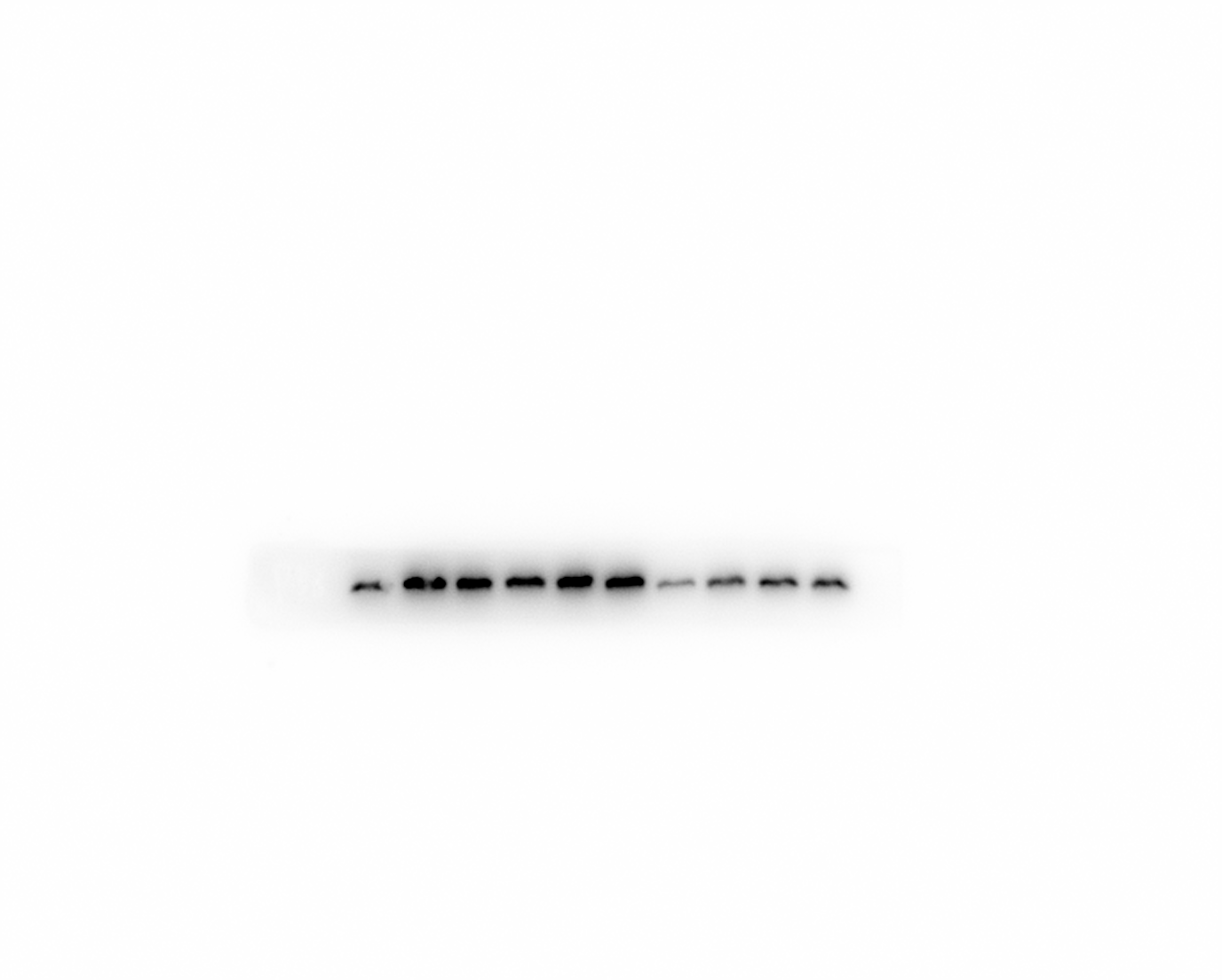

Supplement: Supplementary file 36 — Supplemental Material [file 41419_2022_4955_MOESM36_ESM.tif]

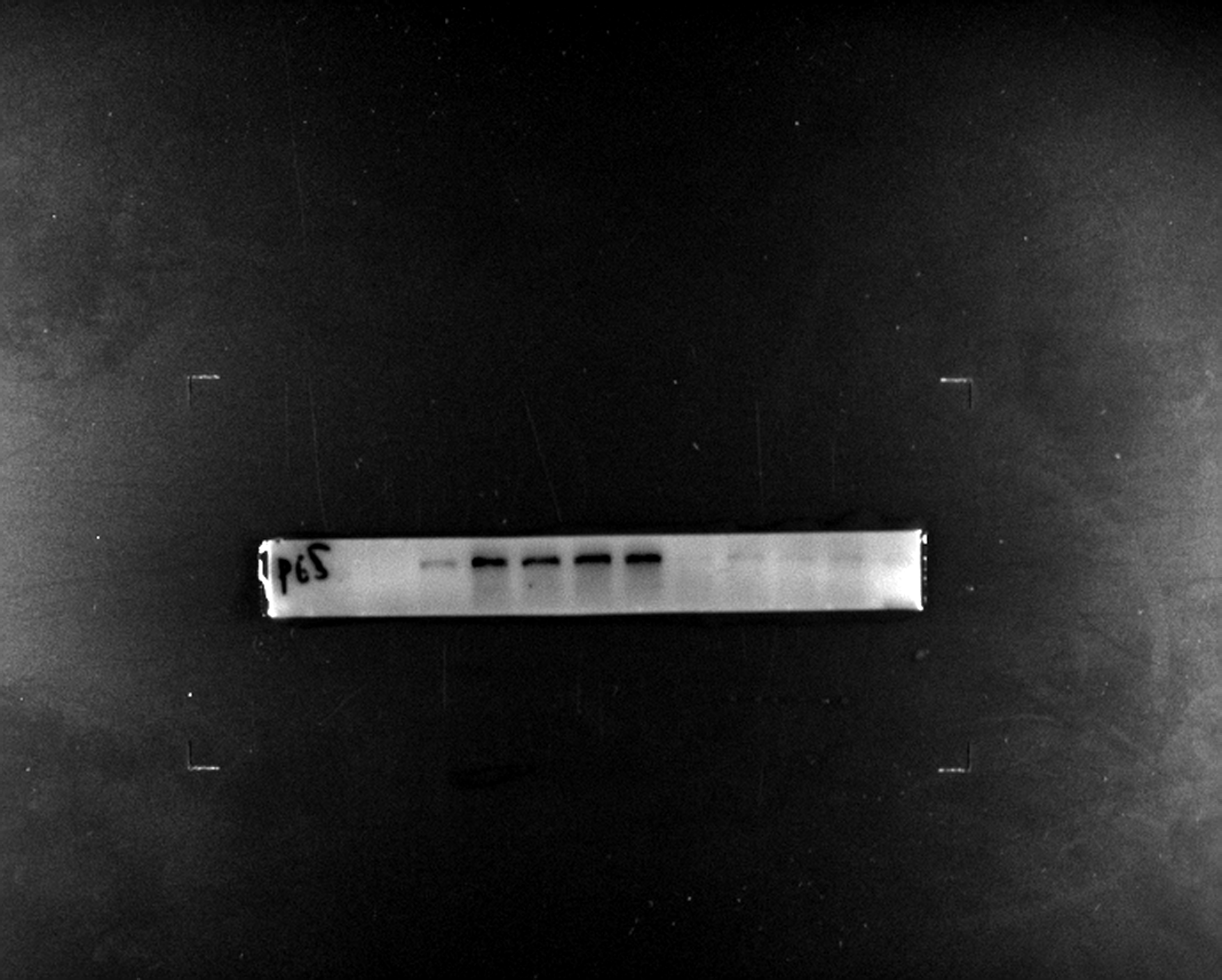

Supplement: Supplementary file 37 — Supplemental Material [file 41419_2022_4955_MOESM37_ESM.tif]

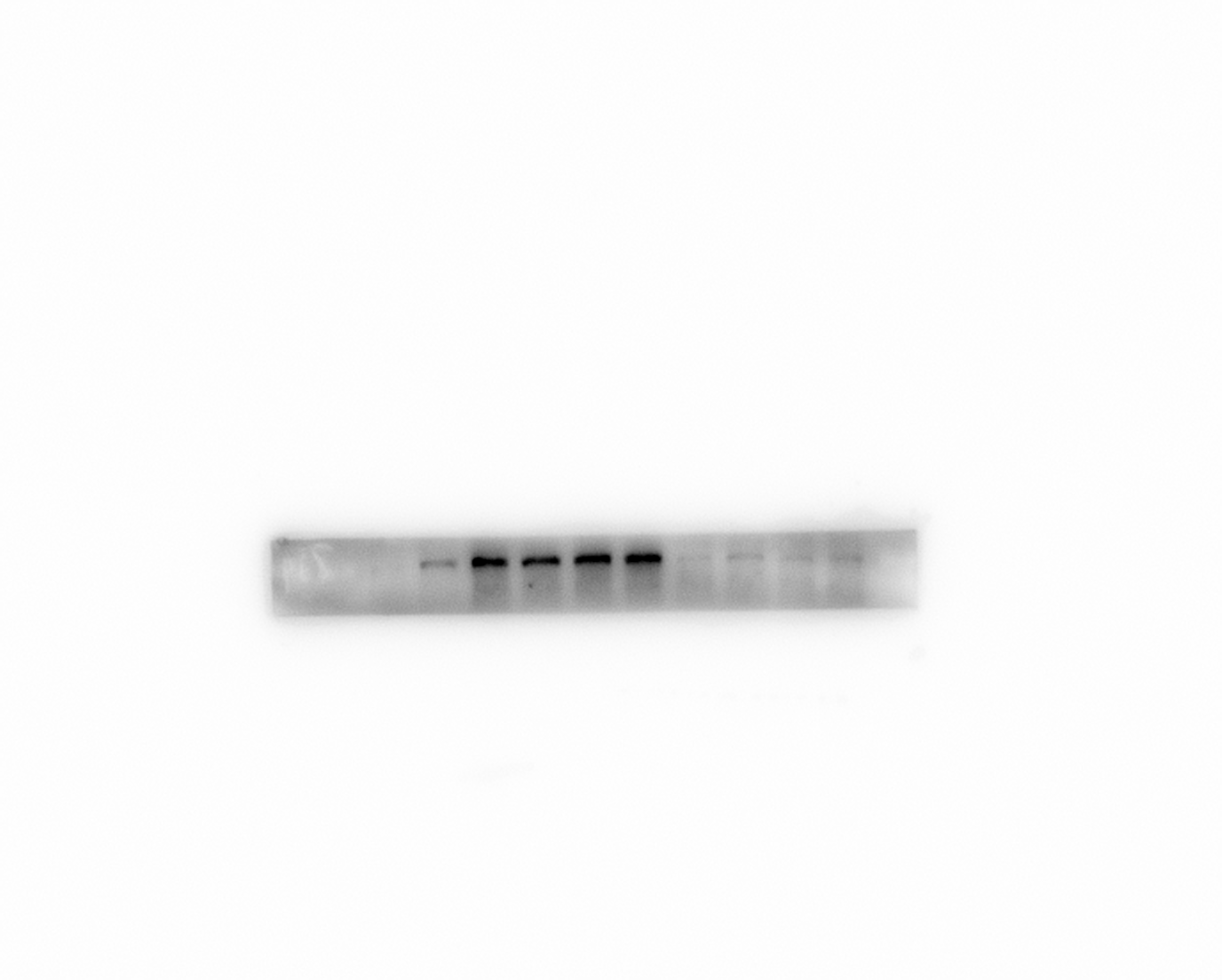

Supplement: Supplementary file 38 — Supplemental Material [file 41419_2022_4955_MOESM38_ESM.tif]

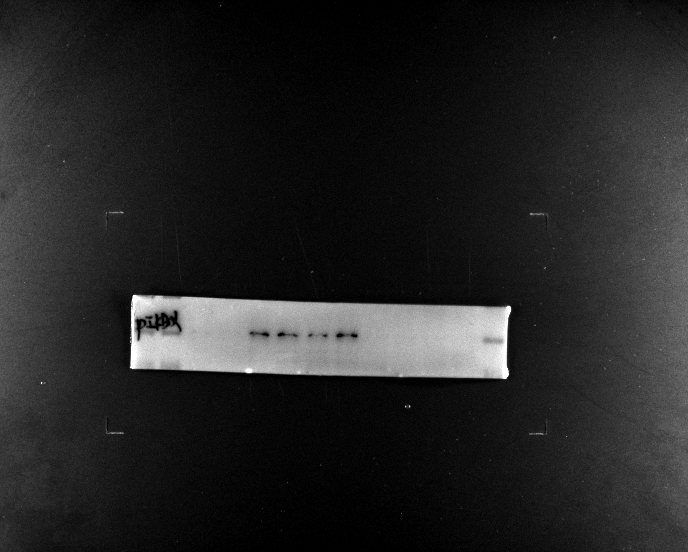

Supplement: Supplementary file 39 — Supplemental Material [file 41419_2022_4955_MOESM39_ESM.tif]

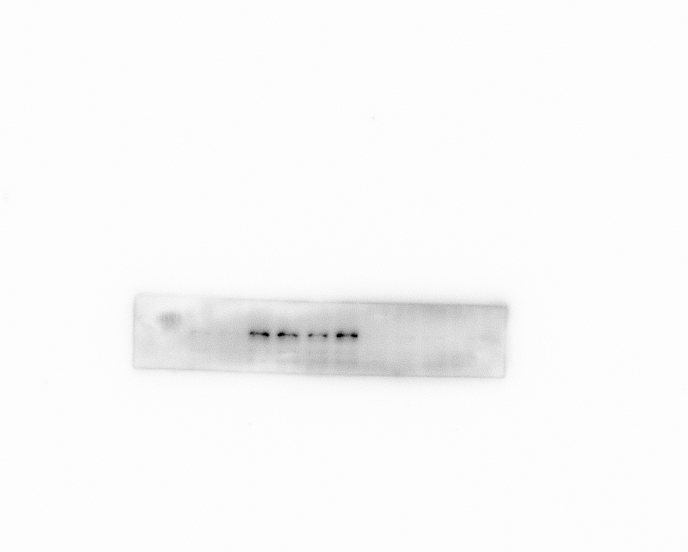

Supplement: Supplementary file 40 — Supplemental Material [file 41419_2022_4955_MOESM40_ESM.tif]

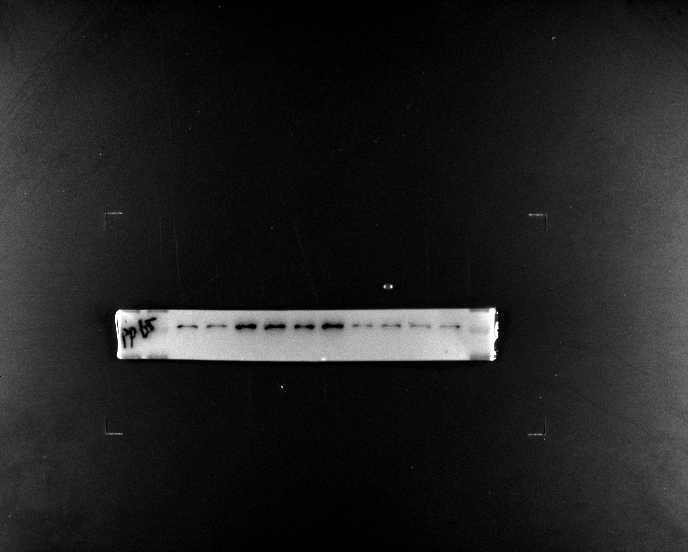

Supplement: Supplementary file 41 — Supplemental Material [file 41419_2022_4955_MOESM41_ESM.tif]

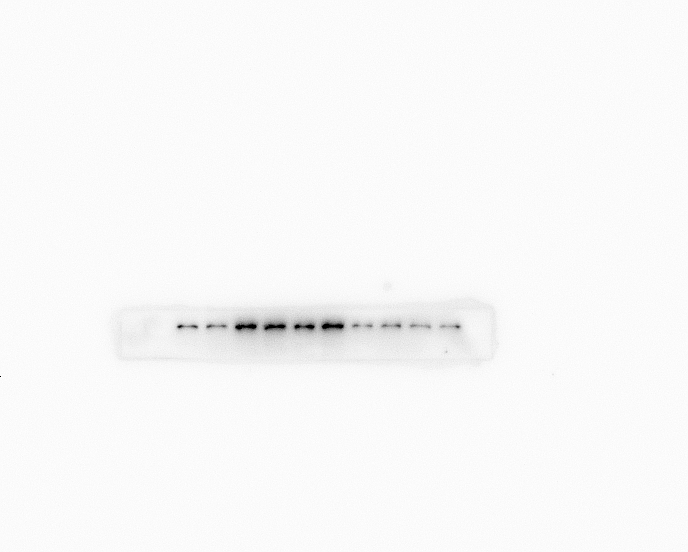

Supplement: Supplementary file 42 — Supplemental Material [file 41419_2022_4955_MOESM42_ESM.tif]

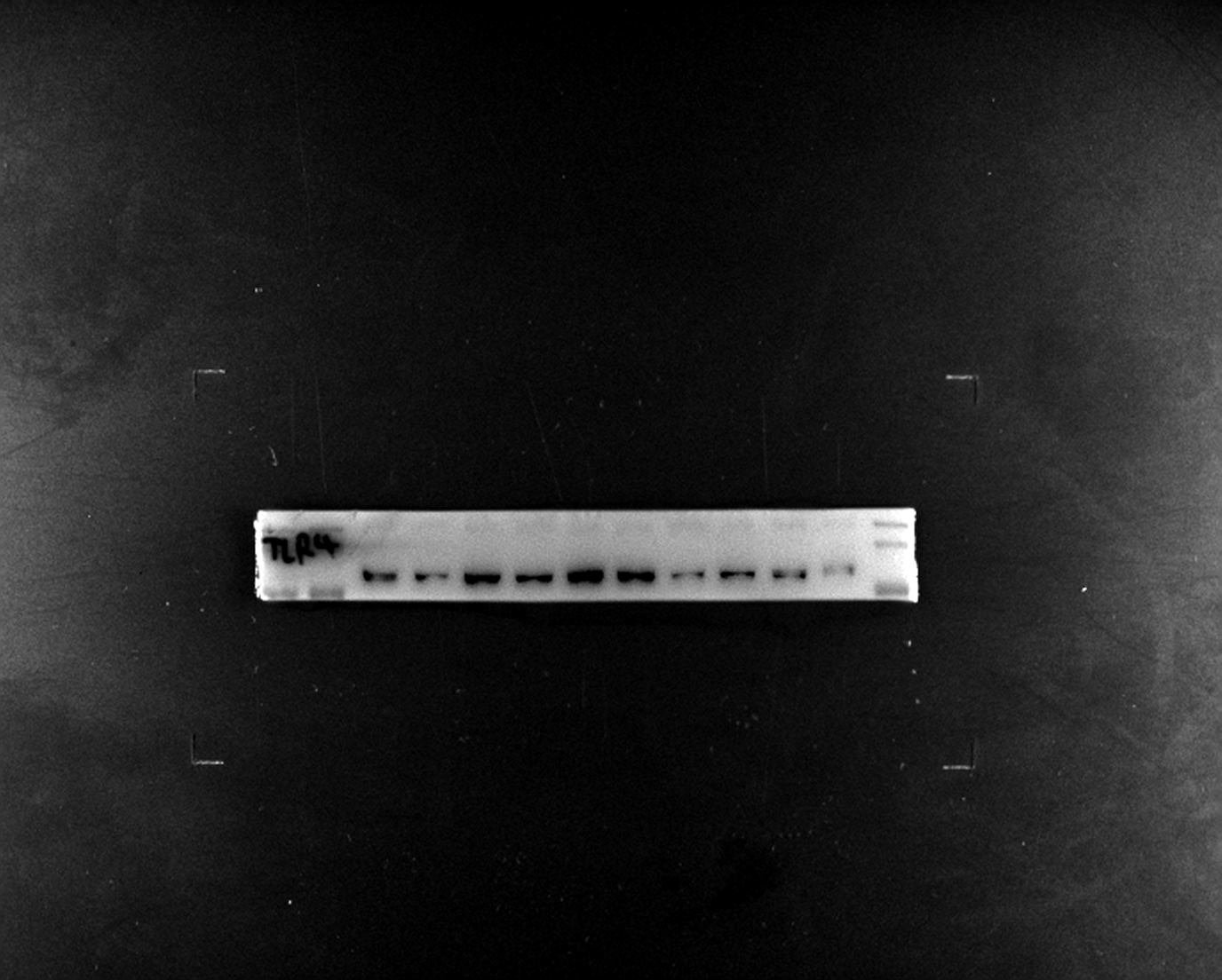

Supplement: Supplementary file 43 — Supplemental Material [file 41419_2022_4955_MOESM43_ESM.tif]

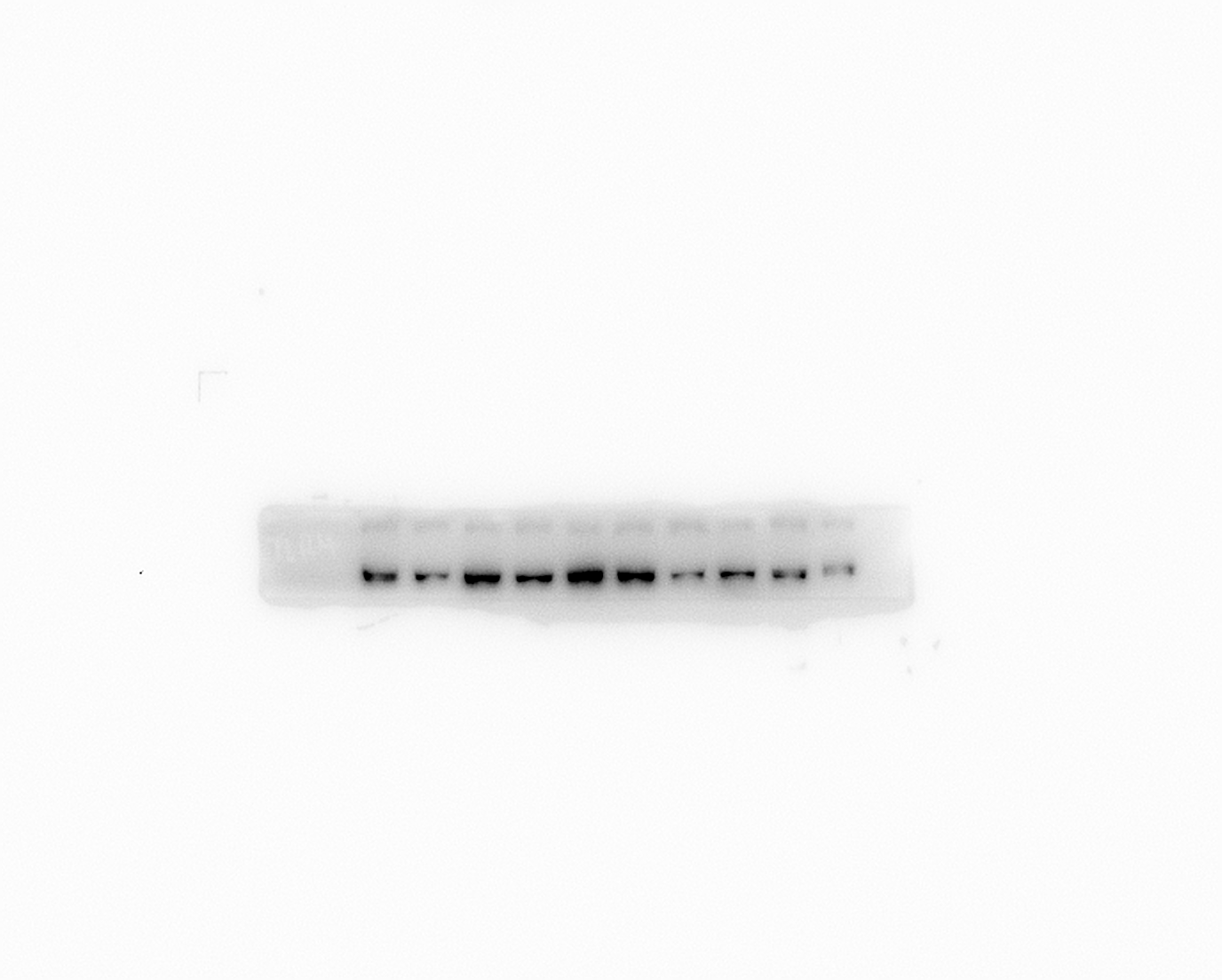

Supplement: Supplementary file 44 — Supplemental Material [file 41419_2022_4955_MOESM44_ESM.tif]

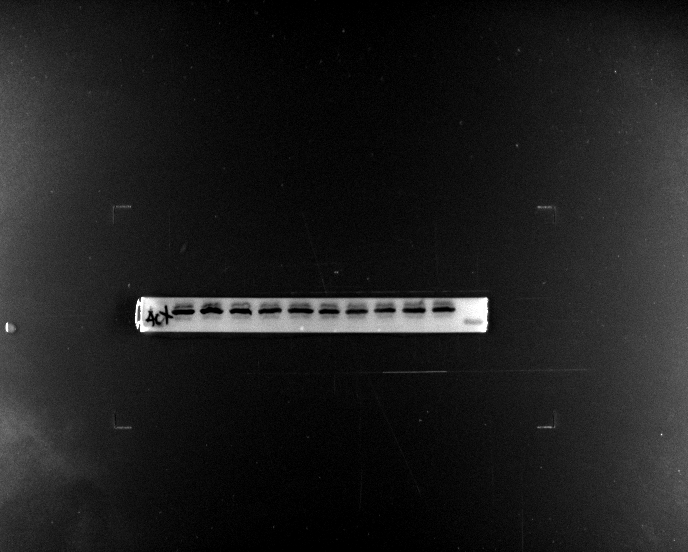

Supplement: Supplementary file 45 — Supplemental Material [file 41419_2022_4955_MOESM45_ESM.tif]

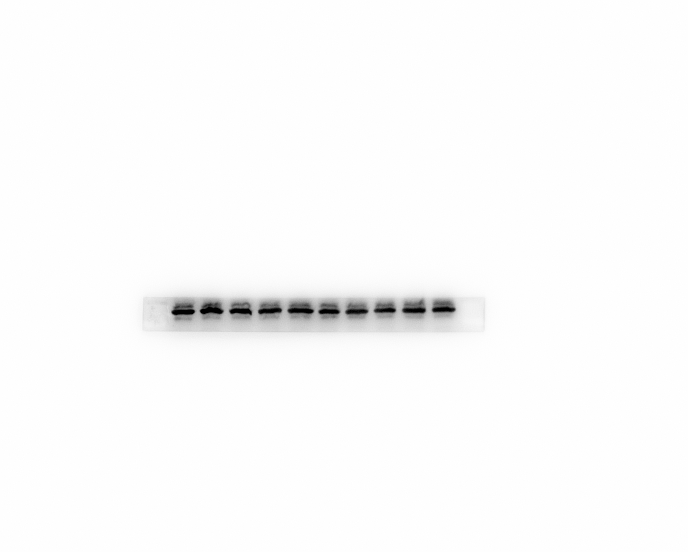

Supplement: Supplementary file 46 — Supplemental Material [file 41419_2022_4955_MOESM46_ESM.tif]

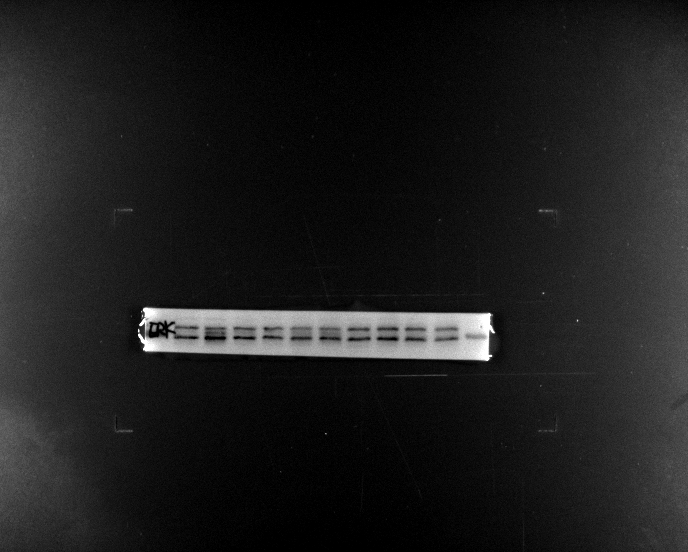

Supplement: Supplementary file 47 — Supplemental Material [file 41419_2022_4955_MOESM47_ESM.tif]

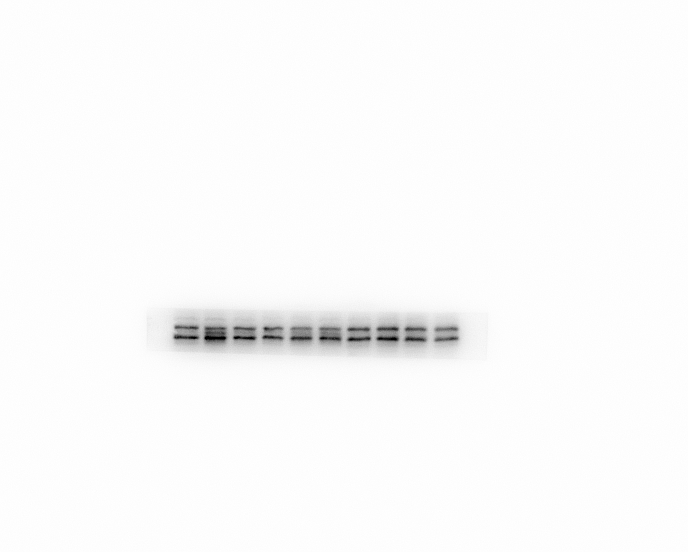

Supplement: Supplementary file 48 — Supplemental Material [file 41419_2022_4955_MOESM48_ESM.tif]

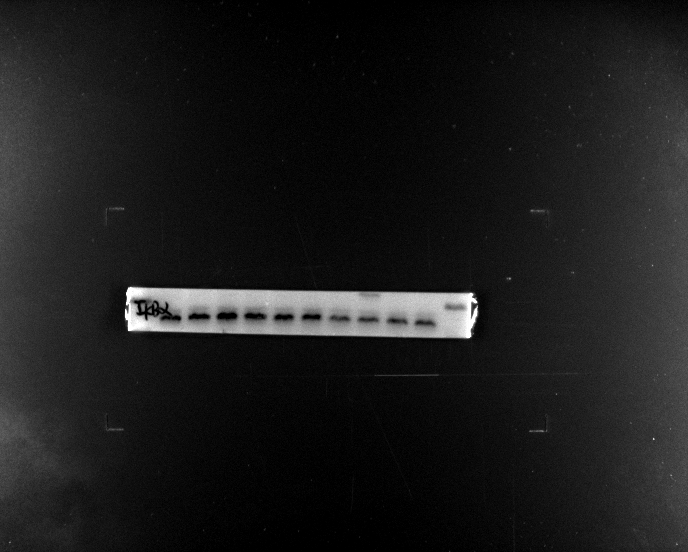

Supplement: Supplementary file 49 — Supplemental Material [file 41419_2022_4955_MOESM49_ESM.tif]

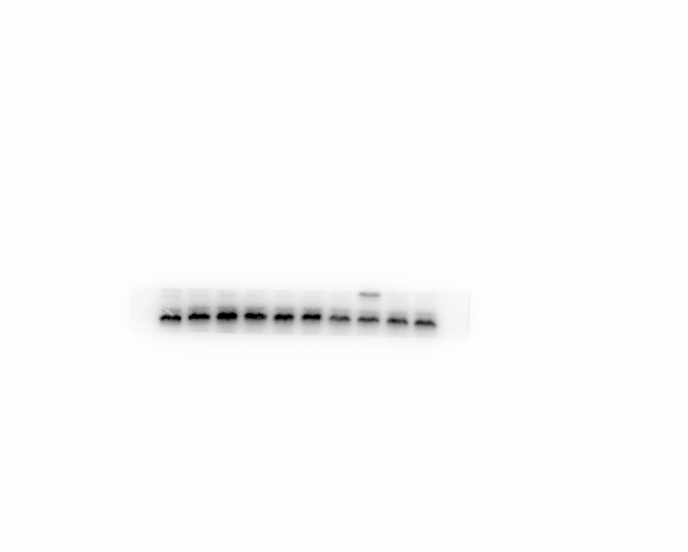

Supplement: Supplementary file 50 — Supplemental Material [file 41419_2022_4955_MOESM50_ESM.tif]

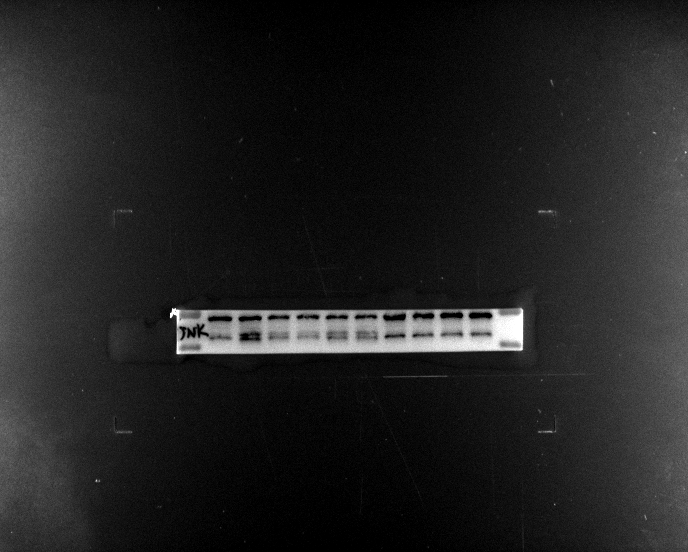

Supplement: Supplementary file 51 — Supplemental Material [file 41419_2022_4955_MOESM51_ESM.tif]

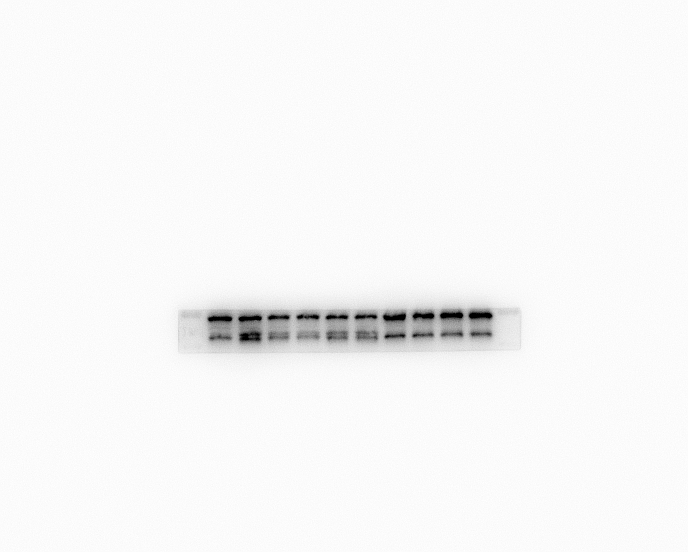

Supplement: Supplementary file 52 — Supplemental Material [file 41419_2022_4955_MOESM52_ESM.tif]

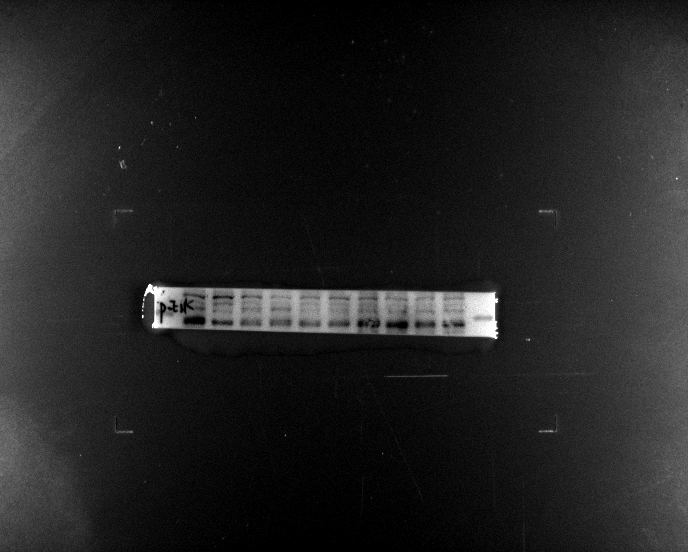

Supplement: Supplementary file 53 — Supplemental Material [file 41419_2022_4955_MOESM53_ESM.tif]

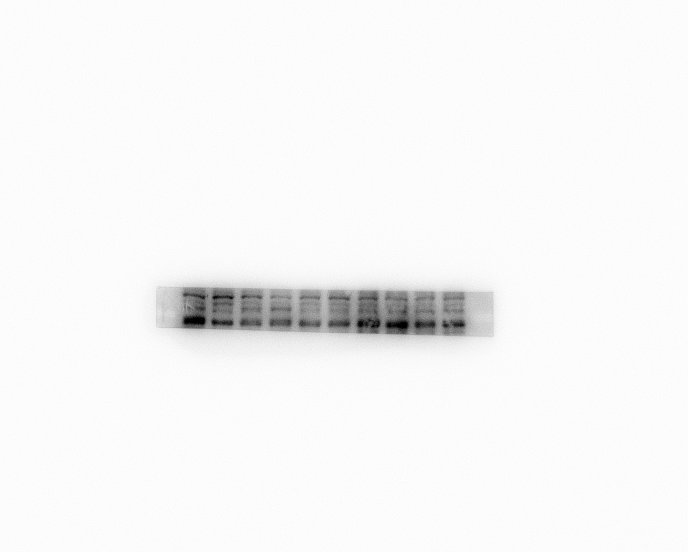

Supplement: Supplementary file 54 — Supplemental Material [file 41419_2022_4955_MOESM54_ESM.tif]

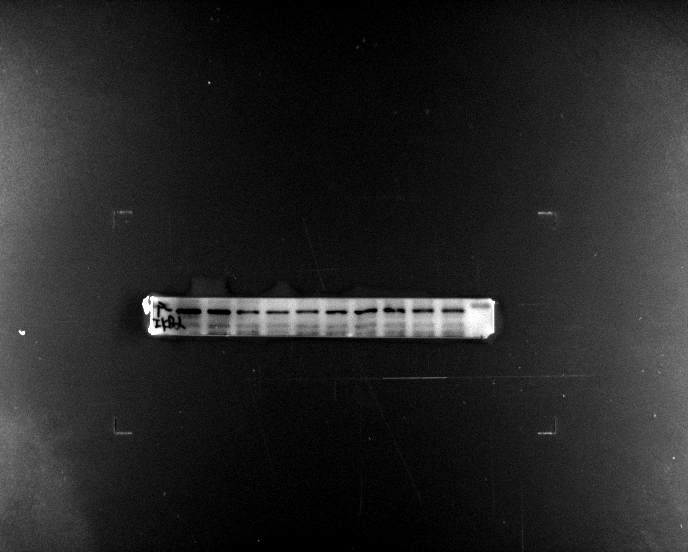

Supplement: Supplementary file 55 — Supplemental Material [file 41419_2022_4955_MOESM55_ESM.tif]

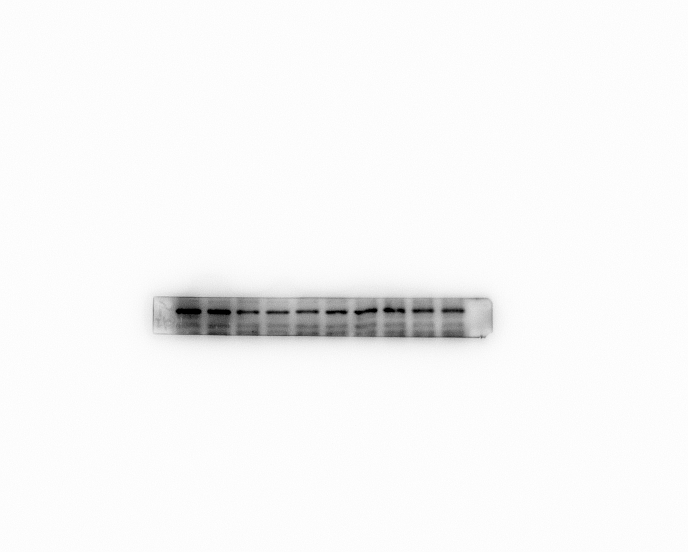

Supplement: Supplementary file 56 — Supplemental Material [file 41419_2022_4955_MOESM56_ESM.tif]

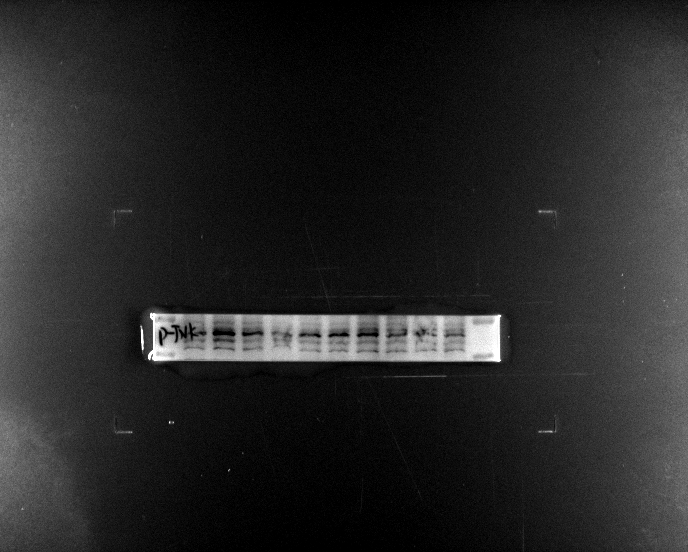

Supplement: Supplementary file 57 — Supplemental Material [file 41419_2022_4955_MOESM57_ESM.tif]

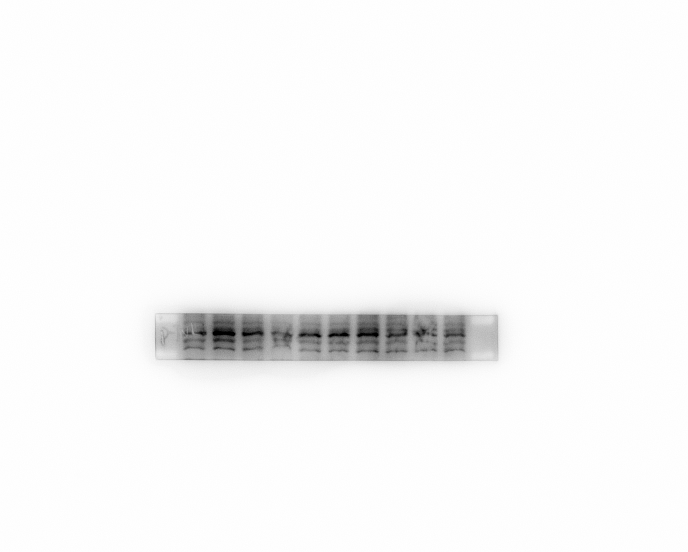

Supplement: Supplementary file 58 — Supplemental Material [file 41419_2022_4955_MOESM58_ESM.tif]

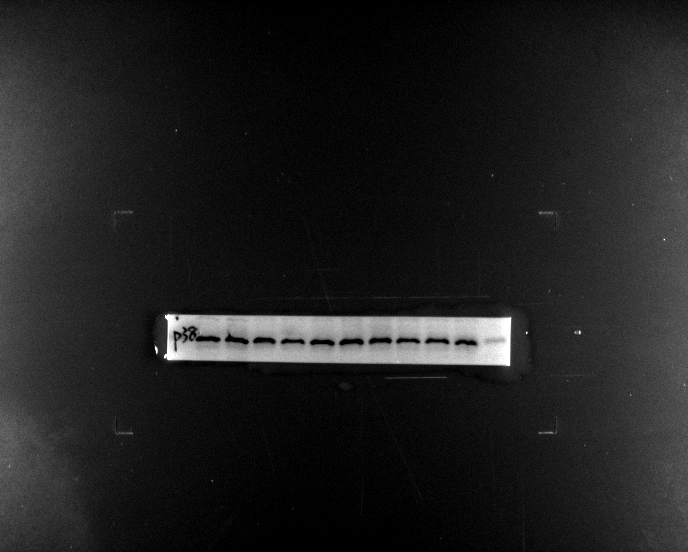

Supplement: Supplementary file 59 — Supplemental Material [file 41419_2022_4955_MOESM59_ESM.tif]

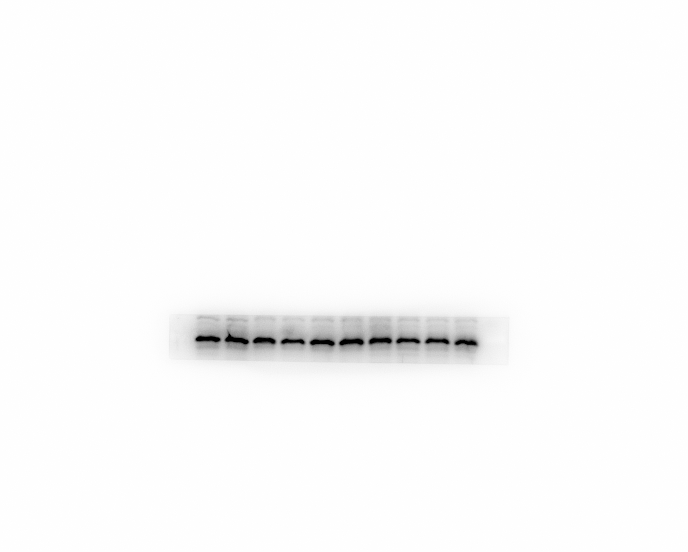

Supplement: Supplementary file 60 — Supplemental Material [file 41419_2022_4955_MOESM60_ESM.tif]

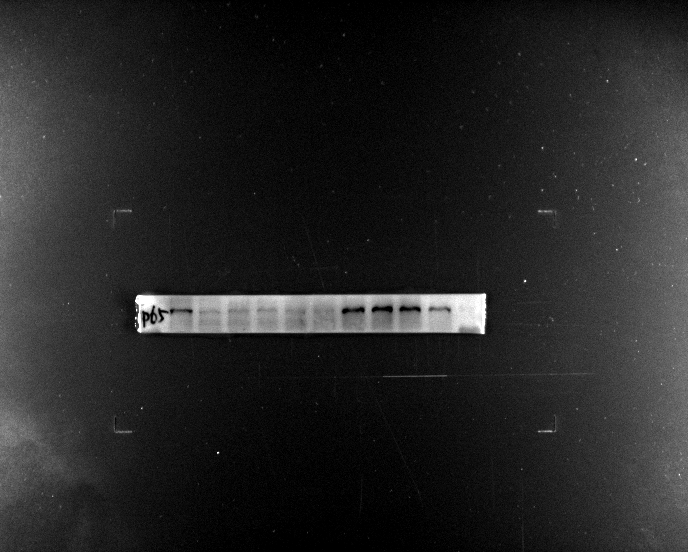

Supplement: Supplementary file 61 — Supplemental Material [file 41419_2022_4955_MOESM61_ESM.tif]

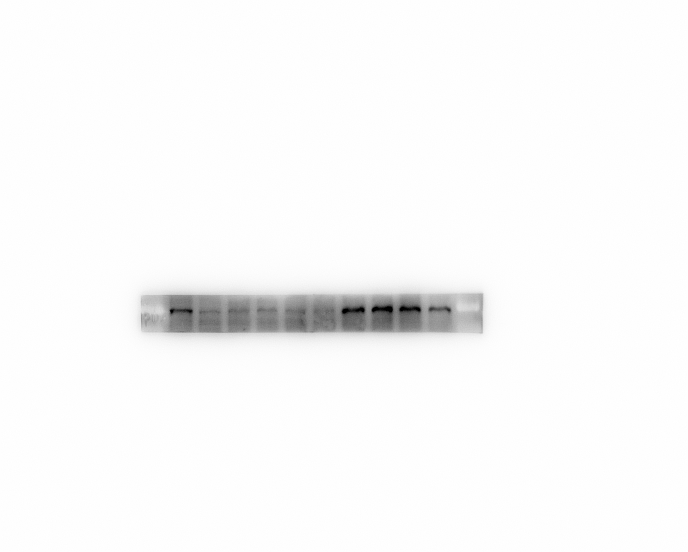

Supplement: Supplementary file 62 — Supplemental Material [file 41419_2022_4955_MOESM62_ESM.tif]

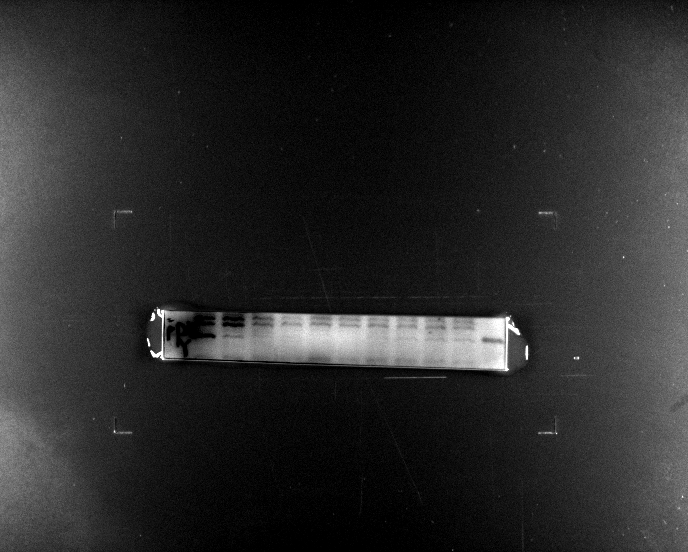

Supplement: Supplementary file 63 — Supplemental Material [file 41419_2022_4955_MOESM63_ESM.tif]

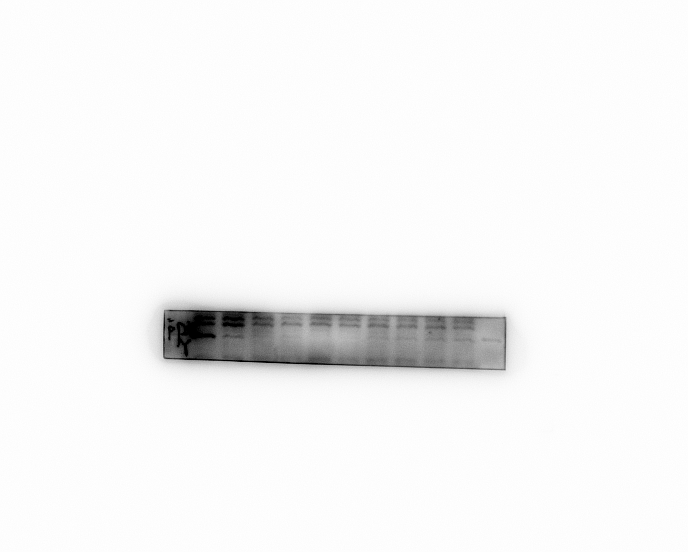

Supplement: Supplementary file 64 — Supplemental Material [file 41419_2022_4955_MOESM64_ESM.tif]

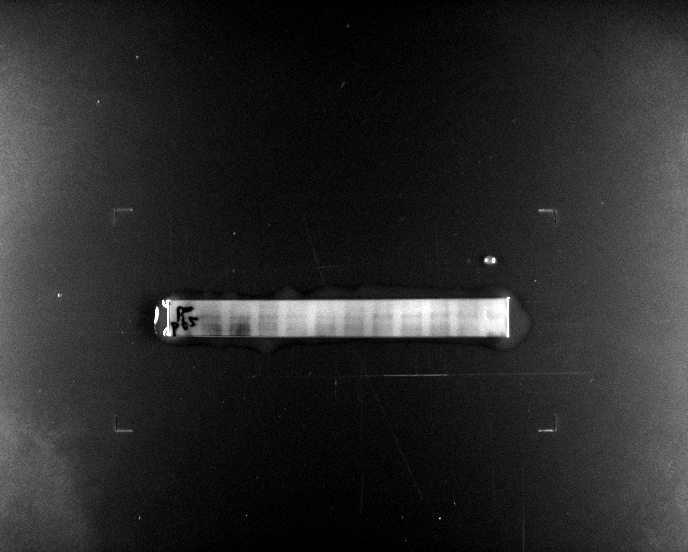

Supplement: Supplementary file 65 — Supplemental Material [file 41419_2022_4955_MOESM65_ESM.tif]

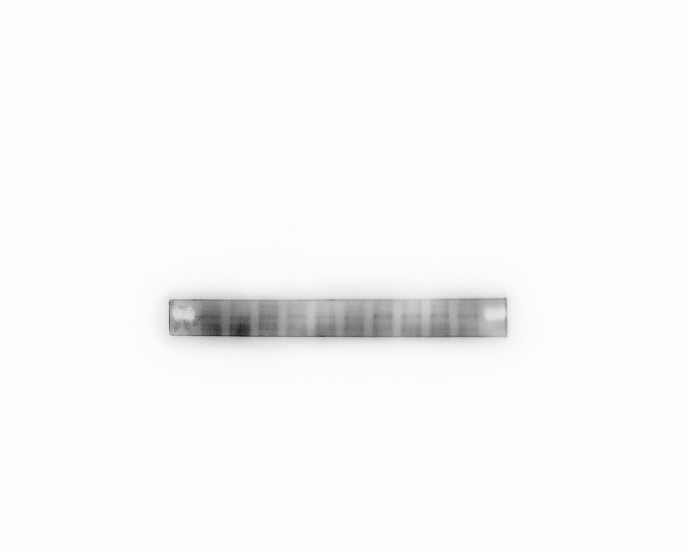

Supplement: Supplementary file 66 — Supplemental Material [file 41419_2022_4955_MOESM66_ESM.tif]

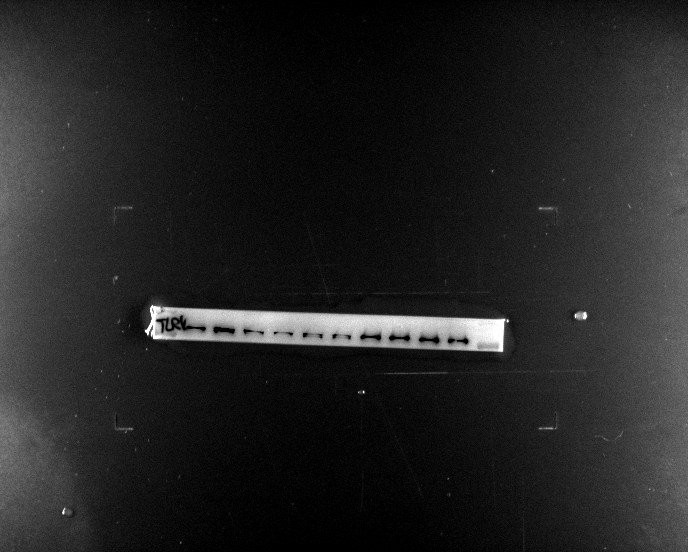

Supplement: Supplementary file 67 — Supplemental Material [file 41419_2022_4955_MOESM67_ESM.tif]

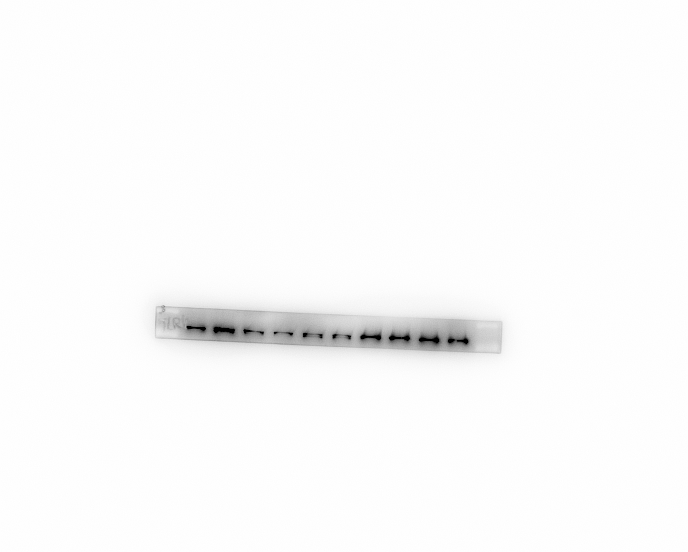

Supplement: Supplementary file 68 — Supplemental Material [file 41419_2022_4955_MOESM68_ESM.tif]

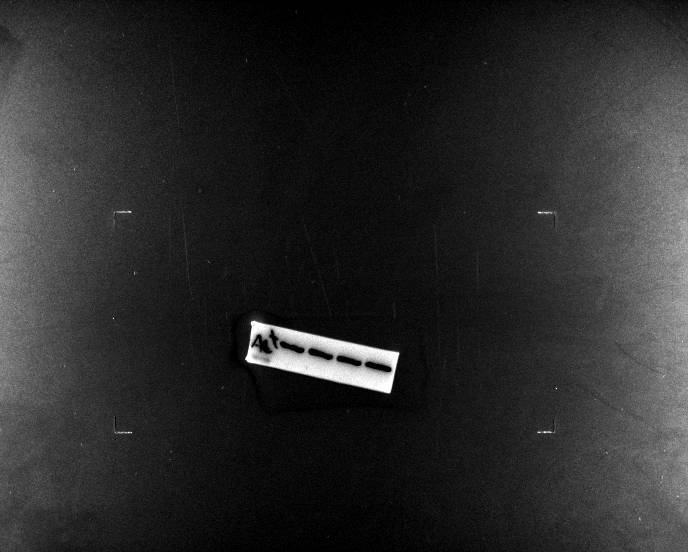

Supplement: Supplementary file 69 — Supplemental Material [file 41419_2022_4955_MOESM69_ESM.tif]

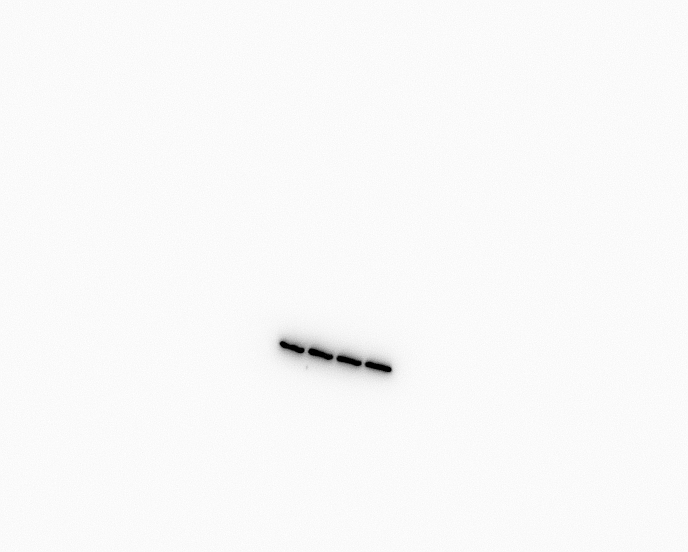

Supplement: Supplementary file 70 — Supplemental Material [file 41419_2022_4955_MOESM70_ESM.tif]

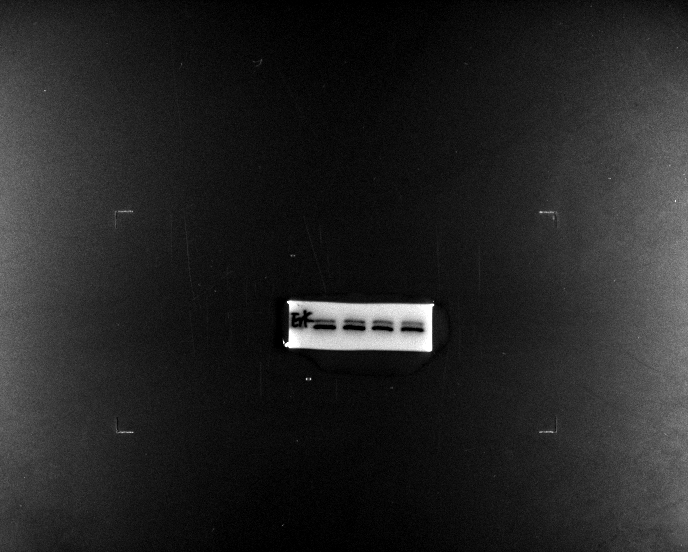

Supplement: Supplementary file 71 — Supplemental Material [file 41419_2022_4955_MOESM71_ESM.tif]

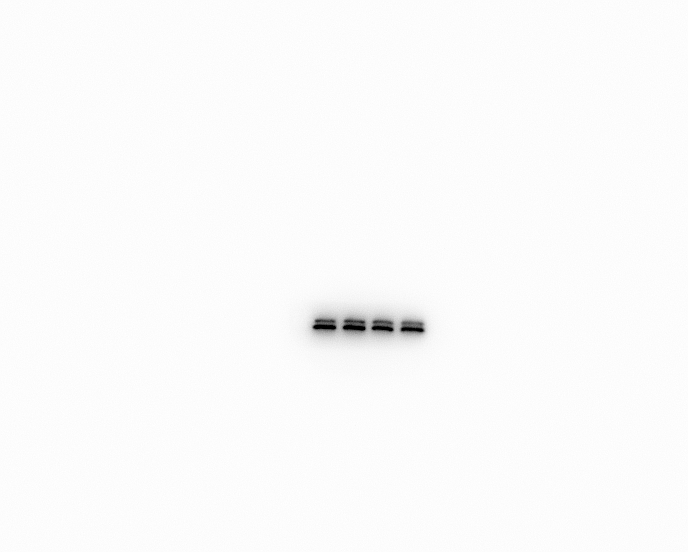

Supplement: Supplementary file 72 — Supplemental Material [file 41419_2022_4955_MOESM72_ESM.tif]

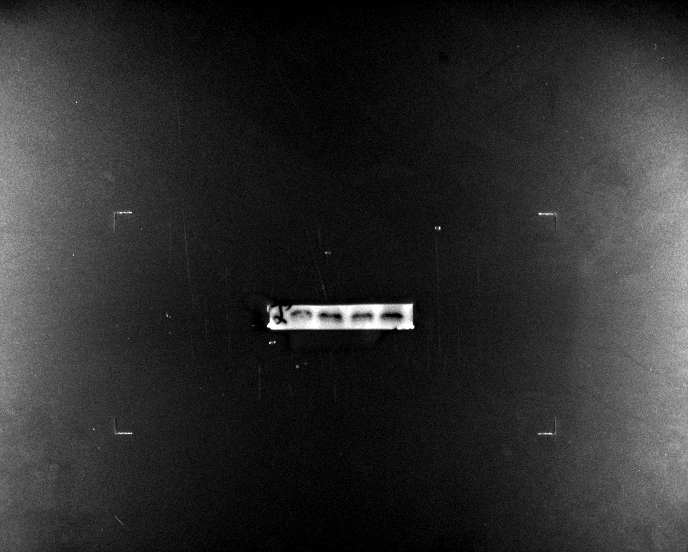

Supplement: Supplementary file 73 — Supplemental Material [file 41419_2022_4955_MOESM73_ESM.tif]

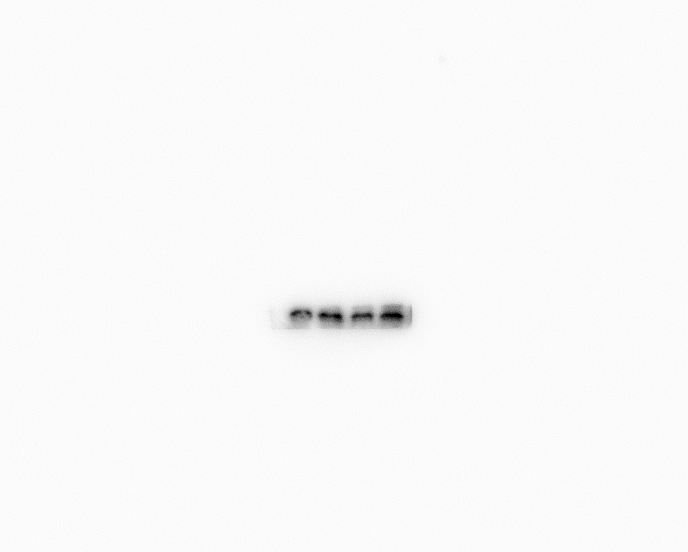

Supplement: Supplementary file 74 — Supplemental Material [file 41419_2022_4955_MOESM74_ESM.tif]

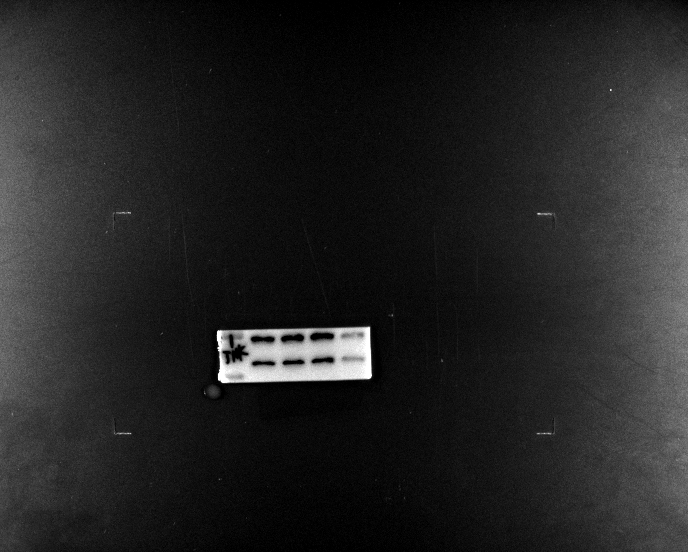

Supplement: Supplementary file 75 — Supplemental Material [file 41419_2022_4955_MOESM75_ESM.tif]

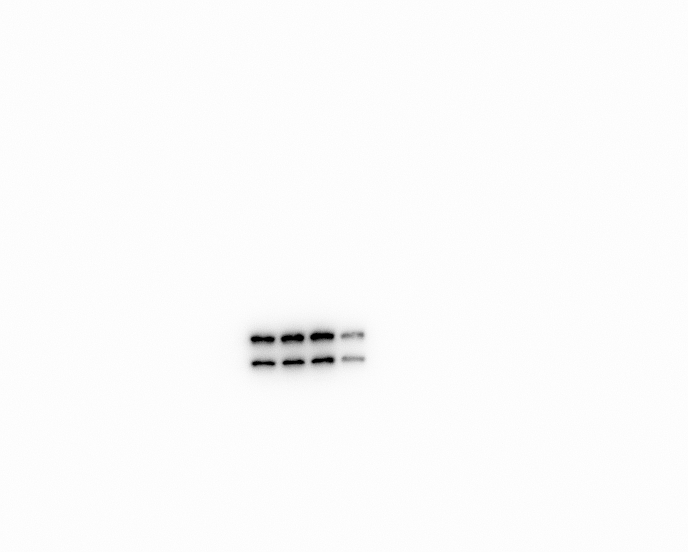

Supplement: Supplementary file 76 — Supplemental Material [file 41419_2022_4955_MOESM76_ESM.tif]

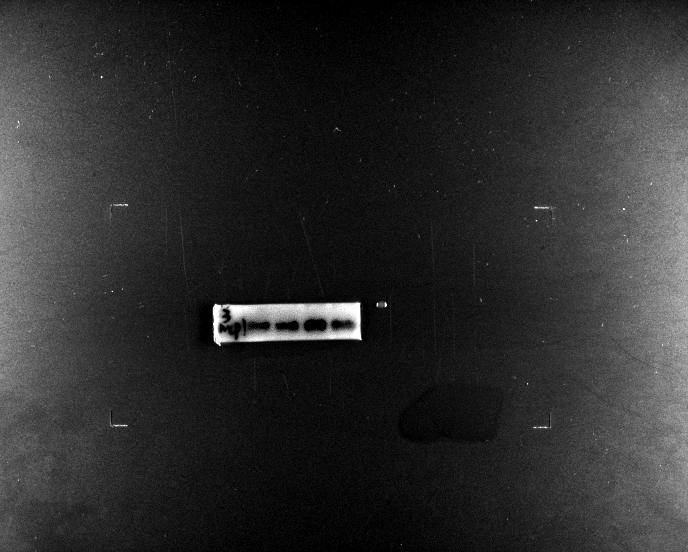

Supplement: Supplementary file 77 — Supplemental Material [file 41419_2022_4955_MOESM77_ESM.tif]

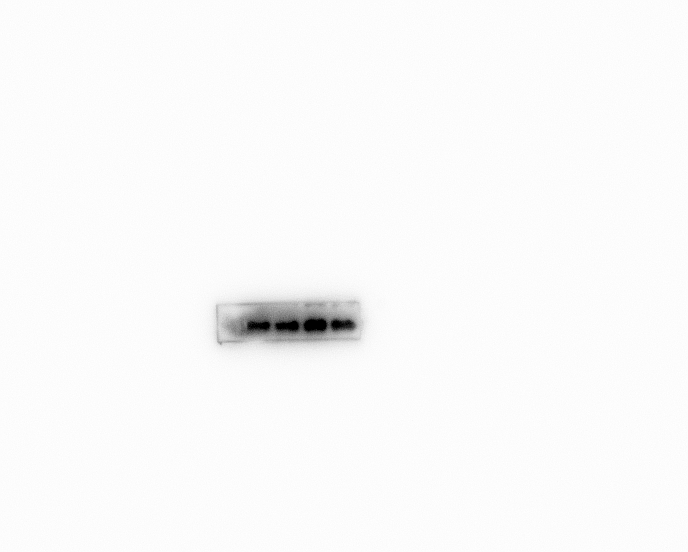

Supplement: Supplementary file 78 — Supplemental Material [file 41419_2022_4955_MOESM78_ESM.tif]

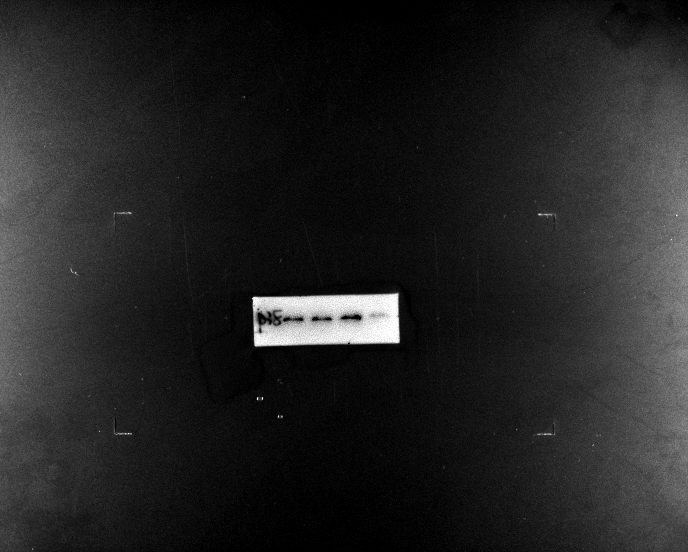

Supplement: Supplementary file 79 — Supplemental Material [file 41419_2022_4955_MOESM79_ESM.tif]

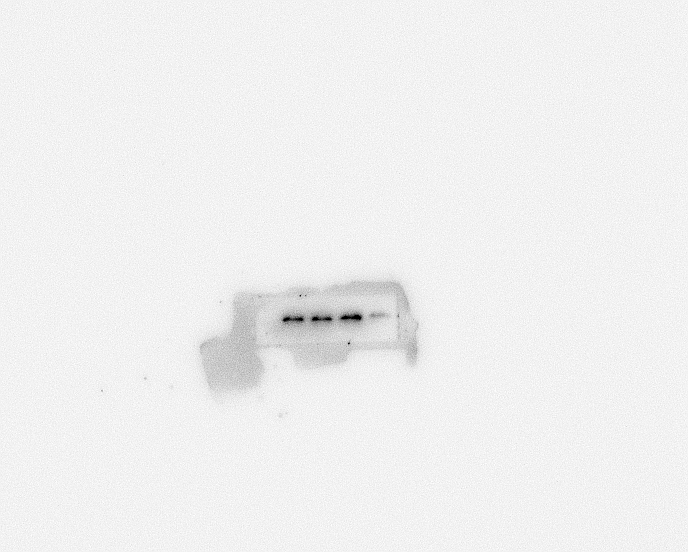

Supplement: Supplementary file 80 — Supplemental Material [file 41419_2022_4955_MOESM80_ESM.tif]

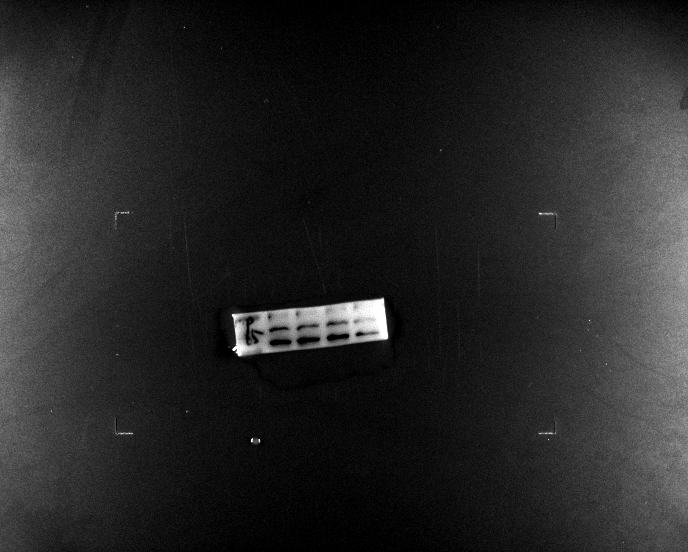

Supplement: Supplementary file 81 — Supplemental Material [file 41419_2022_4955_MOESM81_ESM.tif]

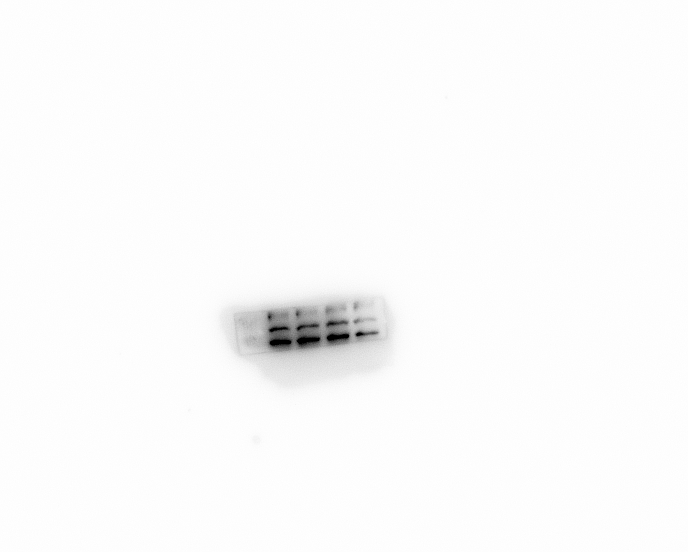

Supplement: Supplementary file 82 — Supplemental Material [file 41419_2022_4955_MOESM82_ESM.tif]

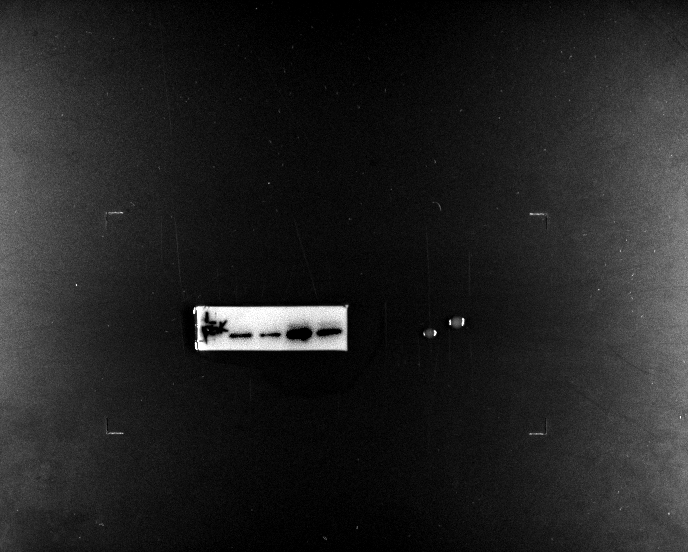

Supplement: Supplementary file 83 — Supplemental Material [file 41419_2022_4955_MOESM83_ESM.tif]

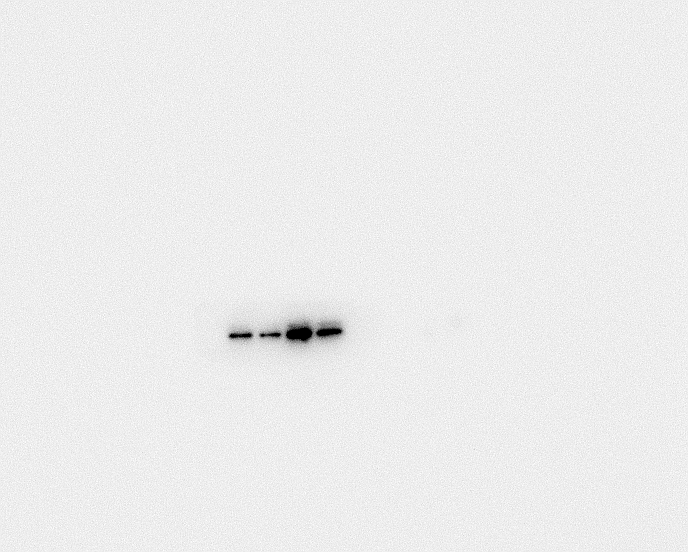

Supplement: Supplementary file 84 — Supplemental Material [file 41419_2022_4955_MOESM84_ESM.tif]

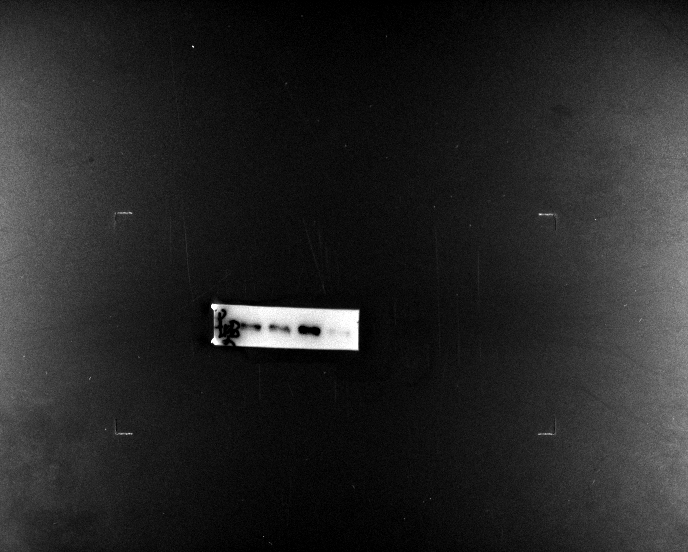

Supplement: Supplementary file 85 — Supplemental Material [file 41419_2022_4955_MOESM85_ESM.tif]

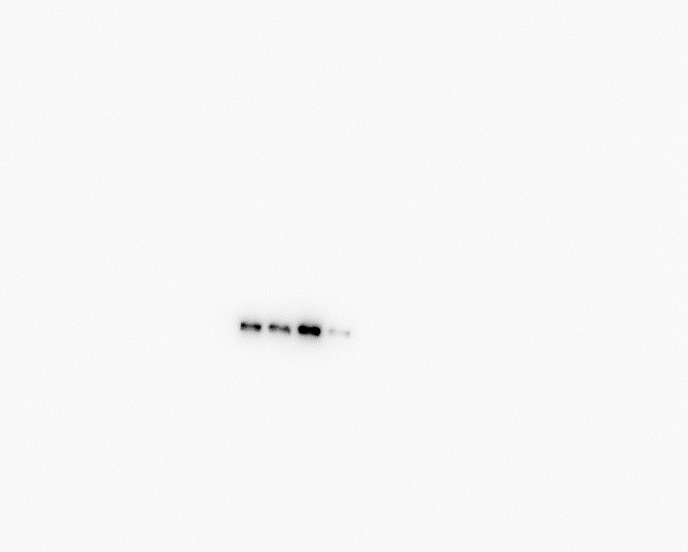

Supplement: Supplementary file 86 — Supplemental Material [file 41419_2022_4955_MOESM86_ESM.tif]

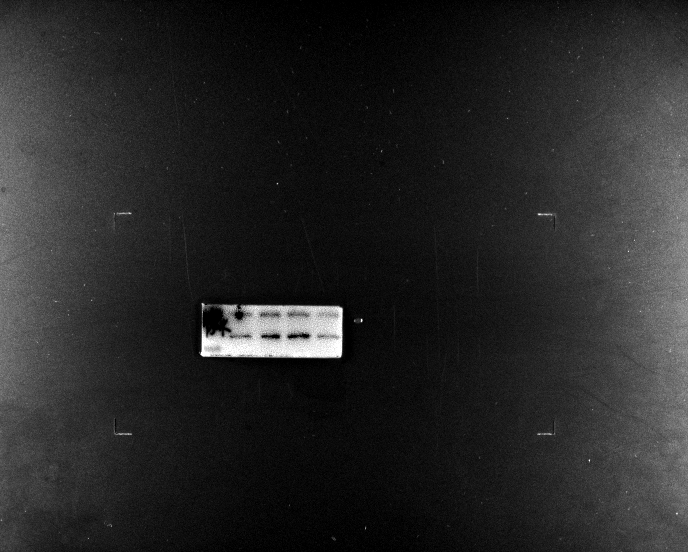

Supplement: Supplementary file 87 — Supplemental Material [file 41419_2022_4955_MOESM87_ESM.tif]

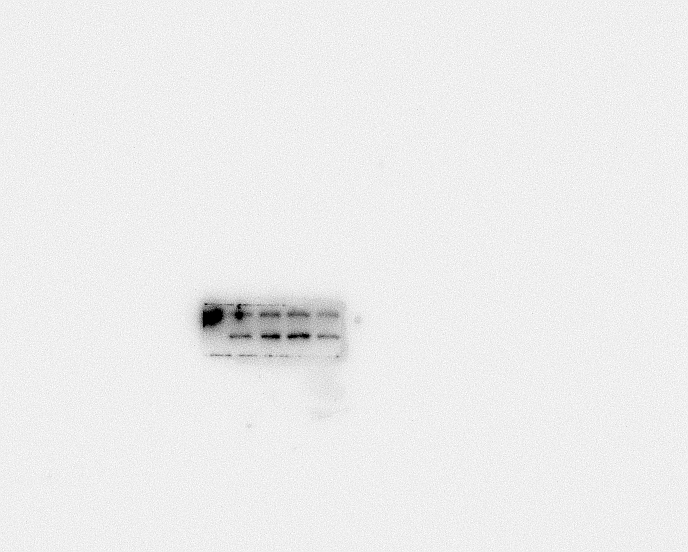

Supplement: Supplementary file 88 — Supplemental Material [file 41419_2022_4955_MOESM88_ESM.tif]

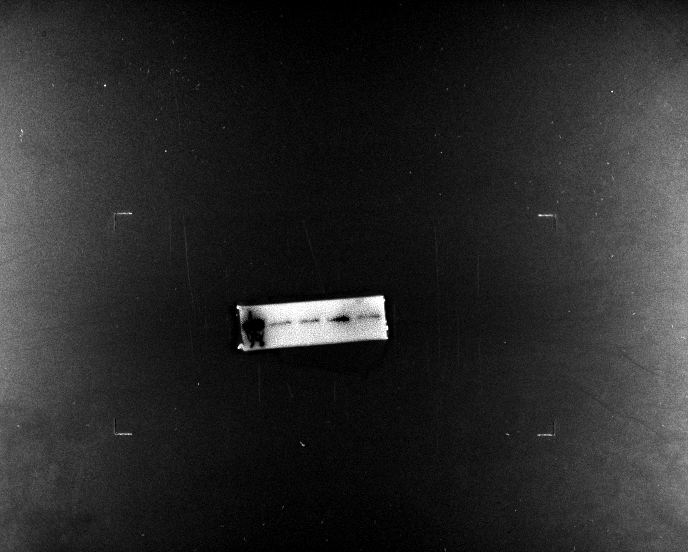

Supplement: Supplementary file 89 — Supplemental Material [file 41419_2022_4955_MOESM89_ESM.tif]

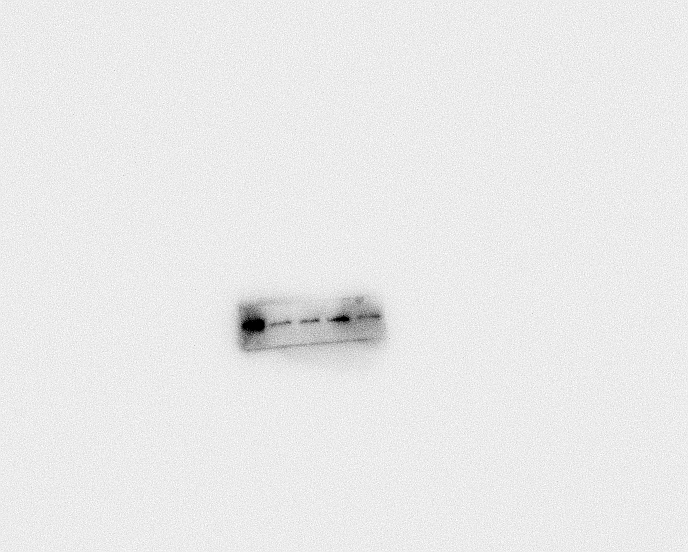

Supplement: Supplementary file 90 — Supplemental Material [file 41419_2022_4955_MOESM90_ESM.tif]

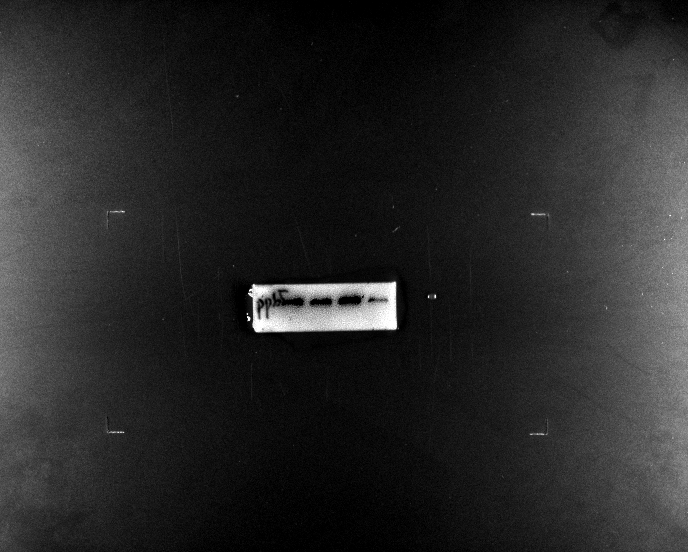

Supplement: Supplementary file 91 — Supplemental Material [file 41419_2022_4955_MOESM91_ESM.tif]

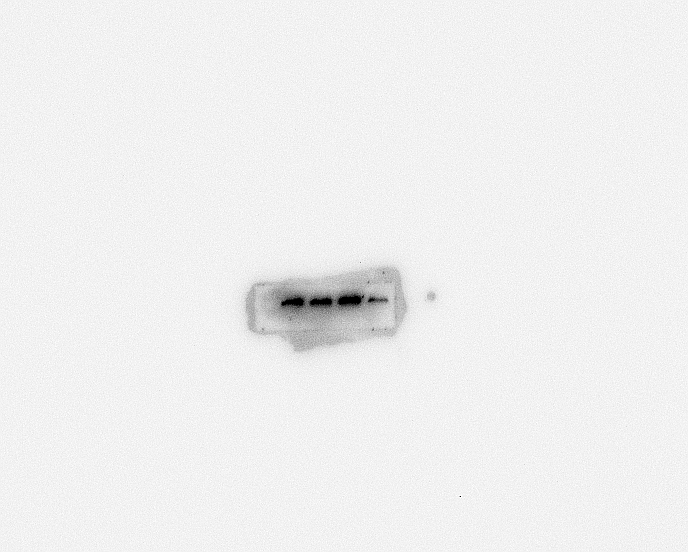

Supplement: Supplementary file 92 — Supplemental Material [file 41419_2022_4955_MOESM92_ESM.tif]

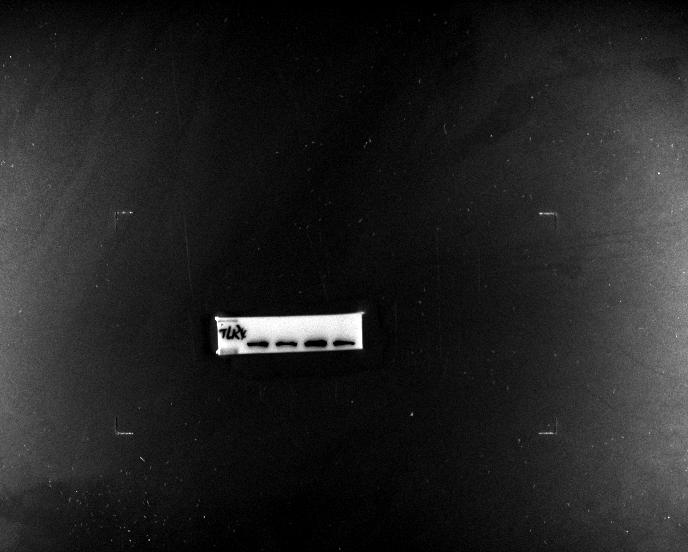

Supplement: Supplementary file 93 — Supplemental Material [file 41419_2022_4955_MOESM93_ESM.tif]

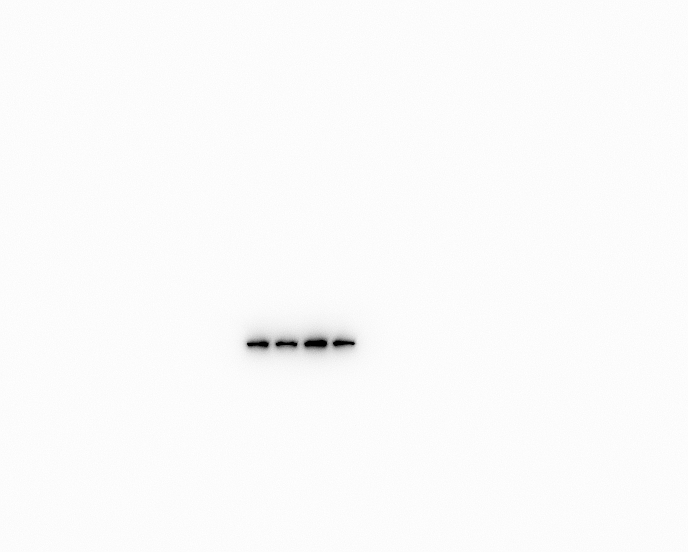

Supplement: Supplementary file 94 — Supplemental Material [file 41419_2022_4955_MOESM94_ESM.tif]

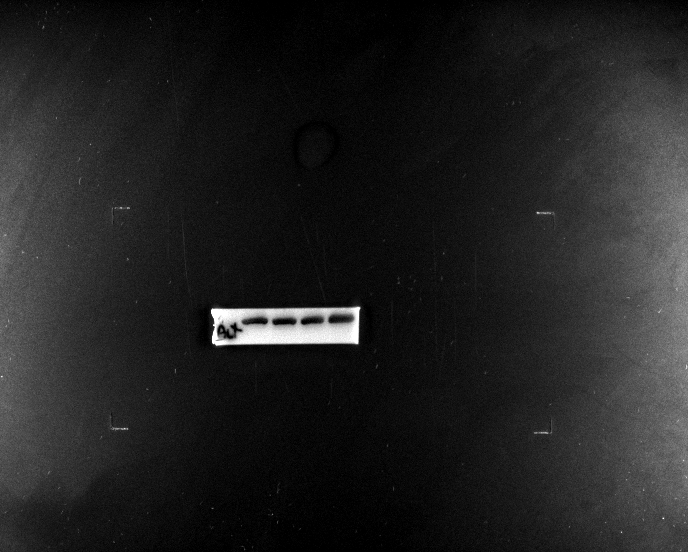

Supplement: Supplementary file 95 — Supplemental Material [file 41419_2022_4955_MOESM95_ESM.tif]

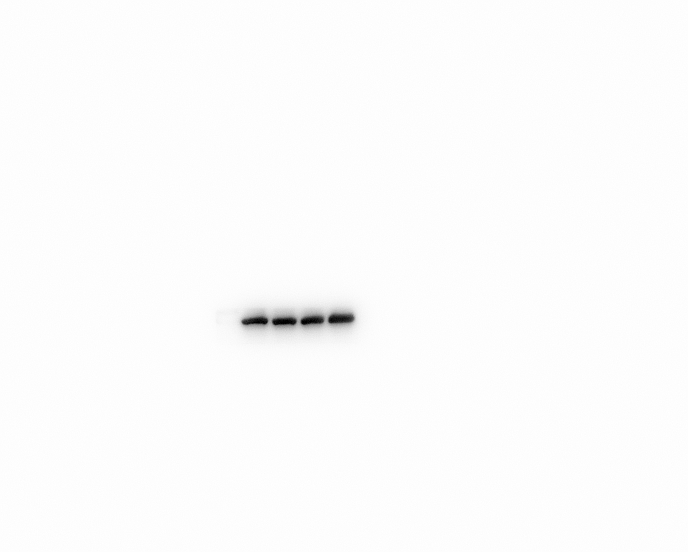

Supplement: Supplementary file 96 — Supplemental Material [file 41419_2022_4955_MOESM96_ESM.tif]

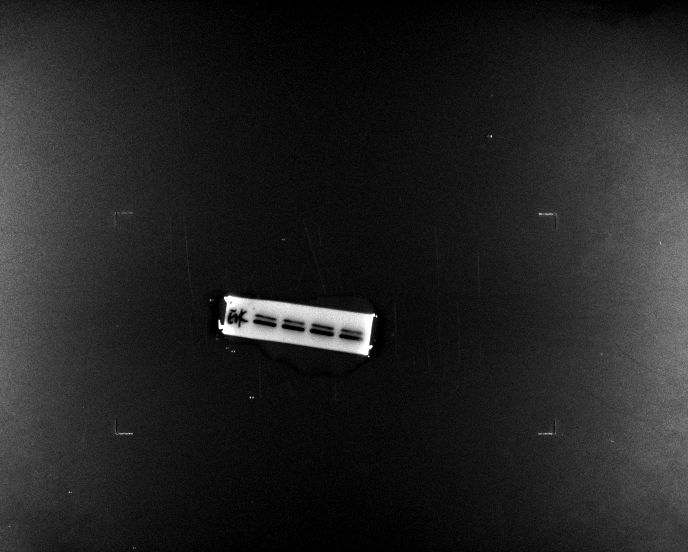

Supplement: Supplementary file 97 — Supplemental Material [file 41419_2022_4955_MOESM97_ESM.tif]

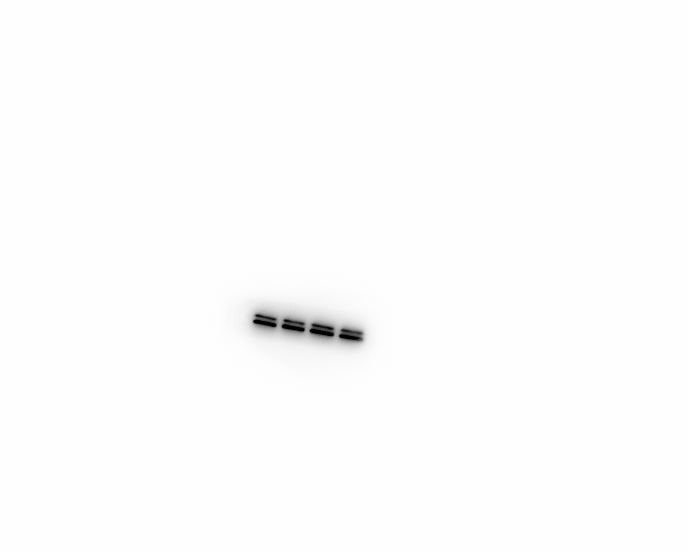

Supplement: Supplementary file 98 — Supplemental Material [file 41419_2022_4955_MOESM98_ESM.tif]

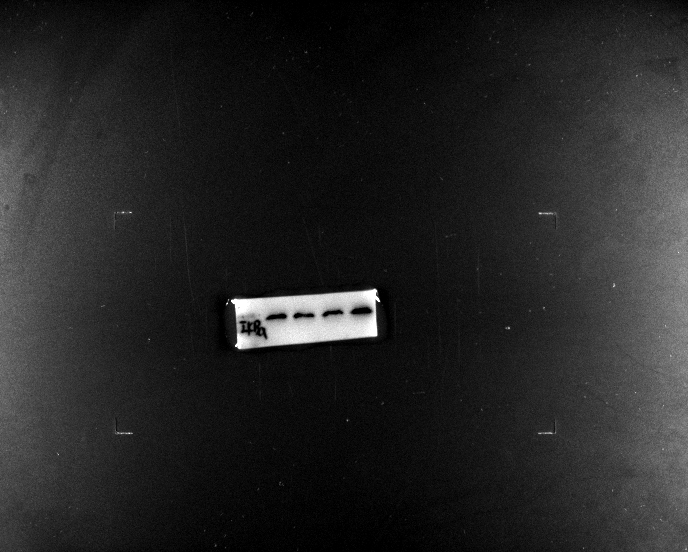

Supplement: Supplementary file 99 — Supplemental Material [file 41419_2022_4955_MOESM99_ESM.tif]

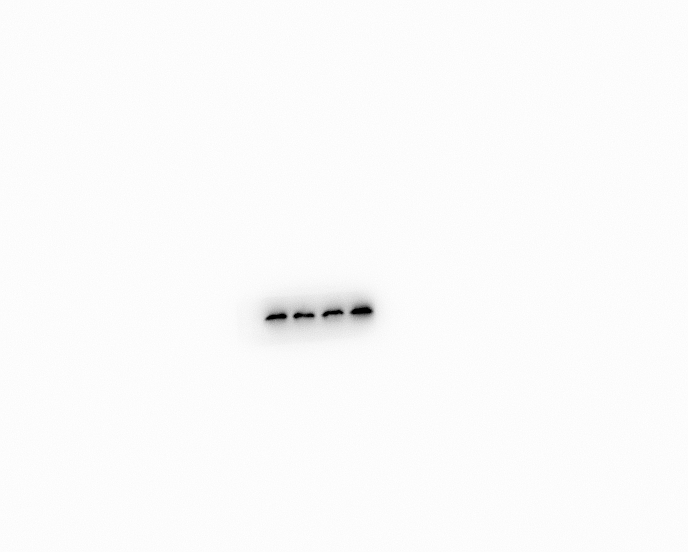

Supplement: Supplementary file 100 — Supplemental Material [file 41419_2022_4955_MOESM100_ESM.tif]

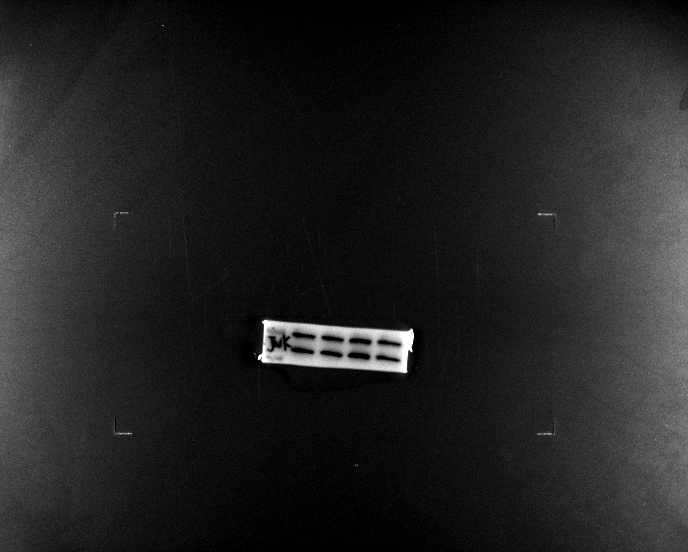

Supplement: Supplementary file 101 — Supplemental Material [file 41419_2022_4955_MOESM101_ESM.tif]

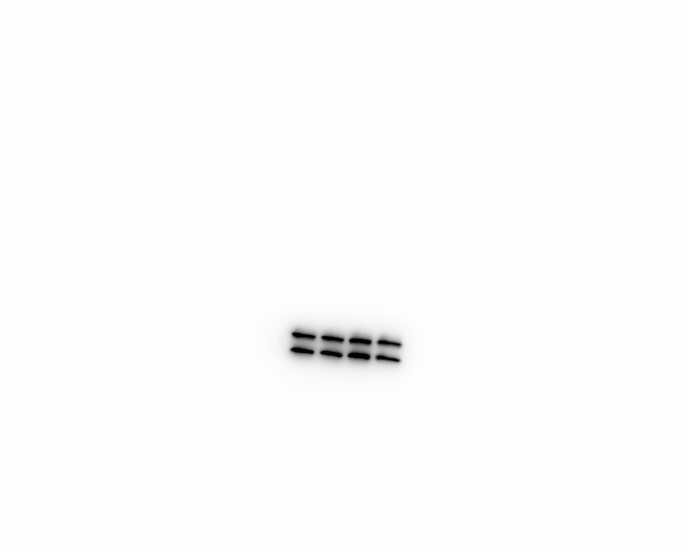

Supplement: Supplementary file 102 — Supplemental Material [file 41419_2022_4955_MOESM102_ESM.tif]

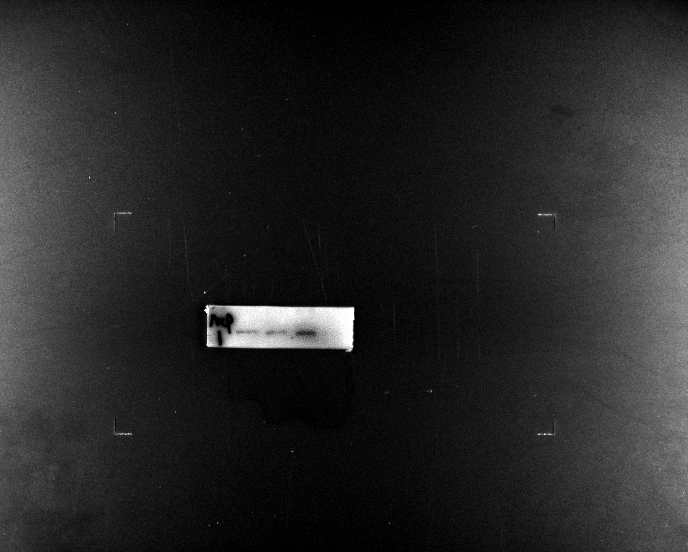

Supplement: Supplementary file 103 — Supplemental Material [file 41419_2022_4955_MOESM103_ESM.tif]
