# Supplementary material for: Antibiotic pretreatment attenuates liver ischemia–reperfusion injury by Farnesoid X receptor activation
Source: Cell Death Dis. 2022 May 21;13(5):484. doi: 10.1038/s41419-022-04955-x (PMC9124217; doi:10.1038/s41419-022-04955-x)
Supplement: Supplementary file 4 — Detailed Attribution of Authorship [file 41419_2022_4955_MOESM4_ESM.pdf]

Author contributions

Name	Contributions
Hanyi Liu[#]	Conducted in vivo experiments<br>(Figure 1, 2, 3, 4, 5A, 5B, 6A, 6B)
Jinglin Wang[#]	Conducted in vitro experiments, collected and analyzed clinical samples<br>(Figure 5C-5J, 6C-6K, 7)
Yitao Ding[*]	Manuscript writing Yitao Ding
Xiaolei Shi[*]	Conceive and critical revisions Xiaolei Shi
Haozhen Ren[*]	Conceive and critical revisions Haozhen Ren
