# Supplementary figures and images for: Antibiotic pretreatment attenuates liver ischemia–reperfusion injury by Farnesoid X receptor activation (part 2 of 2)
Source: Cell Death Dis. 2022 May 21;13(5):484. doi: 10.1038/s41419-022-04955-x (PMC9124217; doi:10.1038/s41419-022-04955-x)

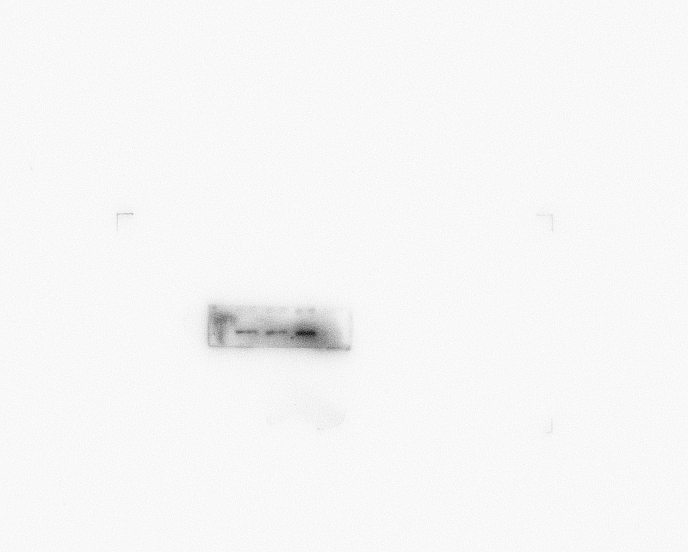

Supplement: Supplementary file 104 — Supplemental Material [file 41419_2022_4955_MOESM104_ESM.tif]

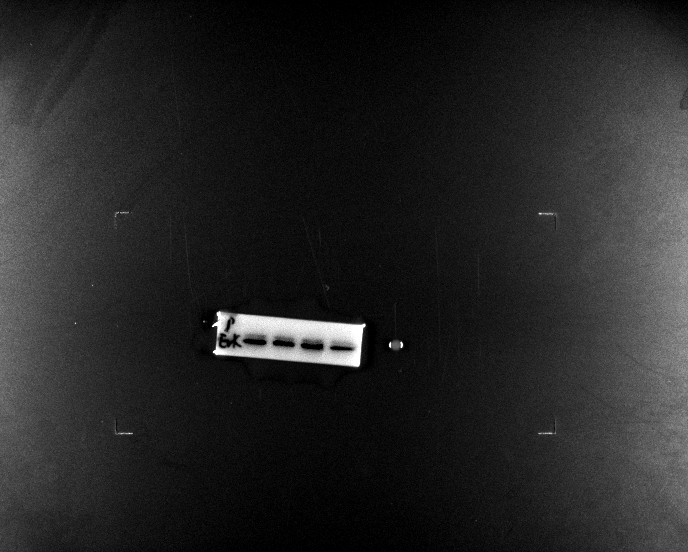

Supplement: Supplementary file 105 — Supplemental Material [file 41419_2022_4955_MOESM105_ESM.tif]

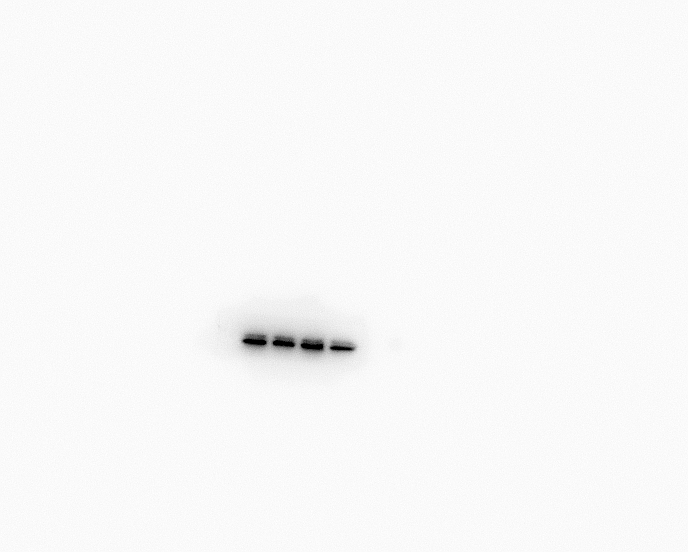

Supplement: Supplementary file 106 — Supplemental Material [file 41419_2022_4955_MOESM106_ESM.tif]

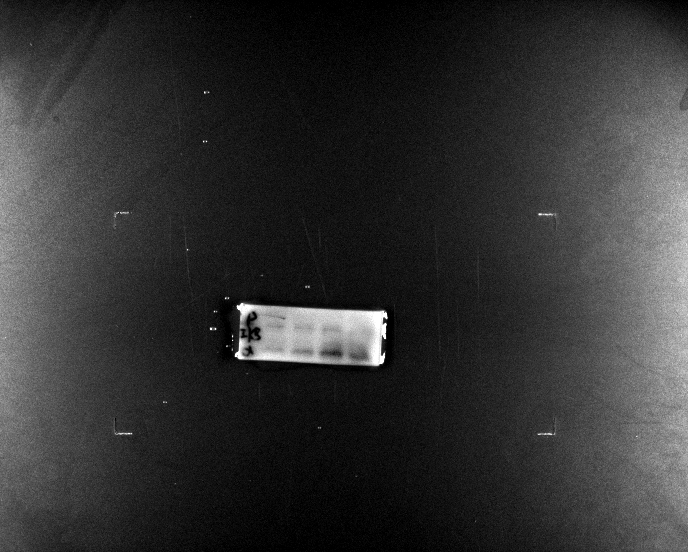

Supplement: Supplementary file 107 — Supplemental Material [file 41419_2022_4955_MOESM107_ESM.tif]

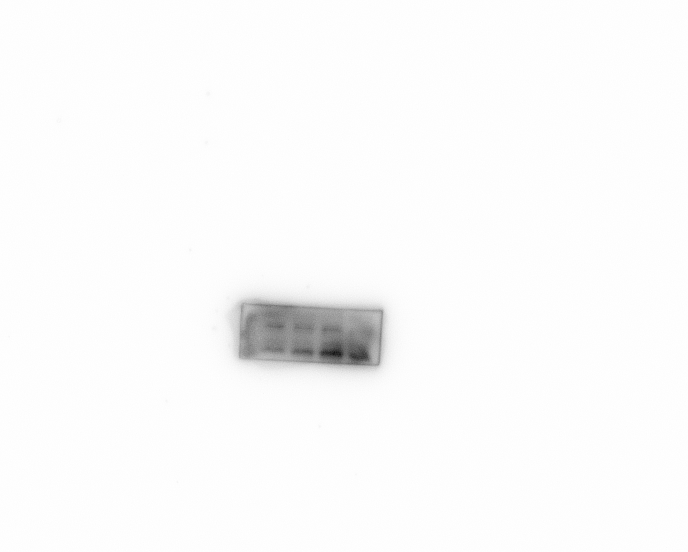

Supplement: Supplementary file 108 — Supplemental Material [file 41419_2022_4955_MOESM108_ESM.tif]

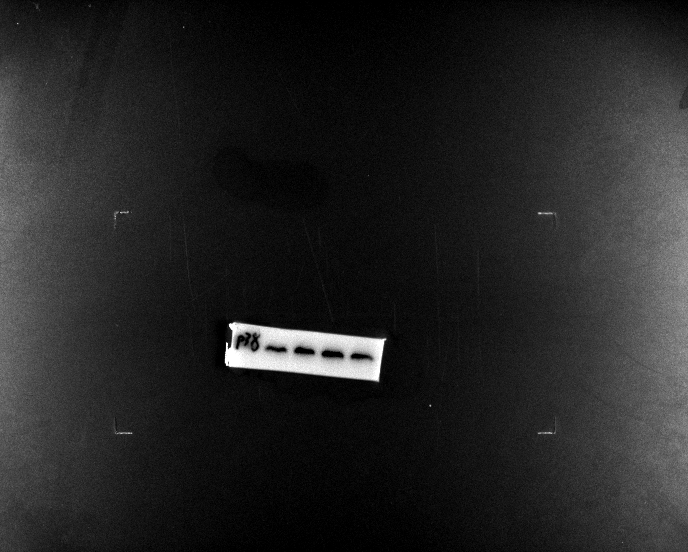

Supplement: Supplementary file 109 — Supplemental Material [file 41419_2022_4955_MOESM109_ESM.tif]

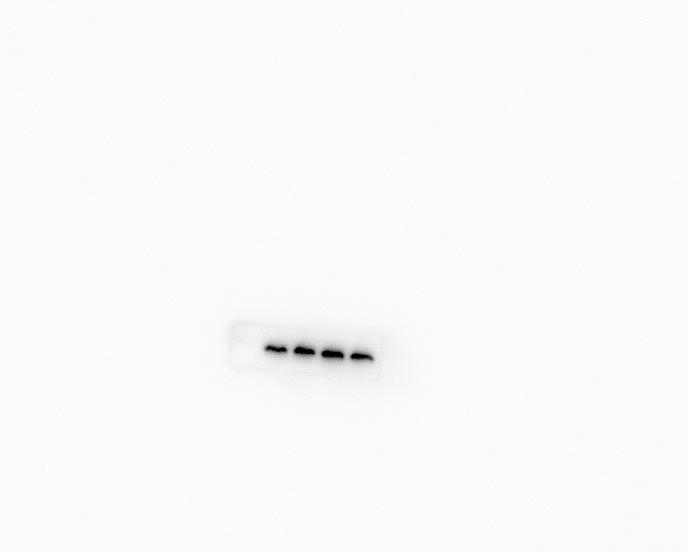

Supplement: Supplementary file 110 — Supplemental Material [file 41419_2022_4955_MOESM110_ESM.tif]

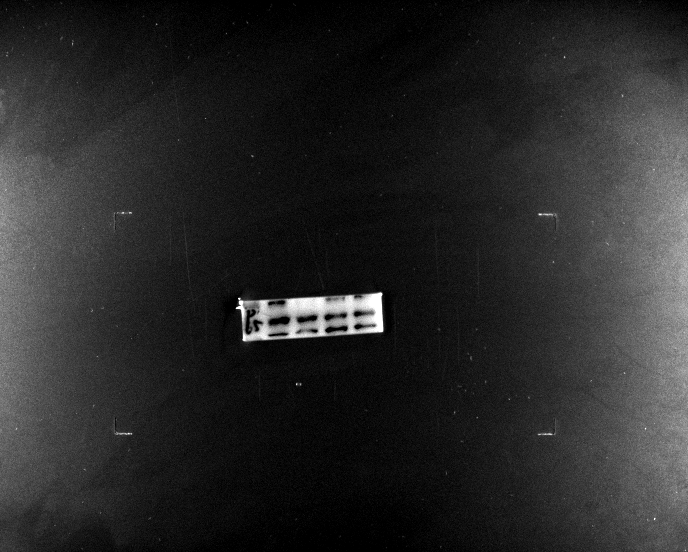

Supplement: Supplementary file 111 — Supplemental Material [file 41419_2022_4955_MOESM111_ESM.tif]

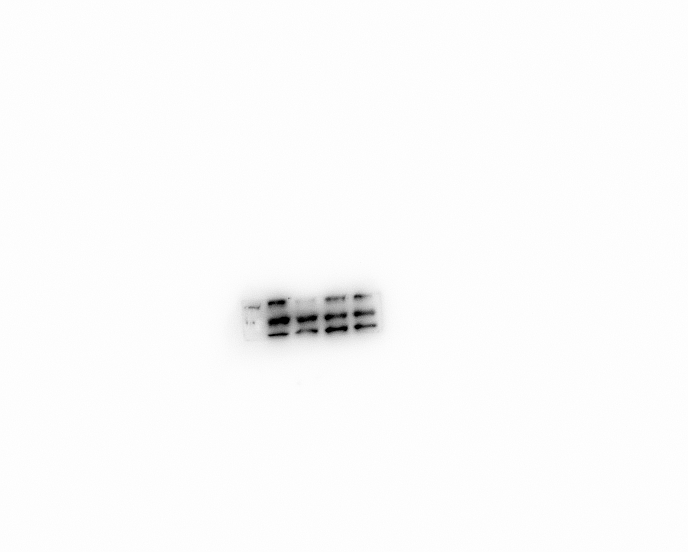

Supplement: Supplementary file 112 — Supplemental Material [file 41419_2022_4955_MOESM112_ESM.tif]

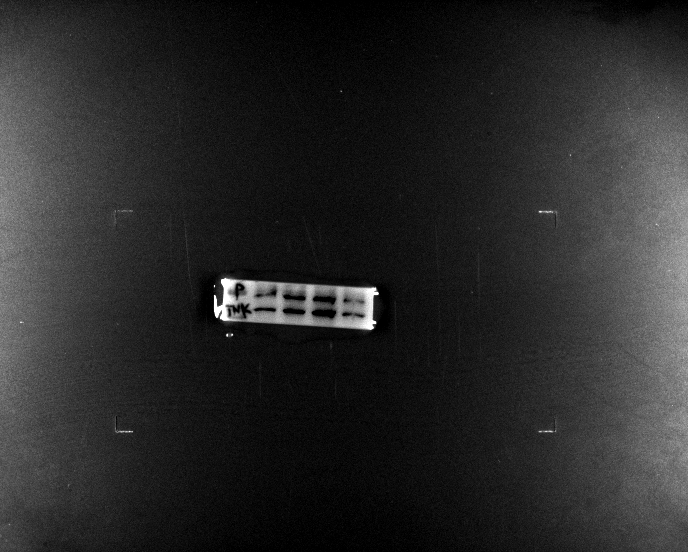

Supplement: Supplementary file 113 — Supplemental Material [file 41419_2022_4955_MOESM113_ESM.tif]

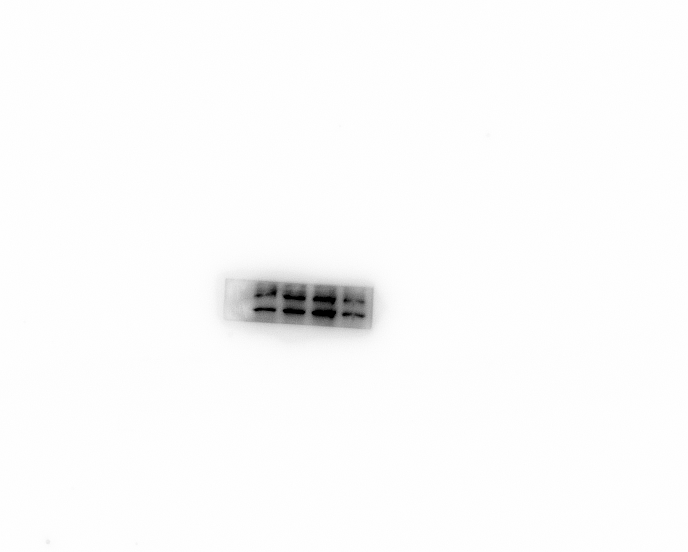

Supplement: Supplementary file 114 — Supplemental Material [file 41419_2022_4955_MOESM114_ESM.tif]

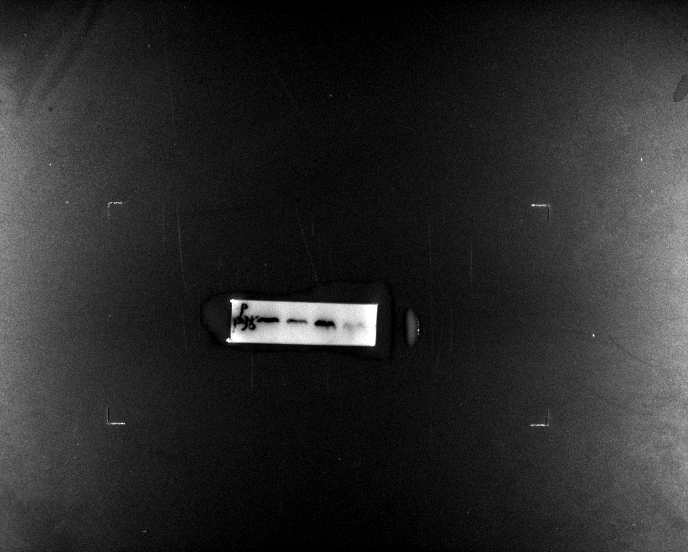

Supplement: Supplementary file 115 — Supplemental Material [file 41419_2022_4955_MOESM115_ESM.tif]

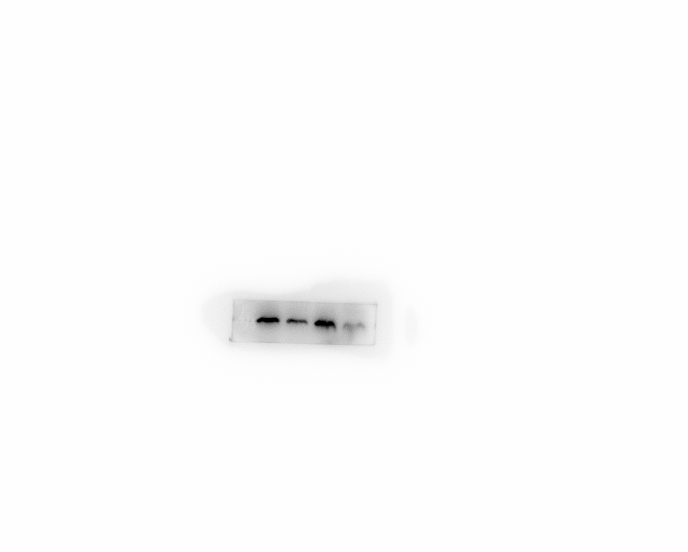

Supplement: Supplementary file 116 — Supplemental Material [file 41419_2022_4955_MOESM116_ESM.tif]

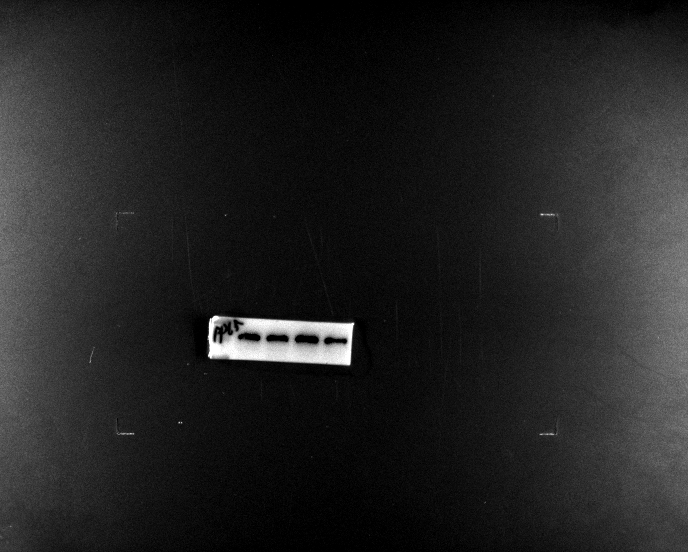

Supplement: Supplementary file 117 — Supplemental Material [file 41419_2022_4955_MOESM117_ESM.tif]

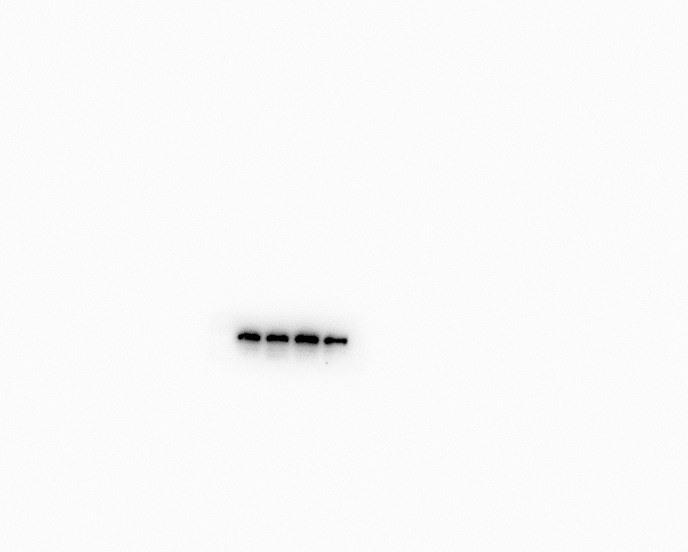

Supplement: Supplementary file 118 — Supplemental Material [file 41419_2022_4955_MOESM118_ESM.tif]

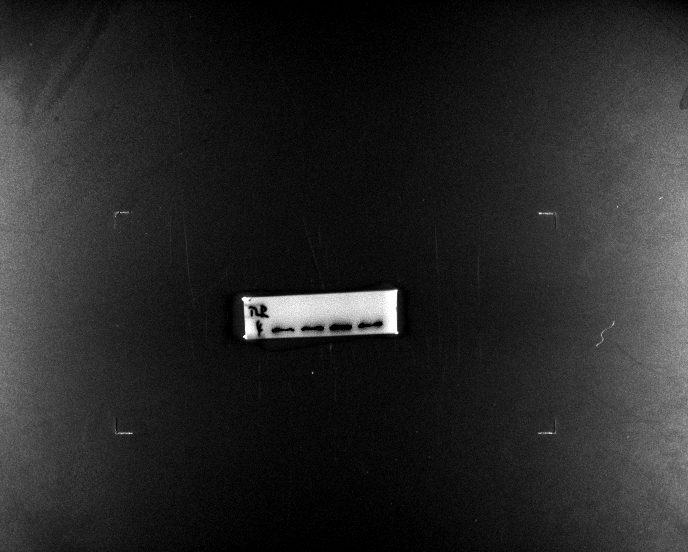

Supplement: Supplementary file 119 — Supplemental Material [file 41419_2022_4955_MOESM119_ESM.tif]

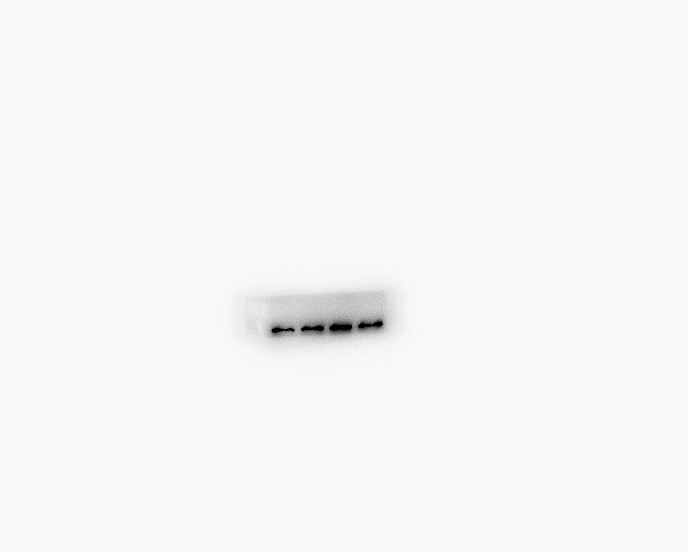

Supplement: Supplementary file 120 — Supplemental Material [file 41419_2022_4955_MOESM120_ESM.tif]

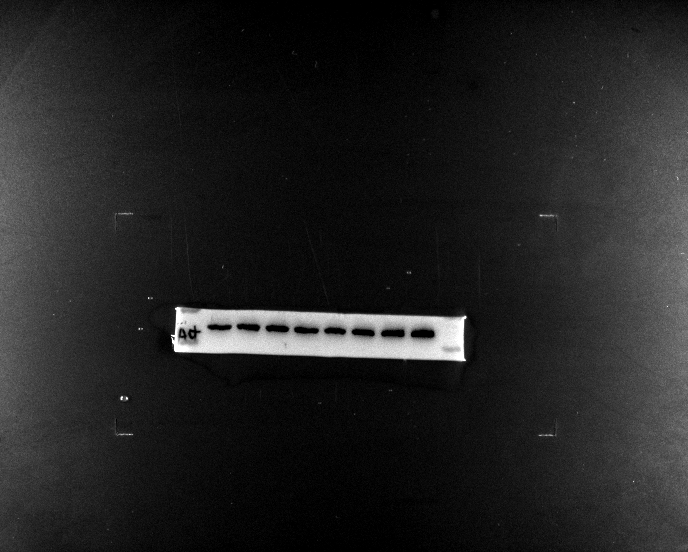

Supplement: Supplementary file 121 — Supplemental Material [file 41419_2022_4955_MOESM121_ESM.tif]

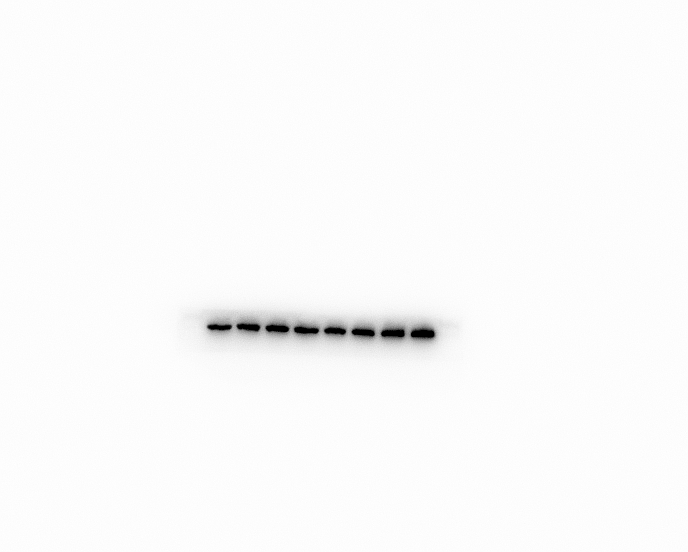

Supplement: Supplementary file 122 — Supplemental Material [file 41419_2022_4955_MOESM122_ESM.tif]

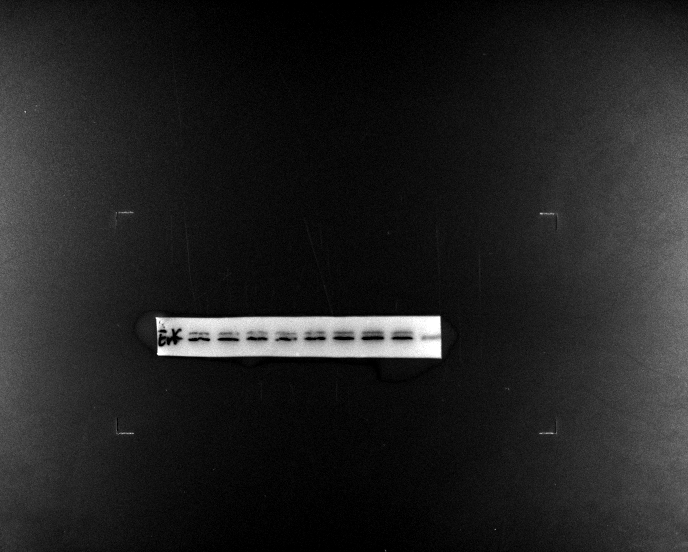

Supplement: Supplementary file 123 — Supplemental Material [file 41419_2022_4955_MOESM123_ESM.tif]

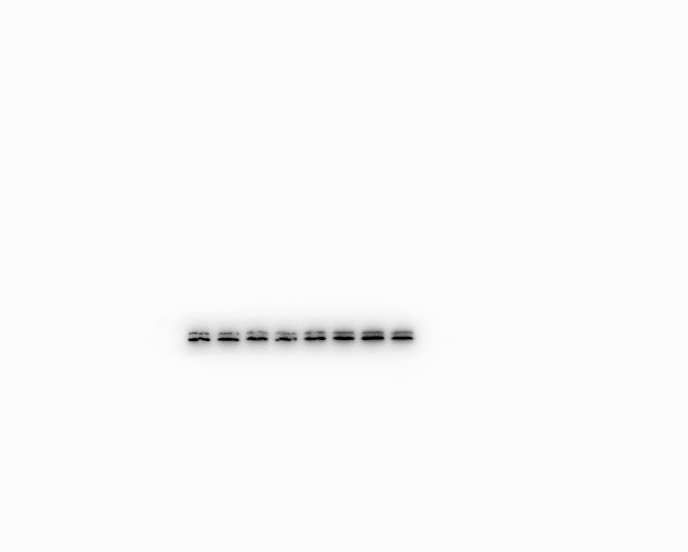

Supplement: Supplementary file 124 — Supplemental Material [file 41419_2022_4955_MOESM124_ESM.tif]

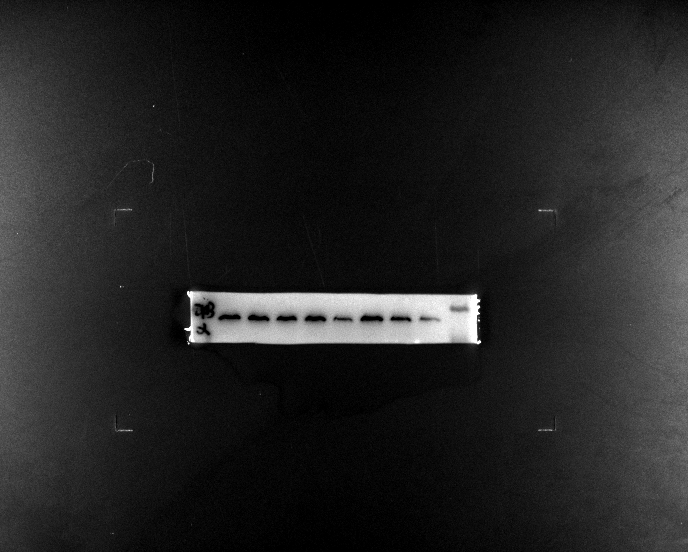

Supplement: Supplementary file 125 — Supplemental Material [file 41419_2022_4955_MOESM125_ESM.tif]

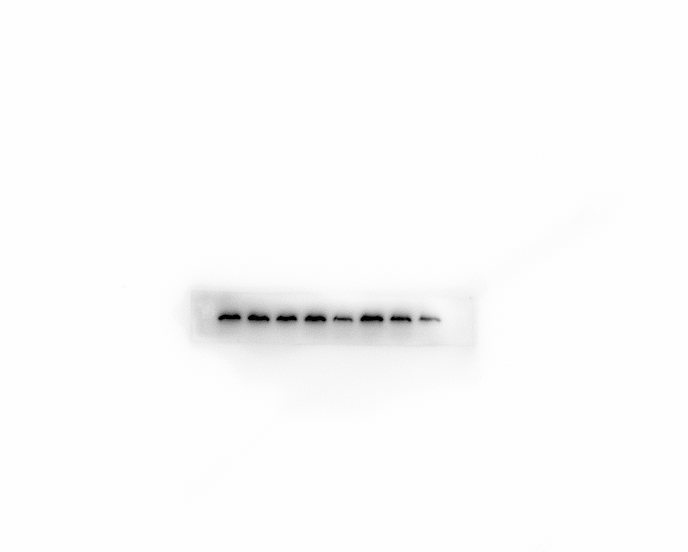

Supplement: Supplementary file 126 — Supplemental Material [file 41419_2022_4955_MOESM126_ESM.tif]

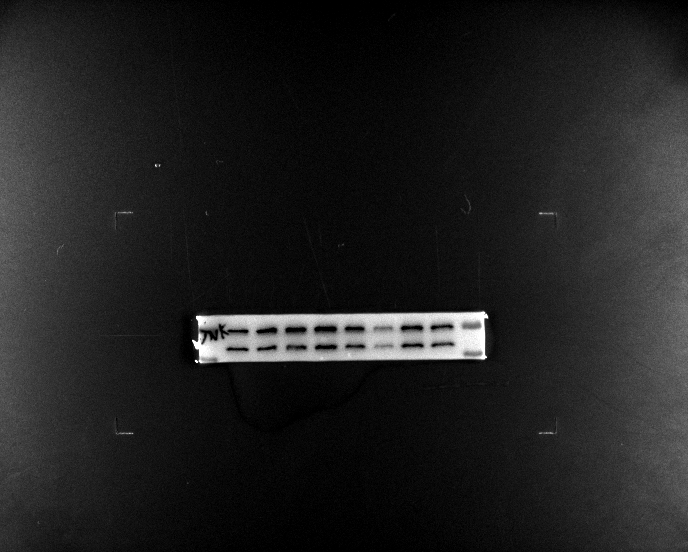

Supplement: Supplementary file 127 — Supplemental Material [file 41419_2022_4955_MOESM127_ESM.tif]

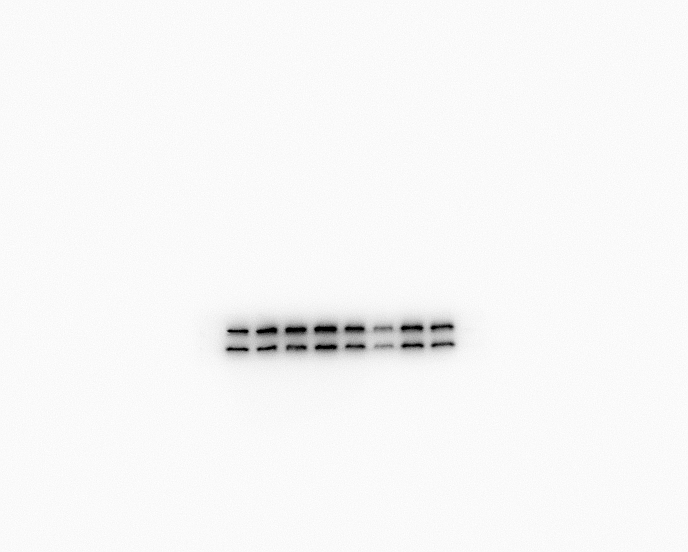

Supplement: Supplementary file 128 — Supplemental Material [file 41419_2022_4955_MOESM128_ESM.tif]

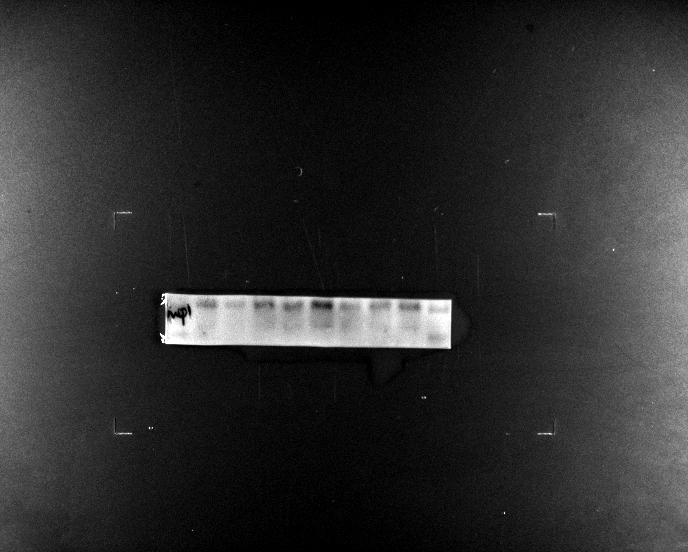

Supplement: Supplementary file 129 — Supplemental Material [file 41419_2022_4955_MOESM129_ESM.tif]

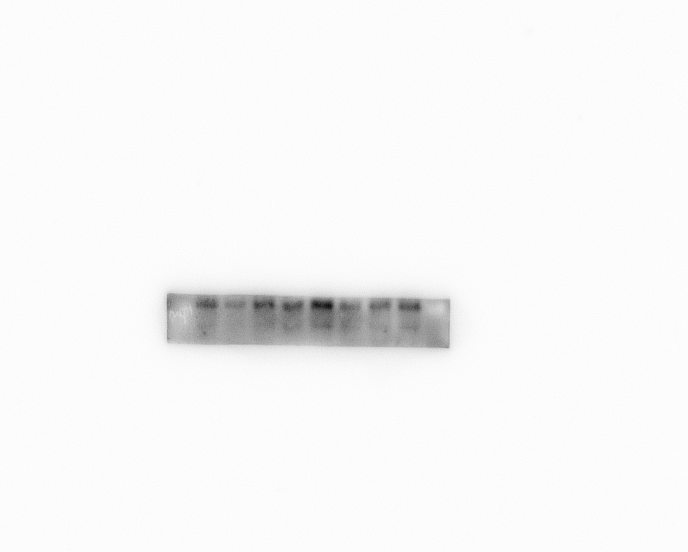

Supplement: Supplementary file 130 — Supplemental Material [file 41419_2022_4955_MOESM130_ESM.tif]

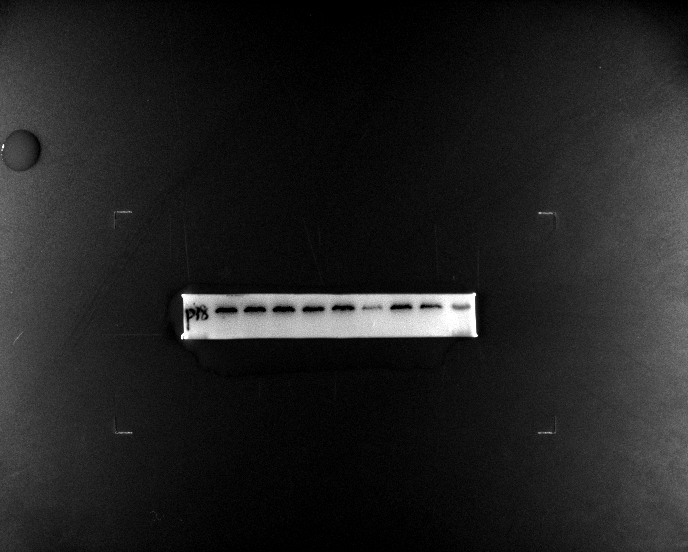

Supplement: Supplementary file 131 — Supplemental Material [file 41419_2022_4955_MOESM131_ESM.tif]

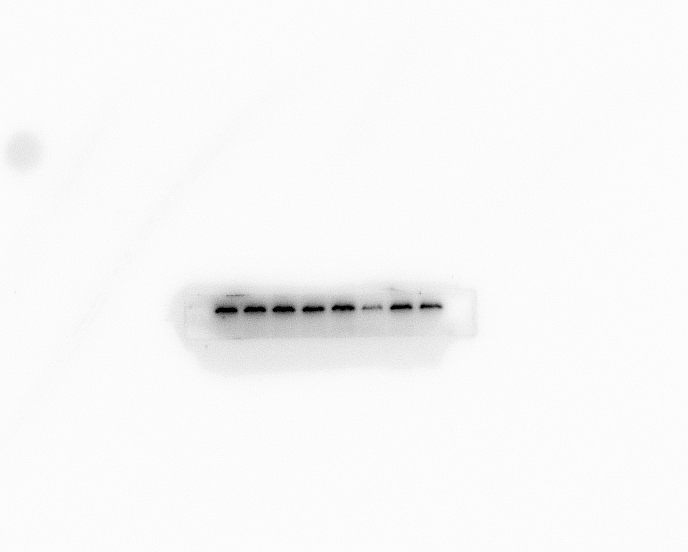

Supplement: Supplementary file 132 — Supplemental Material [file 41419_2022_4955_MOESM132_ESM.tif]

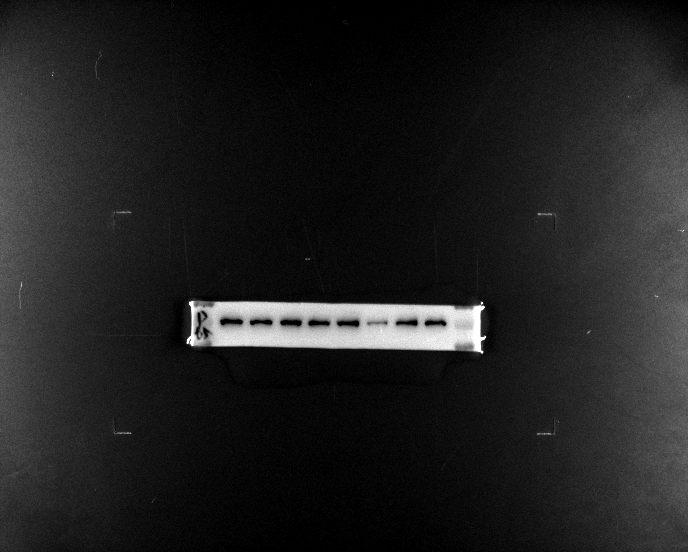

Supplement: Supplementary file 133 — Supplemental Material [file 41419_2022_4955_MOESM133_ESM.tif]

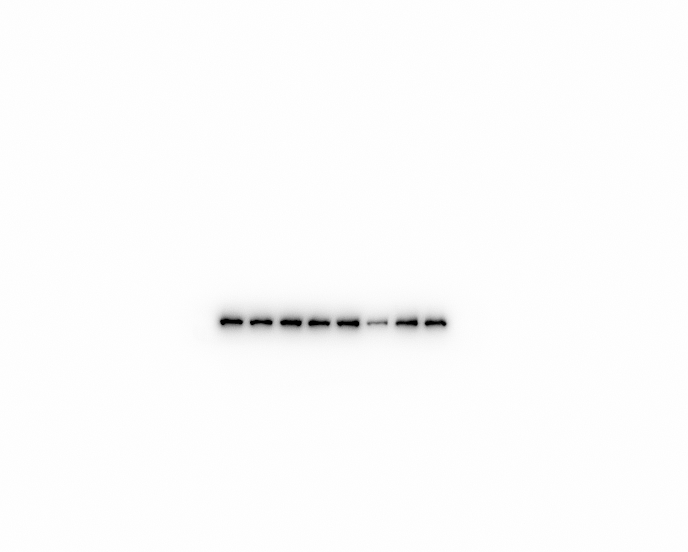

Supplement: Supplementary file 134 — Supplemental Material [file 41419_2022_4955_MOESM134_ESM.tif]

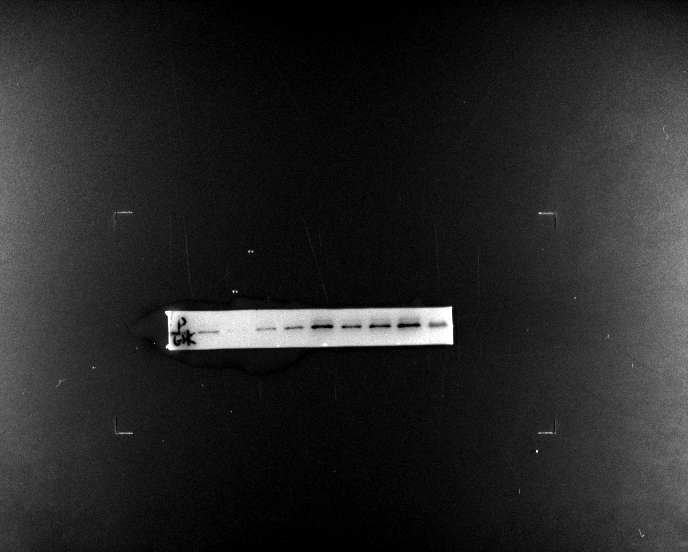

Supplement: Supplementary file 135 — Supplemental Material [file 41419_2022_4955_MOESM135_ESM.tif]

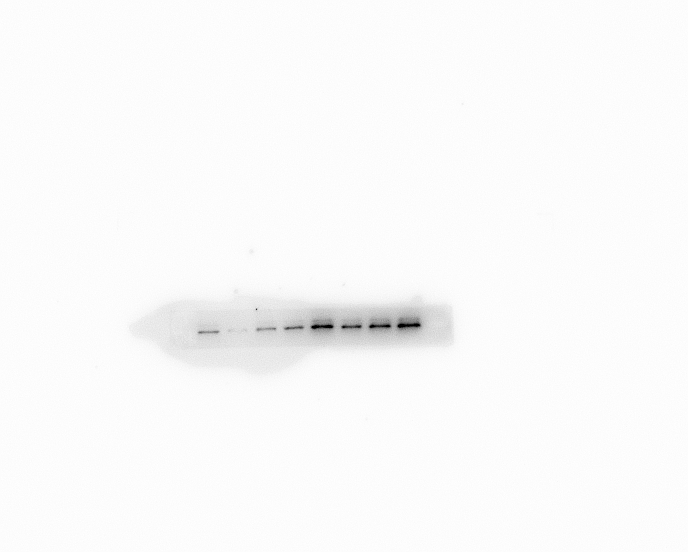

Supplement: Supplementary file 136 — Supplemental Material [file 41419_2022_4955_MOESM136_ESM.tif]

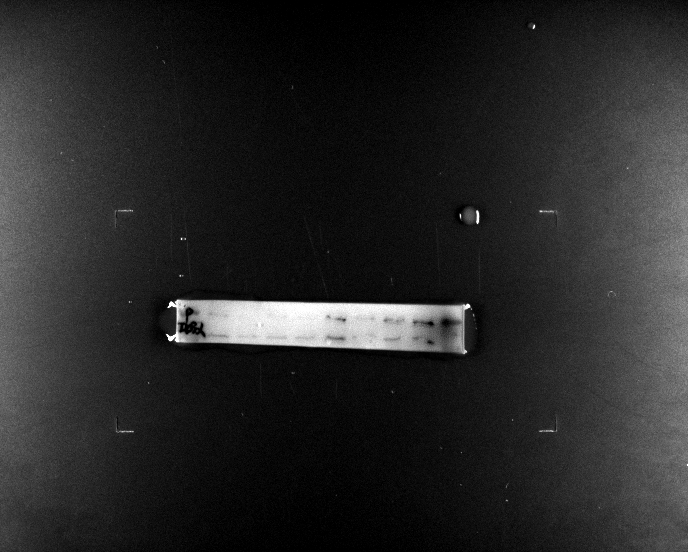

Supplement: Supplementary file 137 — Supplemental Material [file 41419_2022_4955_MOESM137_ESM.tif]

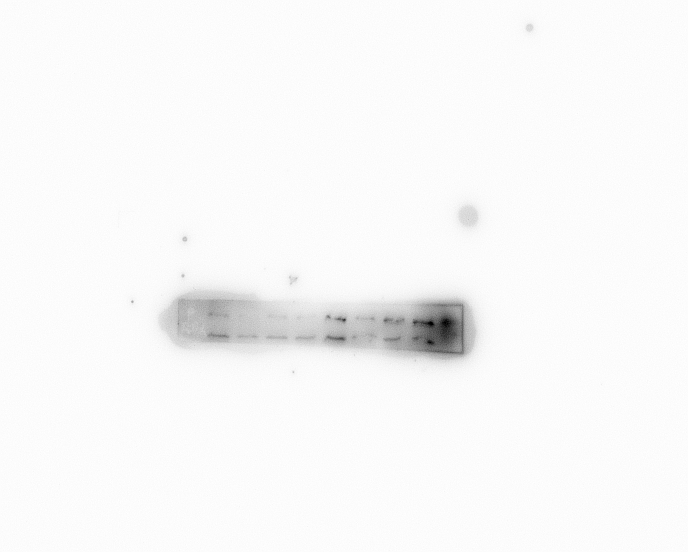

Supplement: Supplementary file 138 — Supplemental Material [file 41419_2022_4955_MOESM138_ESM.tif]

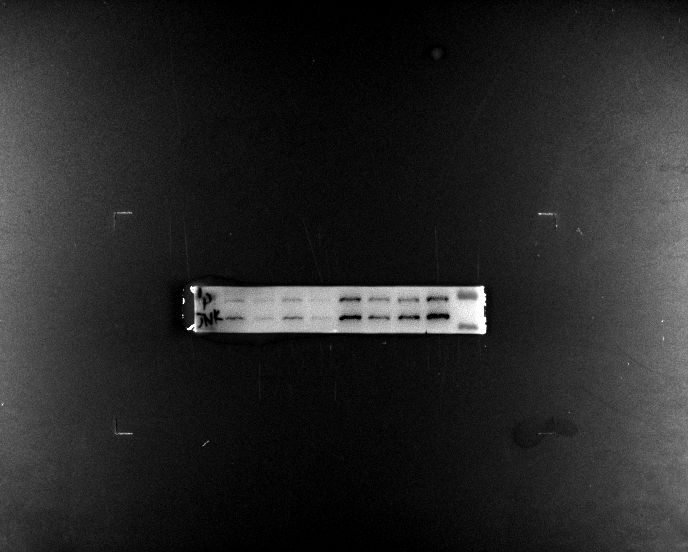

Supplement: Supplementary file 139 — Supplemental Material [file 41419_2022_4955_MOESM139_ESM.tif]

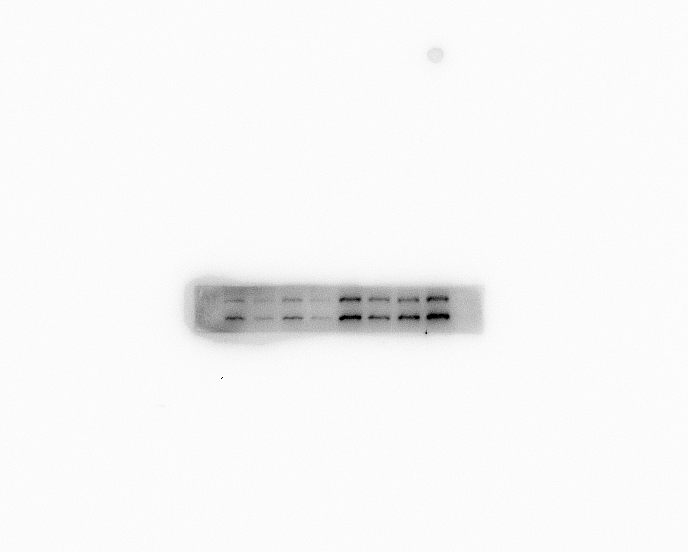

Supplement: Supplementary file 140 — Supplemental Material [file 41419_2022_4955_MOESM140_ESM.tif]

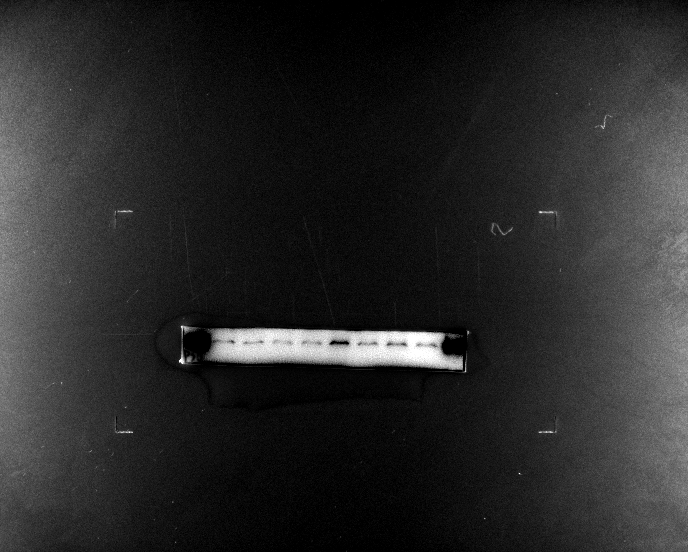

Supplement: Supplementary file 141 — Supplemental Material [file 41419_2022_4955_MOESM141_ESM.tif]

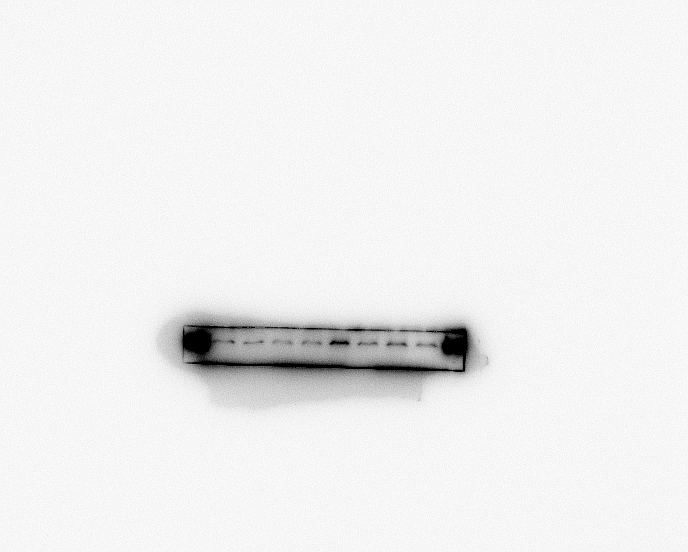

Supplement: Supplementary file 142 — Supplemental Material [file 41419_2022_4955_MOESM142_ESM.tif]

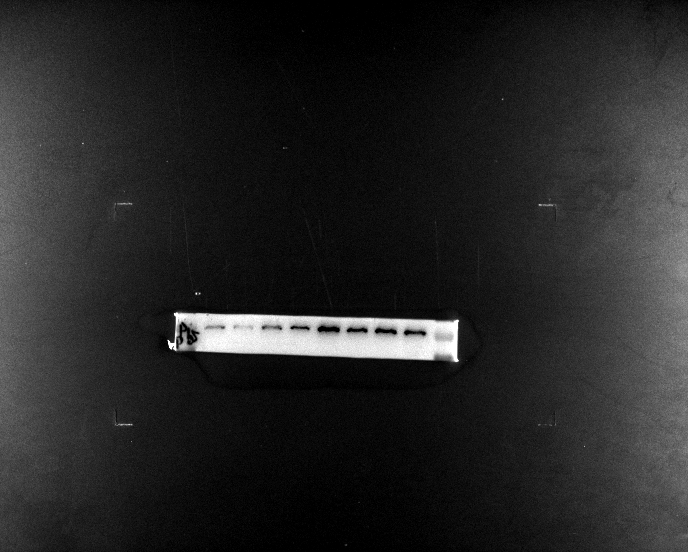

Supplement: Supplementary file 143 — Supplemental Material [file 41419_2022_4955_MOESM143_ESM.tif]

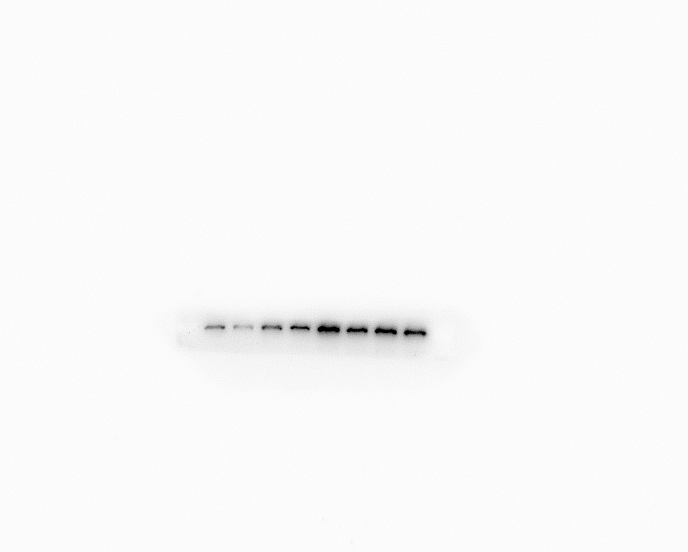

Supplement: Supplementary file 144 — Supplemental Material [file 41419_2022_4955_MOESM144_ESM.tif]

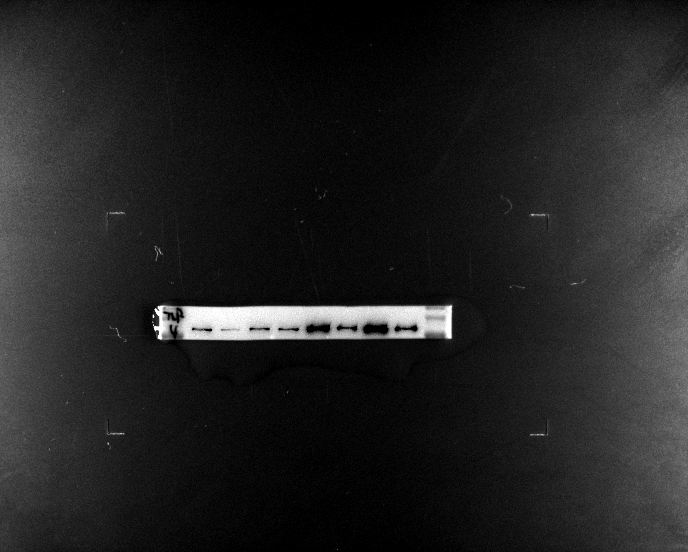

Supplement: Supplementary file 145 — Supplemental Material [file 41419_2022_4955_MOESM145_ESM.tif]

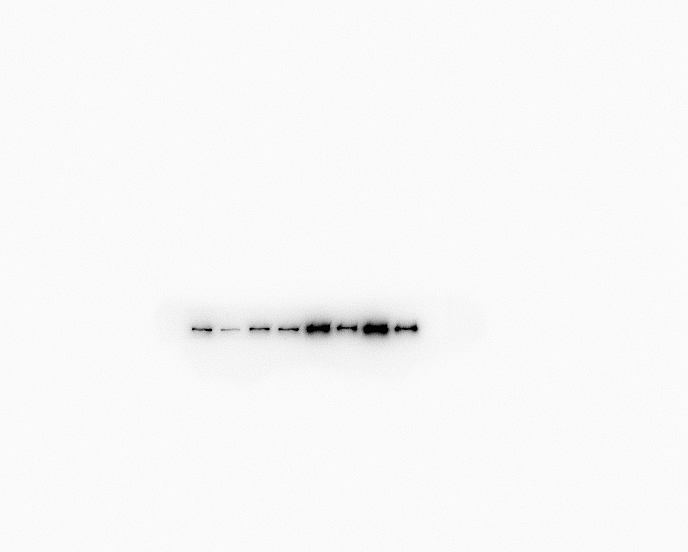

Supplement: Supplementary file 146 — Supplemental Material [file 41419_2022_4955_MOESM146_ESM.tif]

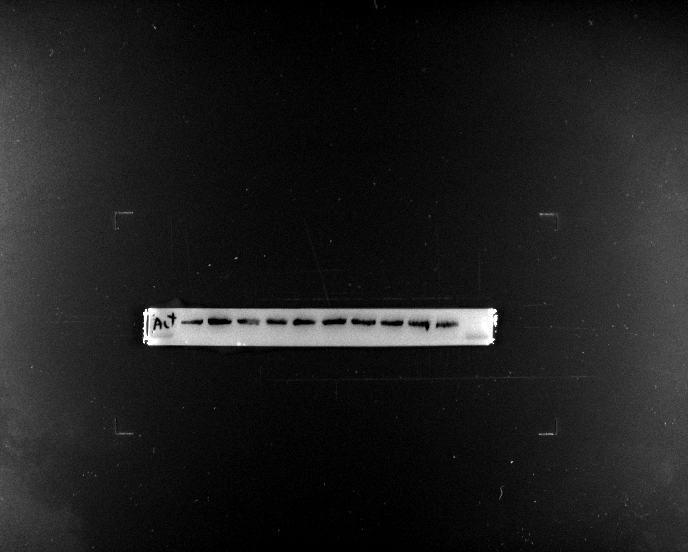

Supplement: Supplementary file 147 — Supplemental Material [file 41419_2022_4955_MOESM147_ESM.tif]

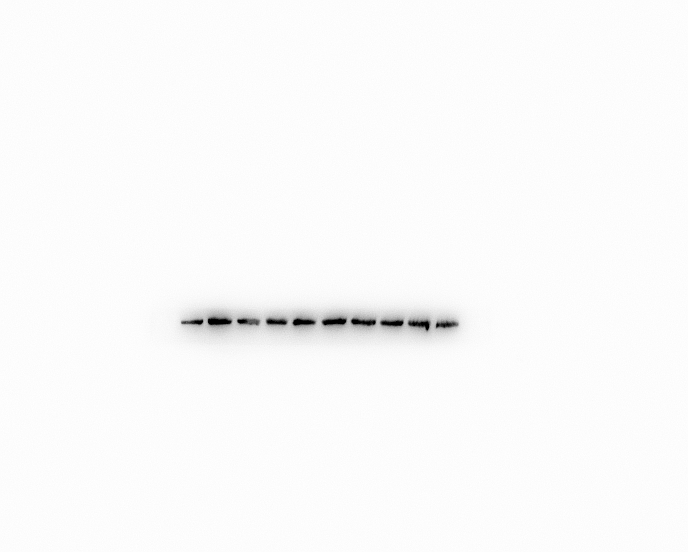

Supplement: Supplementary file 148 — Supplemental Material [file 41419_2022_4955_MOESM148_ESM.tif]

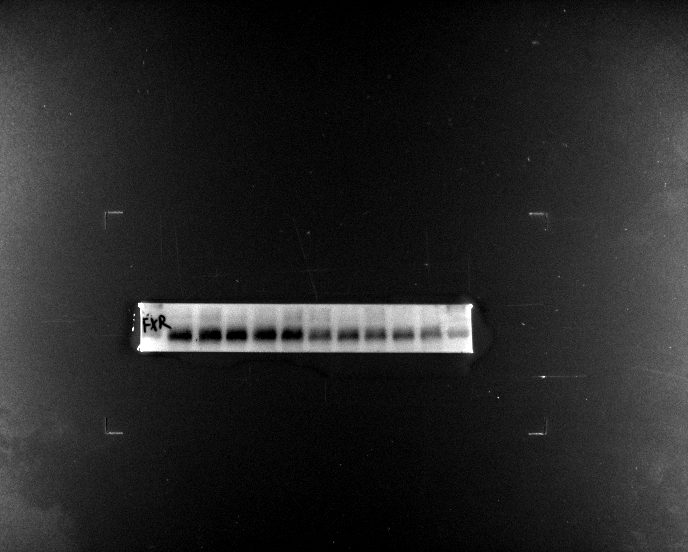

Supplement: Supplementary file 149 — Supplemental Material [file 41419_2022_4955_MOESM149_ESM.tif]

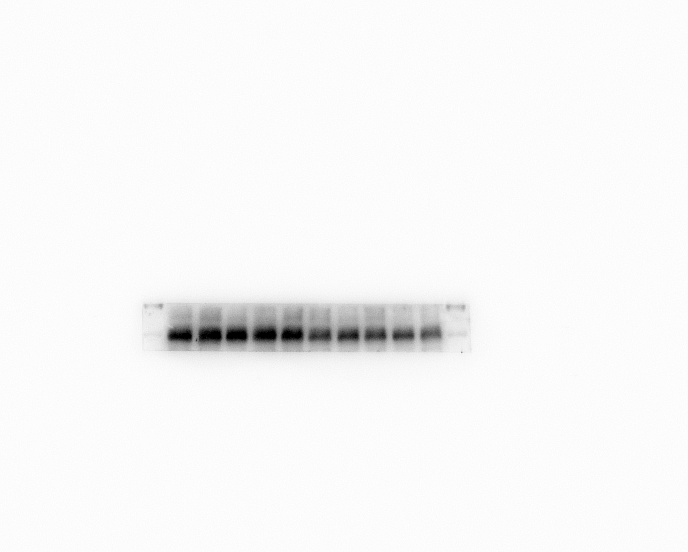

Supplement: Supplementary file 150 — Supplemental Material [file 41419_2022_4955_MOESM150_ESM.tif]

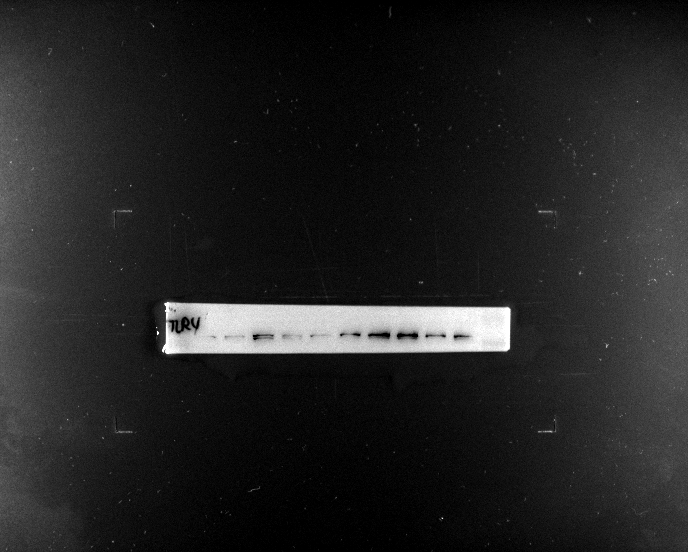

Supplement: Supplementary file 151 — Supplemental Material [file 41419_2022_4955_MOESM151_ESM.tif]

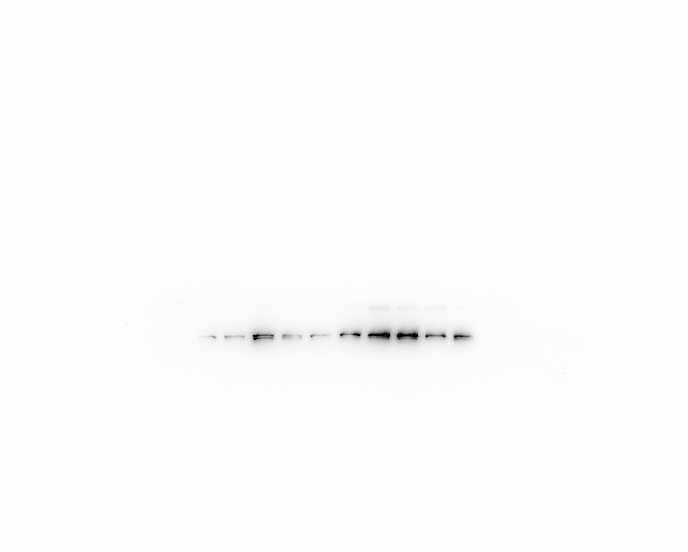

Supplement: Supplementary file 152 — Supplemental Material [file 41419_2022_4955_MOESM152_ESM.tif]

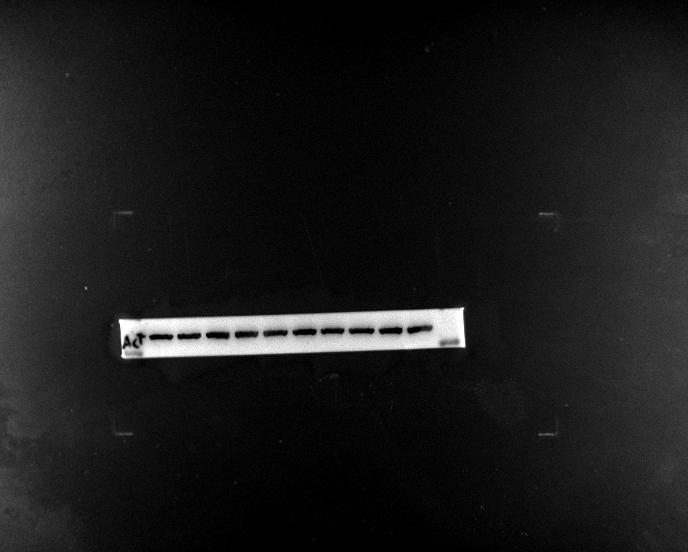

Supplement: Supplementary file 153 — Supplemental Material [file 41419_2022_4955_MOESM153_ESM.tif]

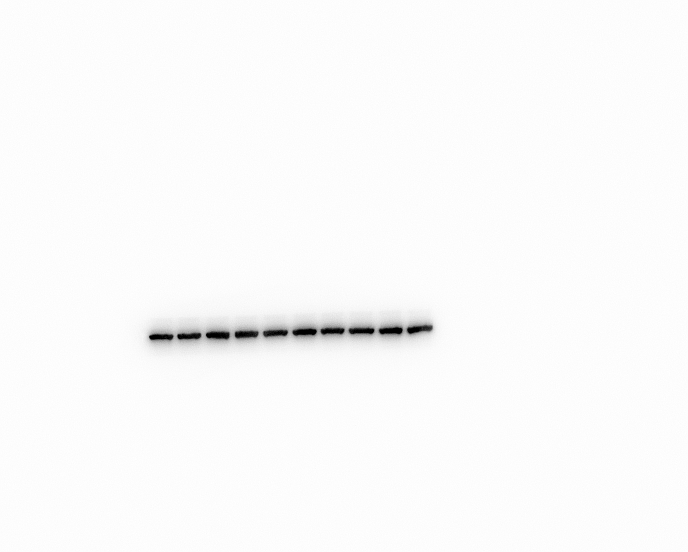

Supplement: Supplementary file 154 — Supplemental Material [file 41419_2022_4955_MOESM154_ESM.tif]

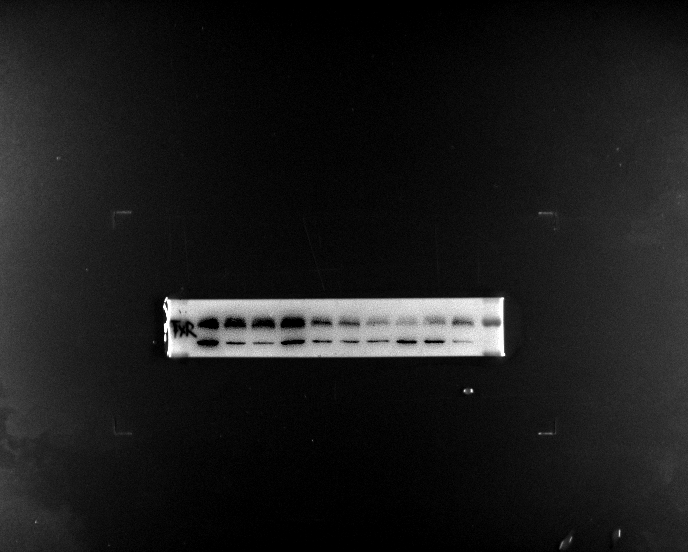

Supplement: Supplementary file 155 — Supplemental Material [file 41419_2022_4955_MOESM155_ESM.tif]

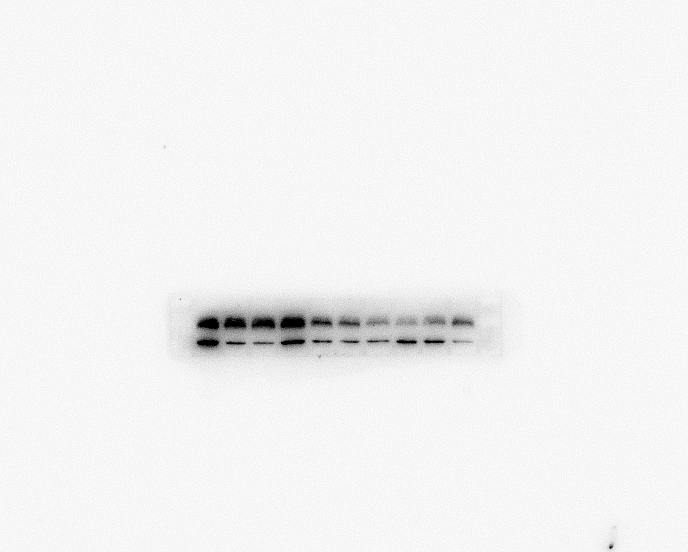

Supplement: Supplementary file 156 — Supplemental Material [file 41419_2022_4955_MOESM156_ESM.tif]

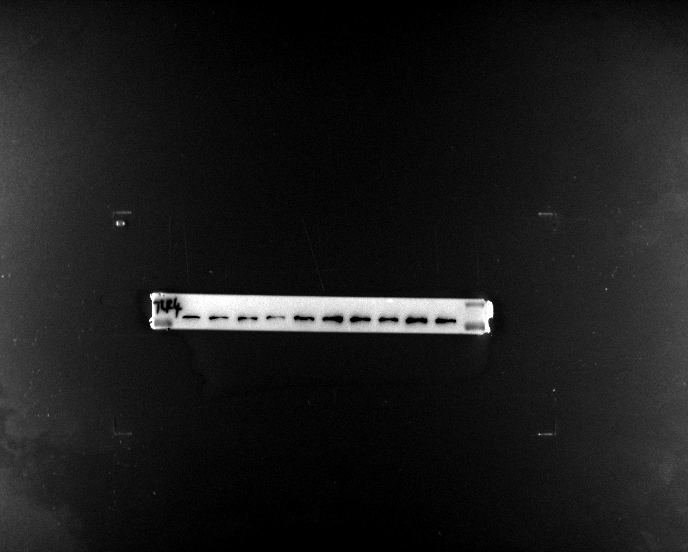

Supplement: Supplementary file 157 — Supplemental Material [file 41419_2022_4955_MOESM157_ESM.tif]

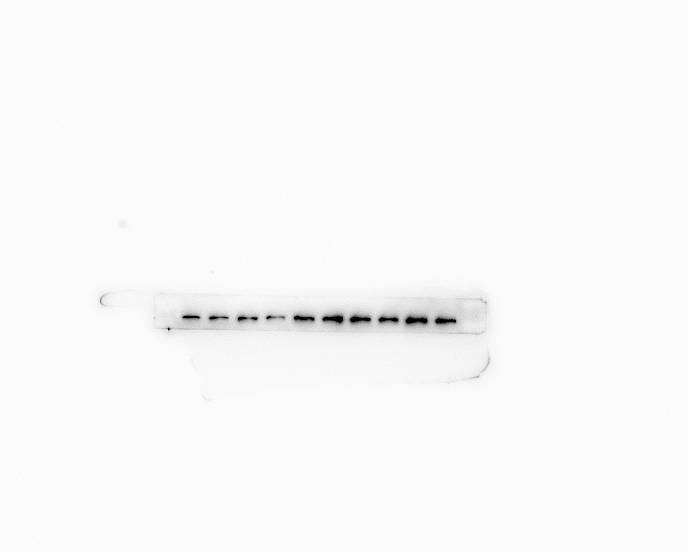

Supplement: Supplementary file 158 — Supplemental Material [file 41419_2022_4955_MOESM158_ESM.tif]

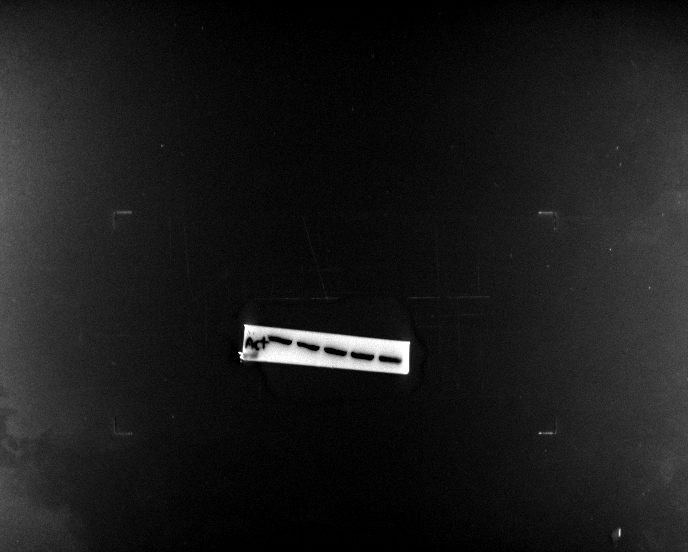

Supplement: Supplementary file 159 — Supplemental Material [file 41419_2022_4955_MOESM159_ESM.tif]

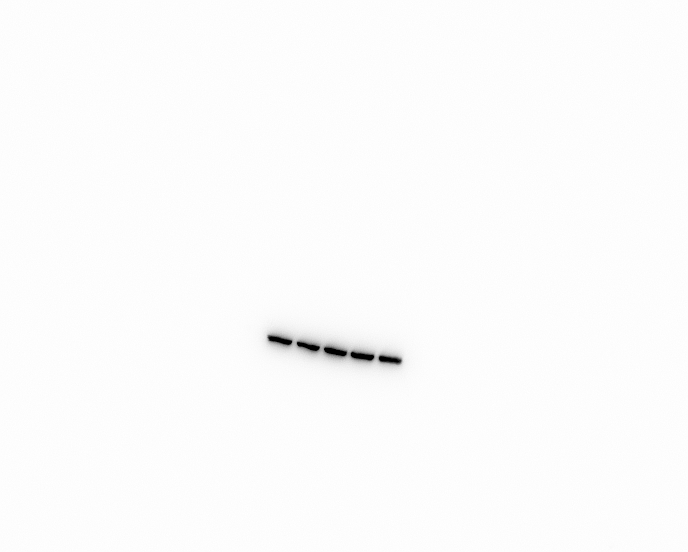

Supplement: Supplementary file 160 — Supplemental Material [file 41419_2022_4955_MOESM160_ESM.tif]

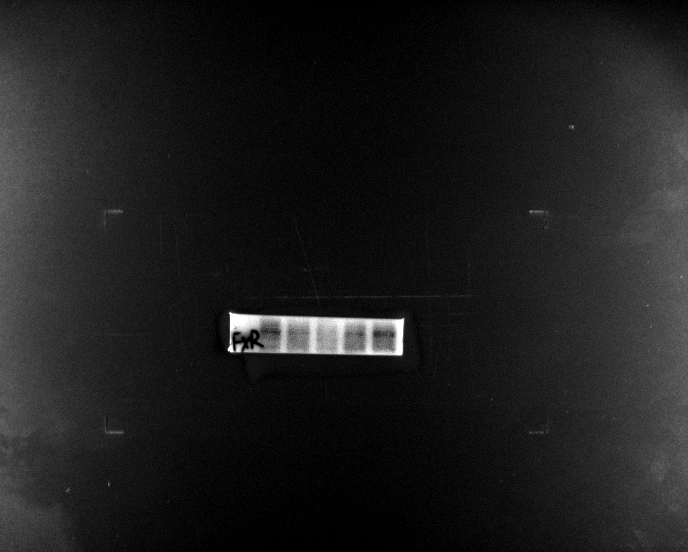

Supplement: Supplementary file 161 — Supplemental Material [file 41419_2022_4955_MOESM161_ESM.tif]

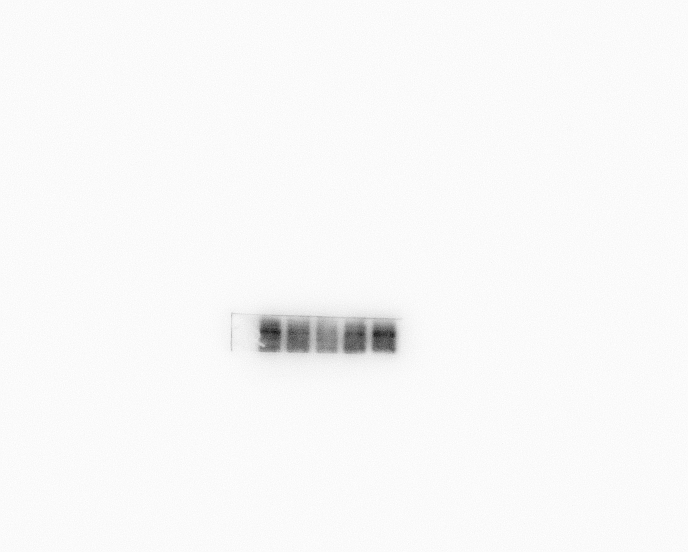

Supplement: Supplementary file 162 — Supplemental Material [file 41419_2022_4955_MOESM162_ESM.tif]

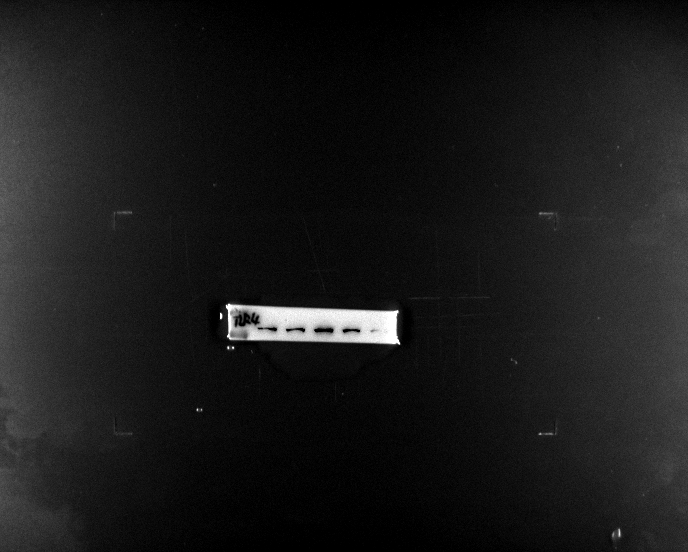

Supplement: Supplementary file 163 — Supplemental Material [file 41419_2022_4955_MOESM163_ESM.tif]

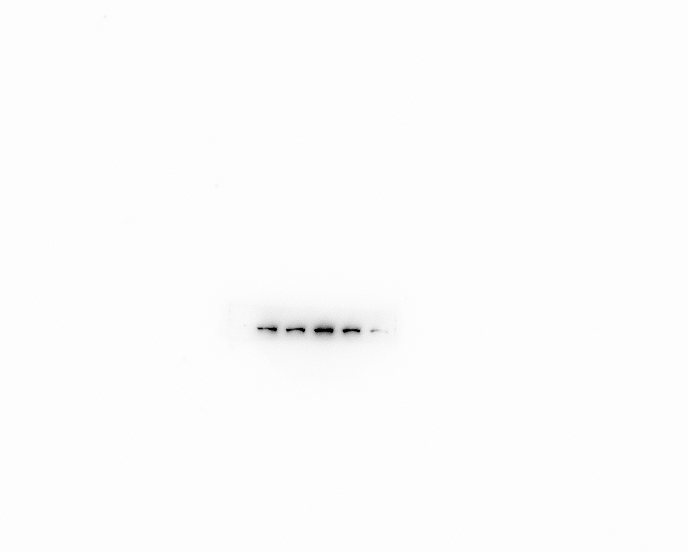

Supplement: Supplementary file 164 — Supplemental Material [file 41419_2022_4955_MOESM164_ESM.tif]

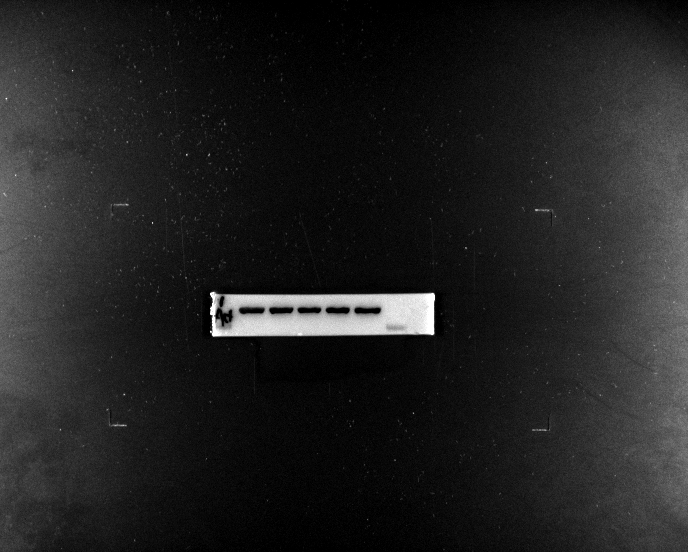

Supplement: Supplementary file 165 — Supplemental Material [file 41419_2022_4955_MOESM165_ESM.tif]

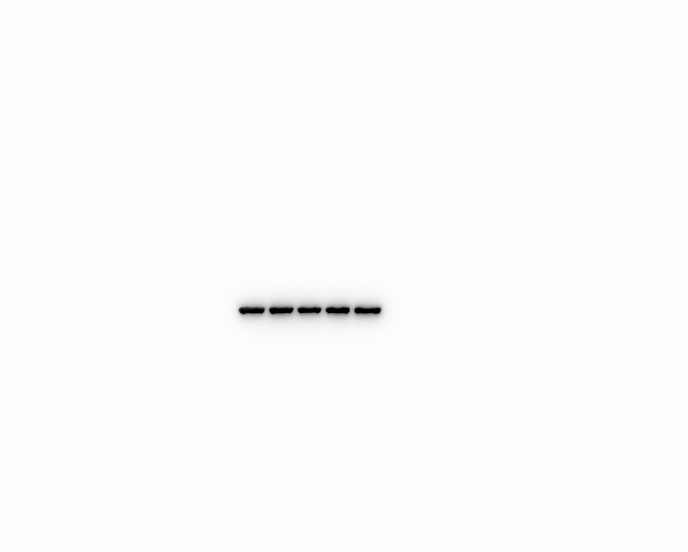

Supplement: Supplementary file 166 — Supplemental Material [file 41419_2022_4955_MOESM166_ESM.tif]

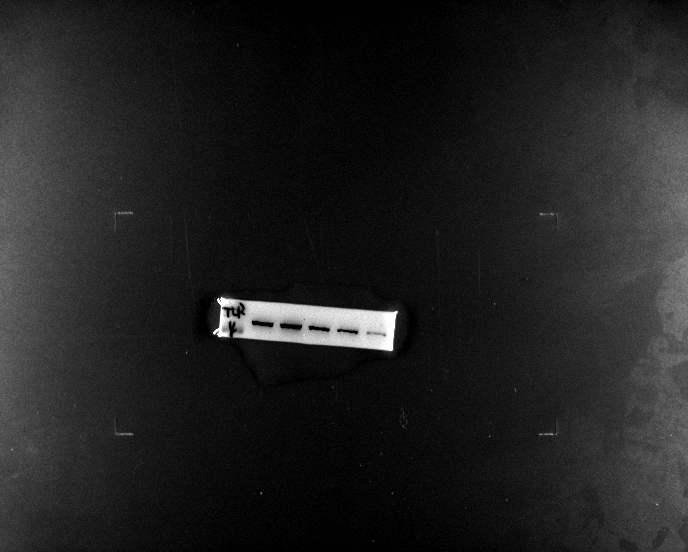

Supplement: Supplementary file 167 — Supplemental Material [file 41419_2022_4955_MOESM167_ESM.tif]

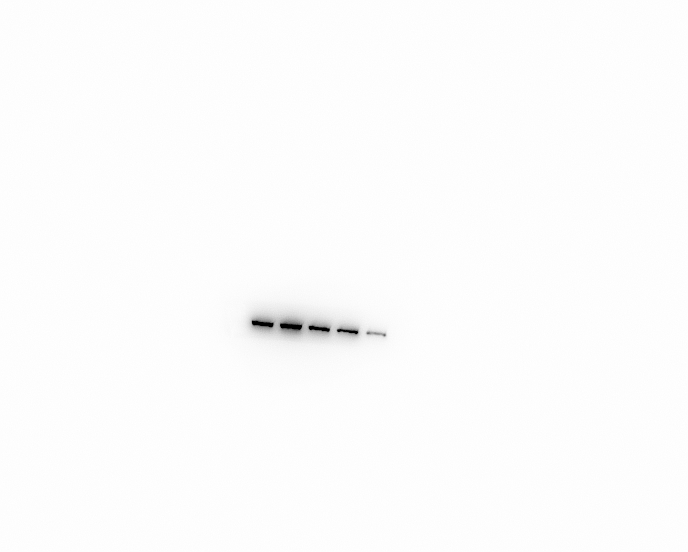

Supplement: Supplementary file 168 — Supplemental Material [file 41419_2022_4955_MOESM168_ESM.tif]

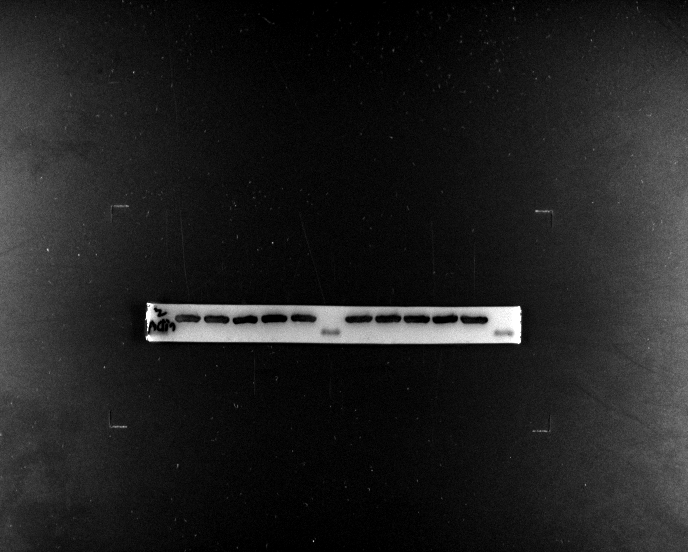

Supplement: Supplementary file 169 — Supplemental Material [file 41419_2022_4955_MOESM169_ESM.tif]

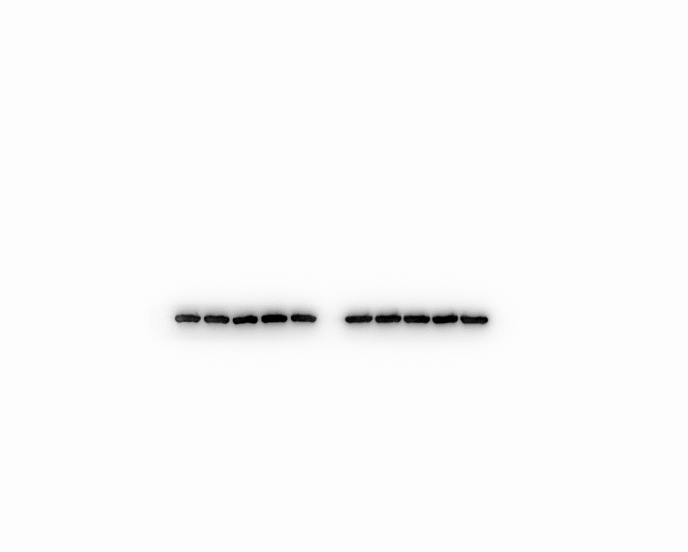

Supplement: Supplementary file 170 — Supplemental Material [file 41419_2022_4955_MOESM170_ESM.tif]

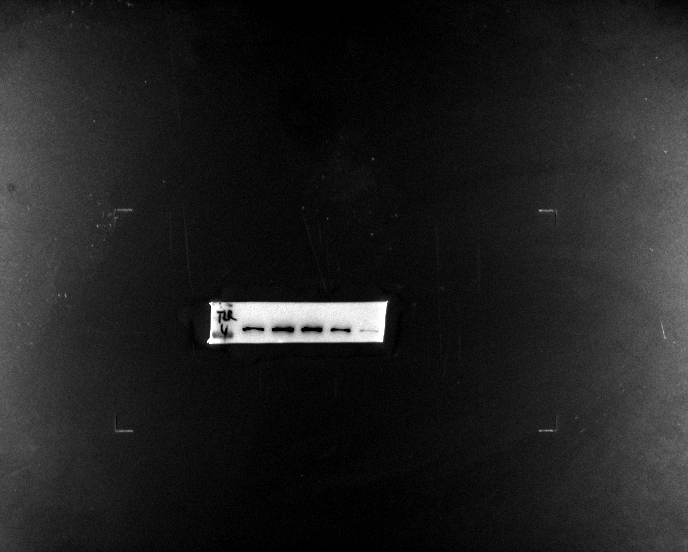

Supplement: Supplementary file 171 — Supplemental Material [file 41419_2022_4955_MOESM171_ESM.tif]

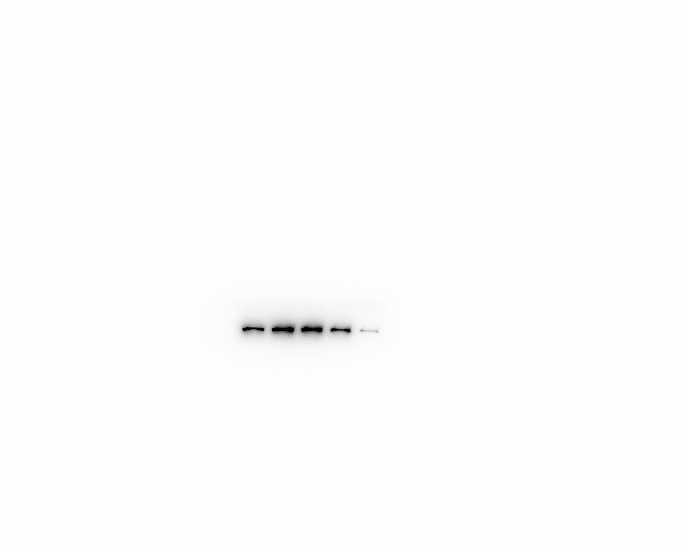

Supplement: Supplementary file 172 — Supplemental Material [file 41419_2022_4955_MOESM172_ESM.tif]
