# Supplementary material for: Antibiotic pretreatment attenuates liver ischemia–reperfusion injury by Farnesoid X receptor activation
Source: Cell Death Dis. 2022 May 21;13(5):484. doi: 10.1038/s41419-022-04955-x (PMC9124217; doi:10.1038/s41419-022-04955-x)
Supplement: Supplementary file 173 — Antibiotic pretreatment attenuates liver ischemia–reperfusion injury by Farnesoid X receptor activation [file 41419_2022_4955_MOESM173_ESM.docx]

**SUPPLEMENTARY MATERIALS**

**TITLE**

Antibiotic pretreatment attenuates liver ischemia-reperfusion injury by Farnesoid X receptor activation

Hanyi Liu^1,#^, Haozhen Ren^1,2, #^, Yitao Ding^1,2,*^, Xiaolei Shi^1,2,*^, Jinglin Wang^1,2, *^

^1^Department of Hepatobiliary Surgery, The Affiliated Drum Tower Hospital of Nanjing University Medical School, Nanjing, China.

^2^Institute of Hepatobiliary Surgery, Nanjing University.

^#^These authors contributed equally to this article.

***Correspondence**

Yitao Ding, Xiaolei Shi and Jinglin Wang, Department of Hepatobiliary Surgery, The Affiliated Drum Tower Hospital of Nanjing University Medical School, 321 Zhongshan Road, Nanjing, Jiangsu 210008, P.R. China.

E‑mail: dytnanjing1983@126.com, [sxl@nju.edu.cn](mailto:sxl@nju.edu.cn) and cw20120817@163.com

**Table S1. Sequence of primers used in experiments**

| Gene | Forward | Reverse |
| --- | --- | --- |
| actin | CATCCGTAAAGACCTCTATGCCAAC | ATGGAGCCACCGATCCACA |
| Il6 | TACCACTTCACAAGTCGGAGGC | CTGCAAGTGCATCATCGTTGTTC |
| Tnfα | GGTGCCTATGTCTCAGCCTCTT | GCCATAGAACTGATGAGAGGGAG |
| Il1β | TGGACCTTCCAGGATGAGGACA | GTTCATCTCGGAGCCTGTAGTG |
| Nr1h4 (Mus musculus) | GCTTGATGTGCTACAAAAGCTG | CGTGGTGATGGTTGAATGTCC |
| NR1H4 (Homo sapiens) | AACCATACTCGCAATACAGCAA | ACAGCTCATCCCCTTTGATCC |
| Cxcl15 | TGTTGAGCATGAAAAGCCTCTAT | AGGTCTCCCGAATTGGAAAGG |
| Ccl2 | TTAAAAACCTGGATCGGAACCAA | GCATTAGCTTCAGATTTACGGGT |
| Cxcl1 | ACTGCACCCAAACCGAAGTC | TGGGGACACCTTTTAGCATCTT |
| Ccl1 | TGCCGTGTGGATACAGGATG | GTTGAGGCGCAGCTTTCTCTA |
| Cxcl2 | CCAACCACCAGGCTACAGG | GCGTCACACTCAAGCTCTG |
| Ccl25 | GAGGGCGATGAGAATCTTGAC | TCCTCACGCTTGTACTGTTGG |
| Cxcr1 | TGCTGGTTATCTTATACAGGCGA | CAGCCCTTCAATTTGGAGACA |
| Ccr8 | CGTGGGCTGCAAGAAACTGA | AGAGACCACCTTACACATCGC |
| Cxcr2 | GCCCTGCCCATCTTAATTCTAC | ACCCTCAAACGGGATGTATTGT |
| Ccr9 | GTCTCAGTTCCCCTACAACTCC | CGGAATCTCTCGCCAACAAAA |
| Ccr2 | ATCCACGGCATACTATCAACATC | TCGTAGTCATACGGTGTGGTG |

**Table S2 Description of Occlusion patients**

| Patient | Age (years) | Sex | Diagnosis | ALT(U/L) | AST (U/L) | Occlusion time (min) |
| --- | --- | --- | --- | --- | --- | --- |
| P1 | 58 | Female | FNH | 100.6 | 82.3 | 0 |
| P2 | 47 | Female | Hemangioma | 85.1 | 66.3 | 0 |
| P3 | 36 | Female | Hepatolithiasis | 102.9 | 100.9 | 0 |
| P4 | 54 | Female | Hemangioma | 207.8 | 126.5 | 15 |
| P5 | 51 | Female | Hepatolithiasis | 210.4 | 222.7 | 25 |
| P6 | 51 | Female | Angiomyolipoma | 288.3 | 240.7 | 36 |
| P7 | 52 | Female | Hepatolithiasis | 196.7 | 165.4 | 51 |
| P8 | 30 | Female | Hemangioma | 61 | 124.9 | 56 |
| P9 | 57 | Male | Hemangioma | 674.7 | 241.8 | 58 |
| P10 | 52 | Female | Hemangioma | 363.1 | 273.5 | 60 |
| P11 | 47 | Female | Hepatolithiasis | 205.6 | 151.7 | 100 |
| P12 | 70 | Male | Hepatolithiasis | 438.3 | 390.3 | 67 |
| P13  P14  P15  P16  P17  P18  P19  P20  P21  P22  P23  P24  P25  P26  P27  P28  P29  P30  P31  P32 | 53  72  24  56  45  47  24  52  26  51  28  25  45  33  39  59  38  68  47  36 | Female  Female  Female  Female  Female  Female  Male  Female  Male  Female  Male  Female  Male  Female  Female  Male  Female  Male  Female  Female | Hepatolithiasis  Hepatolithiasis  FNH  Hemangioma  Hepatolithiasis  Hemangioma  Hemangioma  Hemangioma  Hemangioma  Hemangioma  Hemangioma  Hemangioma  Hemangioma  Hepatolithiasis Hemangioma  Hemangioma  Hemangioma  Hepatolithiasis  Hemangioma  Hemangioma | 419.8  376  104.5  293.9  135  75.5  385  66.3  172.6  176.1  138.9  78.8  206.3  74.8  231.6  245.8  132  149.5  109.2  70.4 | 372.9  436.4  87.5  229.9  124  56.3  267.8  76.6  124.8  153.8  112.1  71.5  139  59.2  211.4  159.6  110.1  188.2  167.1  46.2 | 60  69  0  22  20  27  30  0  15  0  0  0  22  0  60  45  0  45  30  15 |

FNH focal nodular hyperplasia. Liver enzymes were based on the first detection post operation.
